# Behavioral state coding by molecularly defined paraventricular hypothalamic cell type ensembles

**DOI:** 10.1126/science.abb2494

**Published:** 2020-10-16

**Authors:** Shengjin Xu, Hui Yang, Vilas Menon, Andrew L. Lemire, Lihua Wang, Fredrick E. Henry, Srinivas C. Turaga, Scott M. Sternson

**Affiliations:** 1 Janelia Research Campus, Howard Hughes Medical Institute, Ashburn, VA 20147, USA.; 2 Dominick P. Purpura Department of Neuroscience, Albert Einstein College of Medicine, Bronx, NY 10461, USA.

## Abstract

**INTRODUCTION:**

Brain function is often compared to an orchestral ensemble, where subgroups of neurons that have similar activity are analogous to different types of instruments playing a musical score. Brains are composed of specialized neuronal subtypes that can be efficiently classified by gene expression profiles measured by single-cell RNA sequencing (scRNA-seq). Are these molecularly defined cell types the “instruments” in the neural ensemble? To address this question, we examined the neural ensemble dynamics of the hypothalamic paraventricular nucleus (PVH), a small brain region that is important for behavior states such as hunger, thirst, and stress. Past work has emphasized specialized behavioral state–setting roles for different PVH cell types, but it is not clear whether the dynamics of the PVH ensemble support this view.

**RATIONALE:**

We considered three possibilities for how PVH neurons could be involved in encoding behavioral states: (i) PVH neurons of a molecularly defined cell type may respond similarly and be specialized for a behavioral state as a “labeled-line,” (ii) molecularly defined cell types may show unrelated activity patterns and be irrelevant to behavioral state coding, and (iii) molecularly defined neurons may respond similarly within a type, but behavioral state may be encoded by combinations of cell types. To evaluate the role of molecularly defined cell types in the neural ensemble, it is important to monitor activity in many individual neurons with subsecond temporal resolution along with quantitative gene expression information about each cell. For this, we developed the CaRMA (calcium and RNA multiplexed activity) imaging platform in which deep-brain two-photon calcium imaging of neuron activity is performed in mice during multiple behavioral tasks. This is followed by ex vivo multiplexed RNA fluorescent in situ hybridization to measure gene expression information in the in vivo–imaged neurons.

**RESULTS:**

We simultaneously imaged calcium activity in hundreds of PVH neurons from 10 cell types across 11 behavioral states. Within a molecularly defined cell type, neurons often showed similar activity patterns such that we could predict functional responses of individual neurons solely from their quantitative gene expression information. Behavioral states could be decoded with high accuracy based on combinatorial assemblies of PVH cell types, which we called “grouped-ensemble coding.” Labeled-line coding was not observed. The neuromodulatory receptor gene *neuropeptide receptor neuropeptide Y receptor type 1* (*Npy1r*) was usually the most predictive gene for neuron functional response and was expressed in multiple cell types, analogous to the “conductor” of the PVH neural ensemble.

**CONCLUSION:**

Our results validated molecularly defined neurons as important information processing units in the PVH. We found correspondence between the gene expression hierarchies used for molecularly defined cell type classification and functional activity hierarchies involving coordination by neuromodulation. CaRMA imaging offers a solution to the problem of how to rapidly evaluate the function of the panoply of cell types being uncovered with scRNA-seq. CaRMA imaging bridges a gap between the abstract digital elements typically described in systems neuroscience with the “wetware” associated with traditional molecular neuroscience. Merging these two areas is essential to understanding the relationships of gene expression, brain function, behavior, and ultimately neurological diseases.

## Abstract (summary)

Brains encode behaviors using neurons amenable to systematic classification by gene expression. The contribution of molecular identity to neural coding is not understood because of the challenges involved with measuring neural dynamics and molecular information from the same cells. We developed CaRMA (calcium and RNA multiplexed activity) imaging based on recording in vivo single-neuron calcium dynamics followed by gene expression analysis. We simultaneously monitored activity in hundreds of neurons in mouse paraventricular hypothalamus (PVH). Combinations of cell-type marker genes had predictive power for neuronal responses across 11 behavioral states. The PVH uses combinatorial assemblies of molecularly defined neuron populations for grouped-ensemble coding of survival behaviors. The neuropeptide receptor neuropeptide Y receptor type 1 (Npy1r) amalgamated multiple cell types with similar responses. Our results show that molecularly defined neurons are important processing units for brain function.

## Graphical Abstract

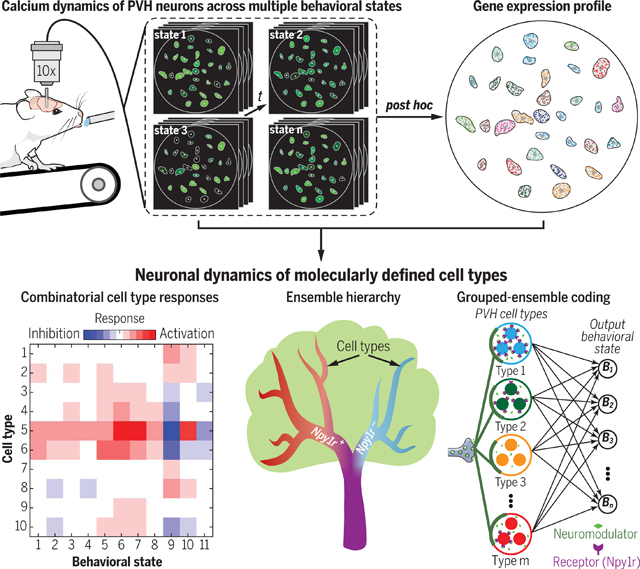

**CaRMA imaging reveals combinatorial cell type coding of behavior states.** CaRMA imaging records calcium dynamics of PVH neurons across multiple behavioral states followed by gene expression profiling. Combinatorial assemblies of PVH cell types encoded behavioral states. The PVH neural activity ensemble was split by Npy1r expression into two main cell classes that were subdivided into cell types. Thus, neuromodulation coordinates cell types for grouped-ensemble coding to represent different survival behaviors such as eating, drinking, and stress.

Neuron activity and neuronal gene expression are considered distinct facets of brain function. Large-scale neuron activity recordings reveal discrete groups of neurons that respond similarly (1–4), and gene expression brain atlases show differential expression patterns across the brain (5). Single-cell transcriptomic studies reveal that the brain is composed of hundreds of molecularly defined neuronal subtypes (6–8). Molecular markers facilitate repeatedly returning to the same type of neurons and also serve as genetic elements that can be used for targeting functionally similar neurons for causality testing (9, 10) or potentially therapeutics (11). These applications can only be justified if molecular markers categorize neurons into groups with similar neuronal dynamics (12) that together encode distinct behavioral states. However, there is limited knowledge about the relationship between activity patterns of cells in the neural ensemble and their underlying gene expression profiles. Therefore, the merit of using neuronal gene expression as a proxy for neurons with similar activity patterns is controversial (12–17).

We set out to determine (i) the role of molecularly defined cell types for encoding different behavioral states and (ii) the association of neuronal marker genes with distinct functional response types of the neuronal ensemble from the mouse paraventricular hypothalamus (PVH). The PVH mediates appetite, stress, thirst, and autonomic functions. This brain region is well known for its diverse peptide-expressing cell types (18, 19), and there are distinct behavioral and physiological consequences of PVH neuropeptide pharmacology (20) as well as cell type–selective optogenetic and chemogenetic perturbations (21–23). Molecular and cellular perturbation studies have indicated that the PVH acts as a behavioral state “switchboard” in which each molecularly defined population influences a distinct behavioral state as a labeled-line (24). However, it is not known if this is consistent with the natural dynamics of PVH neurons during these behaviors.

We considered three models for the role of molecularly defined cell types to encode behavioral states. The labeled-line model encodes different behavioral states based on the activity pattern of distinct, molecularly defined cell types (Fig. 1A). Labeled-line coding is frequently assumed for information processing in hard-wired neural functions involving essential survival behaviors (25–27). At the other end of the spectrum, the full neural ensemble encodes behavioral state irrespective of cell type identity and is primarily a product of neural plasticity (Fig. 1B) (28). In this case, molecular identity is a poor predictor of behavioral state, and functional ensembles are formed using rules that are independent of cell type. An intermediate model is grouped-ensemble coding, in which the members of some molecularly defined cell types show coordinated responses and encode behavioral states based on specific combinations of cell types (Fig. 1C).

It has been extremely difficult to distinguish these models because there has not been a suitable approach to simultaneously examine the functional dynamics of individual neurons having distinct molecular identities across many behavioral states within a single animal. Several methods have been developed to map gene expression onto neuronal function (29–33). However, these methods have limitations of temporal sensitivity, dynamic range, molecular diversity, or restrictions to transparent organisms that have prevented evaluation of this type of problem. Thus, an unbiased and systematic method to relate multigene expression of individual neurons to their activity patterns in vivo on a subsecond time scale is needed to combine molecular and systems neuroscience.

To address this problem, we developed the CaRMA (calcium and RNA multiplexed activity) imaging platform, a comprehensive, three-part strategy compatible with recording neuron dynamics deep in the mouse brain (Fig. 1D). In the first step, we used single-cell RNA sequencing (scRNA-seq) to classify PVH cells into molecular types (Fig. 1D, a and b). In the second step, we performed volumetric, deep-brain, pan-neuronal, two-photon calcium imaging in awake, behaving animals without regard to PVH neuronal subtypes to measure the response dynamics of hundreds of neurons during multiple behavioral states (Fig. 1D, c). Subsequently, the brain was removed, sectioned, and the neurons in the ex vivo tissue sections were registered to those from the in vivo imaging volume (Fig. 1D, d and e). In the final step, molecular identity of the neurons imaged in vivo was determined post hoc by performing multiple rounds of three-plex RNA-fluorescent in situ hybridization (FISH) on the ex vivo tissue sections, guided by marker genes from scRNA-seq in step 1 (Fig. 1D, f to i). This approach enables monitoring the dynamics of many molecularly defined neurons in parallel, within a deep-brain region, and across multiple behaviors.

## RESULTS

### Molecularly defined cell types within PVH

#### scRNA-seq of PVH neurons

We used scRNA-seq to transcriptionally profile 706 manually picked PVH cells from 10 animals. We applied iterative unsupervised clustering (8) to group cells into 12 molecularly defined cell types within the PVH (fig. S1A). Differentially expressed marker genes for these clusters included canonical PVH neuropeptides: oxytocin (*Oxt*), vasopressin (*Avp*), corticotropin-releasing hormone (*Crh*), thyrotropin-releasing hormone (*Trh*), somatostatin (*Sst*), proenkephalin (*Penk*), and prodynorphin (*Pdyn*). Within these broad classes, clusters were further divided into subgroups based on the expression of additional marker genes: glutamic acid decarboxylase 2 (*Gad2*), reelin (*Reln*), netrin G1 (*Ntng1*), and neuropeptide Y receptor type 1 (*Npy1r*) (fig. S1B). All PVH clusters expressed *Sim1*, contained the excitatory neuron marker *Vglut2* (*Slc17a6*), and lacked the inhibitory neuron marker *Vgat* (*Slc32a1*). This targeted, deep-sequencing approach provides a combinatorial set of marker genes for molecularly defined cell type assignment.

#### 12-plex FISH in PVH

We mapped the spatial and coexpression pattern of these 11 differentially expressed genes plus *Vglut2* by performing four rounds of three-plex FISH from two mice in PVH subregions separated by 280 mm: the anterior PVH (aPVH, 1394 cells), middle PVH (mPVH, 1855 cells), and posterior PVH (pPVH, 1263 cells) (Fig. 2A). This was achieved by developing new methods for stripping fluorescently labeled probes, aligning individual cells in three dimensions across multiple rounds of FISH using 4′,6-diamidino-2-phenylindole (DAPI) fluorescence, as well as generalizable methods for three-dimensional (3D) segmentation of cell boundaries (figs. S2 to S4; see the materials and methods).

We observed marker gene enrichment in different PVH subregions (Fig. 2B): Two genes (*Gad2 and Ntng1*) were significantly enriched in aPVH, three genes (*Vglut2*, *Crh*, and *Avp*) were significantly enriched in mPVH, and nine genes (*Vglut2*, *Gad2*, *Npy1r*, *Crh*, *Reln*, *Ntng1*, *Pdyn*, *Oxt*, and *Avp*) were significantly enriched in pPVH. Pairwise gene coexpression also differed based on anterior-posterior position (fig. S5). For example, a majority of *Crh* cells coexpressed *Npy1r* in pPVH (75%) but considerably less in mPVH (30%) and aPVH (35%).

We determined the proportions of PVH cells in situ that expressed 0 to 12 of the marker genes (Fig. 2C). Coexpression (more than two genes) was common (81% of cells), and the mode was three genes. Unsupervised hierarchical clustering of gene expression profiles from these 4512 PVH cells resulted in 13 transcriptional clusters (Fig. 2, D to F). The spatial distribution of these molecularly defined cell types was largely intermingled but some well-organized patterns were apparent (Fig. 2G; fig. S6, A and B; and table S1). Expression levels of the marker genes were distributed continuously, with strong rightward skew and high variance (Fig. 2H). Clustering implicitly thresholded gene coexpression relationships, and most FISH clusters (12/13) were dominated by high expression of one gene (Fig. 2E). FISH clusters were correlated with the scRNA-seq dataset (fig. S6, C and D). Likewise, scRNA-seq clusters were represented in the FISH dataset (fig. S6, E to G) and mapped to different regions of the PVH (fig. S6G).

#### CaRMA imaging

To record PVH neuronal dynamics, we expressed GCaMP6m in PVH neurons without regard to neuronal subtypes. A thin gradient refractive index (GRIN) lens was implanted into the PVH (Fig. 3A). Volumetric two-photon calcium imaging was acquired from PVH neurons under the GRIN lens (range: 80 to 240 μm) in a head-fixed behaving mouse (Fig. 3B). We performed a set of calcium-imaging experiments involving eating during hunger, drinking water during thirst, hedonic eating (ad libitum fed, palatable food), fear retrieval (movie S1), and hormone-induced hunger (ghrelin) or energy surfeit (leptin) sequentially over 10 days (fig. S7). Subsequently, the brain was removed and sectioned.

To register the GCaMP-expressing neurons in the ex vivo–sectioned brain to the in vivo image volume, the sections below the GRIN lens were imaged by confocal microscopy. Because the GRIN lens introduced multiple optical distortions, we characterized these aberrations and transformed the in vivo image volume coordinates to the view obtained by confocal imaging in ex vivo tissue sections (fig. S8). For a successful in vivo ↔ ex vivo alignment, it was crucial to correct field curvature introduced by the GRIN lens (fig. S8, C, D, F, and J), as well as the nonlinear relationship between sample position and objective lens position (fig. S8, H and J) and depth-dependent magnification changes (fig. S8, C, D and I). After correcting for these aberrations (Fig. 3C and fig. S9A), we found neurons with distinctive shapes in the two-photon–imaging volume (neurons 1 to 3 in Fig. 3D; neurons 1, 2, 4, and 35 in fig. S9B) that could be recognized in the ex vivo confocal images. We used these neurons as starting points to match the surrounding neurons imaged in vivo and ex vivo with 96% correspondence between two independent observers. Most of the neurons (89.3%, 334/374) imaged in vivo could be found in the brain slices, and 95.5% (319/334) of the aligned neurons could be tracked across the entire sequence of experiments (Fig. 3D and fig. S9B).

After matching neurons between the in vivo and ex vivo image spaces, we quenched GCaMP6m with acidic buffer (34) and performed four rounds of three-plex RNA-FISH (Fig. 3E and fig. S9C). We developed a non-rigid 3D registration pipeline for the GCaMP-expressing brain slices across multiple rounds of processing (fig. S10). Calcium dynamics of individual neurons during multiple behaviors were extracted from the in vivo image volumes (fig. S11), along with the corresponding molecular profiles for each neuron acquired from ex vivo tissue sections (Fig. 3F and fig. S9D). These computational and image analysis tools are available online (see “Data and materials availability” in the Acknowledgments).

#### PVH ensemble activity across 11 behavioral states

We recorded calcium dynamics of 319 neurons from three mice in the same portion of the pPVH (fig. S12) during all behaviors. We segmented the behavioral tasks into 11 behavioral states (fig. S7). These behavioral states could be well separated by unsupervised clustering of the recorded PVH neural activity patterns (Fig. 4A).

#### Labeled-line coding

First, we investigated whether this PVH ensemble showed evidence for labeled-line coding of behavioral states irrespective of any gene expression information. A labeled-line neuron was defined as specifically activated or inhibited in one state but not in other states. We searched for the maximum number of labeled-line neurons among all *k* combinations of 11 behavioral states (Fig. 4B). Although selectively tuned neurons were evident with comparison of a small number of behavioral states, the number of neurons showing apparent labeled-line coding was dependent on the number of behavioral states examined (Fig. 4C). Beyond six behavioral statessss, there were no selective neurons for each state. Moreover, the proportions of neurons selective for each behavioral state were statistically greater than chance for not more than four states (Fig. 4B). We also considered the possibility that grouping similar states might make labeled-line coding more apparent. States were grouped into “state classes” either subjectively based on ideas about behavioral similarity (fig. S13A) or systematically based on PVH neuronal response similarity metrics for unsupervised clustering (Fig. 4A). However, grouping further reduced performance of the labeled-line model (Fig. 4D and figs. S13 and S14). Examining a small number of behavioral states might thus lead to the erroneous conclusion that a set of neurons is involved in highly selective labeled-line coding. Although the labeled-line configuration cannot be excluded for some brain functions, labeled-line coding is poorly scalable.

#### Molecularly defined neural ensemble responses

Next, we used unsupervised clustering of gene expression profiles to group the PVH neurons into 11 molecularly defined clusters (MCs) (Fig. 5, A to C; see the materials and methods) so that we could evaluate the activity patterns in molecularly defined cell types across 11 behavioral states. Although GCaMP expression was not observed in *Oxt-* or *Avp*-expressing neurons because of adeno-associated virus tropism (fig. S15), these 11 MCs were consistent with cell types clustered from FISH-only pPVH tissue (fig. S16). Spatially, these molecularly defined cell types were intermingled, primarily in the medial parvicellular and lateral parvicellular subdivisions of the pPVH (Fig. 5, D and E).

#### Response purity within molecular clusters

If cell type information is important to encode a behavioral state, then we expected that neurons within a molecularly defined type, grouped solely using gene expression information, should respond similarly in that state. When we looked at MC5-Crh neurons in response to hedonic eating and fear retrieval, we found that neurons within this cluster responded similarly. By contrast, MC8-Penk neurons showed highly heterogeneous responses in these states (Fig. 5F). Thus, molecularly defined neurons do not necessarily respond similarly in all behaviors and may be tuned to only some behavioral states.

We quantified the similarity of neuronal responses within each cell type cluster using linear purity (fig. S17; see the materials and methods). During fear cue presentation, MC5-Crh had high purity (approaching 1), whereas MC8-Penk had low purity (close to zero; Fig. 5F). Cell types with purity >0.5 can be considered to respond similarly (fig. S17). Next, we defined a metric called “consistent-response”, which weights cell type activity by the response purity (Fig. 5F and fig. S17). A high consistent-response molecularly defined cell type is composed of neurons that respond strongly to a stimulus with similar temporal dynamics. Low consistent-response cell types have either low purity (a diversity of responses) or low response magnitude in a behavioral state (fig. S17).

#### Combinatorial molecularly defined cell type tuning to behavioral states

We computed the temporal maximum of consistent-responses and purities of each cell type in 11 behavioral states (Fig. 5H and fig. S18, B and C). In the consistent-response map, some cell types responded differently to individual behavioral states, whereas others were similarly tuned across multiple states. MC8-Penk neurons, which had relatively low response and low purity during fear, showed higher purity activation after injection of the hormone ghrelin. This demonstrates that molecularly defined neurons are tuned to respond similarly in some behaviors but not others. The purity of the molecularly defined cell types resulting from unsupervised clustering of multiple genes produces significantly higher response purities than when cells are grouped by expression of a single gene (fig. S19, A to C). The high purity activation response to food consumption of MC5-Crh neurons that highly express *Crh* and coexpress *Vglut2* and *Npy1r* in the pPVH is opposite to the inhibitory response reported by low-resolution, multineuron fiber photometry measurements of all CRH neurons in the PVH, although both methods give the same response to fear-inducing stimuli (35, 36). However, low-expressing *Crh*^+^/*Vglut2*^+^/*Npy1r*^−^ neurons in the pPVH were inhibited in response to food ingestion (fig. S20), and this cell type is more prevalent in the mPVH (fig. S20, F and G), which is the major target region in the reported photometry experiments (35, 36). Thus, CaRMA imaging is a powerful platform for systematically discovering the relationship between cell types defined by multiple gene markers and their response consistency in different behavioral states.

The consistent-response map shows the relationships between molecularly defined cell type activity patterns for behavioral state coding (Fig. 5H). From these data, it is apparent that behavioral state is not represented by a single molecularly defined cell type (fig. S19D). Instead, the consistent-response pattern indicates combinatorial coding. What is different from previous views of hypothalamic circuits is that behavioral state is encoded by the different combinations, magnitudes, and temporal dynamics of cell type activity patterns. For example, MC5-Crh and MC6-Pdyn showed similar consistent-responses during hunger eating, drinking, and hedonic eating (absolute differences ≤0.18; Fig. 5H), but their temporal response profiles were significantly different (Fig. 5G). MC5-Crh neurons ramped to maximum magnitude, whereas MC6-Pdyn neurons responded more quickly to the ingested stimuli (Fig. 5G). Similarly, the return to baseline after withdrawing food, water, or the offset of the fear retrieval cue was slower for MC5-Crh (fig. S18A). Although these cell types have similar consistent-response measures, their different temporal dynamics indicate distinct functions for encoding behavioral states, as is also the case for other cell types (fig. S18A).

### Decoding behavioral states with molecularly defined cell types

#### Individual cell type coding

CaRMA imaging enabled us to investigate whether behavioral states can be quantitatively decoded from the PVH ensemble dynamics of molecularly defined cell types. Because cell type encoding of behavioral states has been traditionally examined either one cell type at a time (37–39) or was used without regard to cell type (2, 3, 40), we first examined the decoding performance of individual molecularly defined cell types to distinguish 11 behavioral states.

We used multinomial logistic regression classification to determine the overall performance of predicting behavioral states from the temporal dynamics of single neurons without regard to cell type (All) as well as from singlssse neurons of known cell types (Fig. 6A; see the materials and methods). Single neurons from several cell types showed decoding performance superior to a classifier trained using all neurons without regard to cell type (Fig. 6B and fig. S21A). The decoding accuracy from the dynamics of MC5-Crh neurons was the highest (Fig. 6B), and the confusion matrix for behavioral state decoded by MC5-Crh neurons showed perfect decoding of the ghrelin-induced hunger state and high decoding performance for fear retrieval (Fig. 6C). MC5-Crh neurons showed significantly different response amplitude and sign across several behavioral states (Fig. 6D), which explained the high decoding performance. However, our analysis also showed that individual cell types in the PVH are insufficient to decode many behavioral states.

#### Grouped-ensemble cell type ensemble coding

**Fig. 1.**
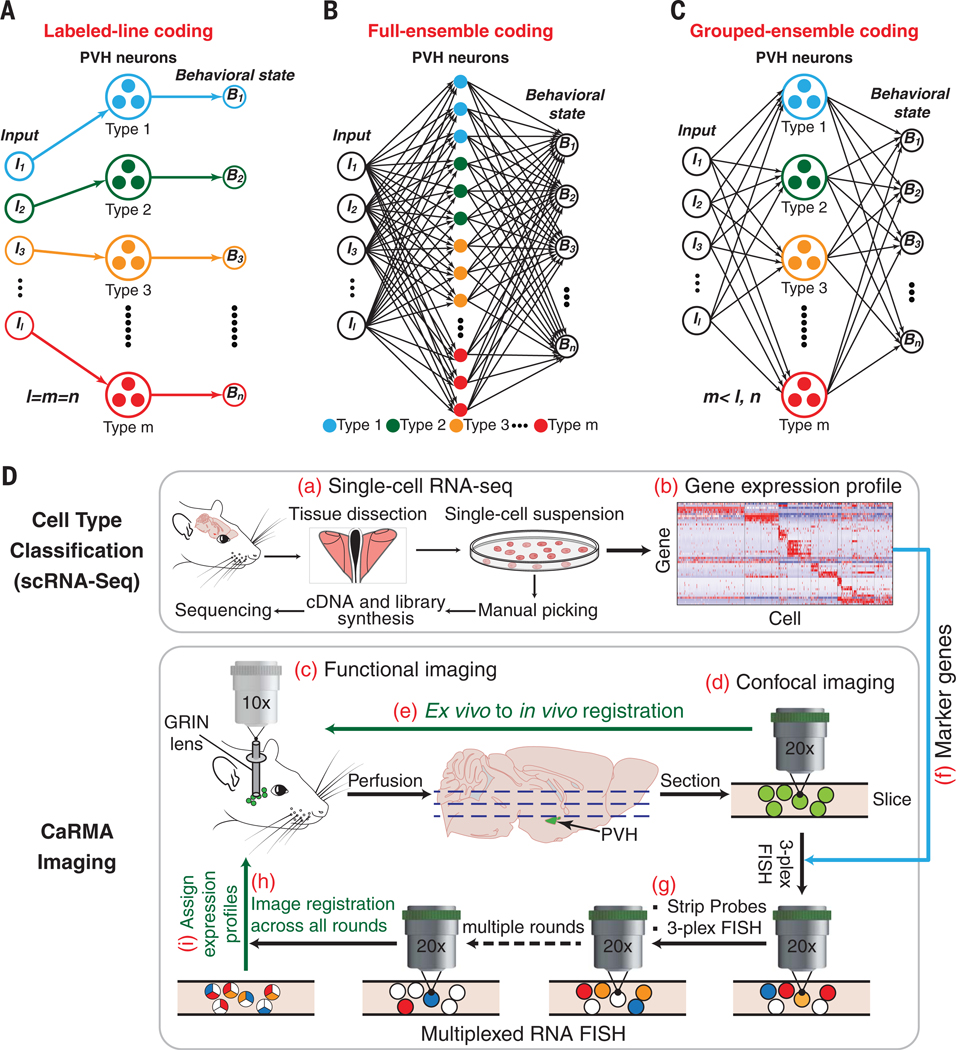
CaRMA imaging for investigating models of behavioral state coding by molecularly defined cell types. (**A** to **C**) Models of multiple molecularly defined cell types encoding multiple behavioral states after processing diverse internal and external inputs (*I*). (A) Labeled-line coding uses a specialized cell type for a behavioral state in which individual members respond similarly, where the number of encoding cell types (*m*) is equal to the number of distinctly encoded behavioral states (*n*). (B) In a full-ensemble-coding model, molecularly defined cell types do not respond similarly, and behavioral state coding is independent of cell type. (C) Grouped-ensemble coding uses combinations of molecularly defined cell types in which individual molecularly defined cell types act as a coherent functional unit. (D) Schematic of the CaRMA imaging platform.

CaRMA imaging enables the activity of many cell types to be imaged simultaneously in the same animal. This permitted us to test models of behavioral state coding using an ensemble of molecularly defined cell types (Fig. 1C) or, alternatively, an ensemble of neurons in which molecularly defined cell type information was ignored (Fig. 1B). We trained a classifier on an ensemble of molecularly defined cell types model by randomly choosing one cell’s activity trace from each of 10 molecularly defined cell types and concatenating them into 10-cell ensembles for each behavioral state (repeated 100 times with replacement), followed by multinomial logistic regression for 11 behavioral states using 10-fold cross-validation (accuracy: 93.13 ± 0.02%, Fig. 6, E and F; see the materials and methods). With molecularly defined cell type information, 11 different behavioral states could be accurately decoded with the combinatorial responses of only 10 PVH neurons, one from each of the 10 cell types. This was significantly higher decoding accuracy (*P* = 0; Fig. 6G) than a neural ensemble that ignores molecularly defined cell type information by randomly assigning neurons into dummy cell types (fig. S21B).

We also examined the effect of adjusting the hierarchical molecularly defined cell type clustering threshold on behavioral state decoding. Purity and decoding accuracy generally increased with the number of molecular clusters (figs. S22 and S23). Although grouped-ensemble coding was supported with a range of clustering thresholds, this approach involves “peeking” at the functional responses to supervise molecular clustering. Because our objective was to evaluate the predictive value of molecular information on functional and behavioral responses, we proceeded using the unbiased determination of 11 MCs in the pPVH ensemble (to avoid circular logic). Taken together, our findings support a grouped-ensemble coding strategy used by molecularly defined PVH neurons (Fig. 1C).

#### Cell type ensemble response-decoding diagrams

To reveal the relative contributions of different molecularly defined cell types to grouped-ensemble coding (Fig. 1C) for different behavioral states in the PVH, we used the multinomial regression coefficients and the consistent-response measurements to generate cell type response-decoding diagrams for each behavioral state (Fig. 6, H to K, and fig. S24). In these diagrams, cell type consistent-responses are weighted by each cell type’s contribution to decoding accuracy and quantitatively summarized across multiple behavioral states. MC5-Crh and MC6-Pdyn neurons had large consistent-responses in several behavioral states (23, 35, 36). These neurons were activated by negative and positive stimuli, indicating an unexpected general role for encoding salience of diverse stimuli in a variety of different states. Other molecularly defined cell types, such as MC8-Penk and MC10-Trh neurons, contributed substantial weights for distinguishing behavioral states (Fig. 6, H to K, and fig. S24C). For example, food withdrawal (Eat-Aft) in hunger elicited a transient inhibitory response in MC10-Trh after spout retraction that transitions to a prolonged activated response, which was not observed after removing water in thirst (Drink-Aft) (fig. S25A). This contributes to behavioral state coding (fig. S25B) and is also consistent with past work indicating that TRH neurons project to the arcuate nucleus and activate hunger-associated Agouti-related peptide (AGRP) neurons (22). By contrast, most MC8-Penk neurons showed prolonged inhibition after food withdrawal in hunger but only transient inhibition after drinking in thirst (fig. S25, C and D). More generally, these response-decoding diagrams show a functional organization in the pPVH, with major branches associated with the sign and amplitude of broadly tuned MC5-Crh and MC6-Pdyn neurons and individual twigs associated with a more selective combinatorial code of cell types that distinguish behavioral states.

#### Gene expression prediction of ensemble functional responses

Grouped-ensemble coding relies on similar responses from the neurons that make up a cell type, thereby simplifying the neural ensemble to component cell types instead of the much larger total number of cells. Consistent-response maps also indicate higher-level organization based on similar responses between groups of cell types. For example, MC5-Crh and MC6-Pdyn show similar response tuning to multiple behavioral states. By contrast, MC8-Penk typically responds oppositely to these cell types (Figs. 5H and 6, H to K, and fig. S24C). Thus, different molecularly defined cell types may have coordinated activity patterns that comprise hierarchical functional groupings in the neural ensemble. This raises the second major question: Do differentially expressed genes map to functional response types in the neural ensemble?

We aimed to identify optimal combinations of genes that best predict the functional response classes within the neuronal ensemble. Moreover, neurons have complex dynamics during behaviors, so we assessed the temporal window over which molecular markers offer useful predictive information about functional responses.

#### Differential gene expression in functional clusters

For each behavioral state, we grouped all neurons on the basis of their neuronal response dynamics into functional clusters (FCs) by unsupervised hierarchical clustering (Fig. 7, A and B, and fig. S26). Two FCs emerged for each behavioral state associated with primarily activated (Act-FC) or inhibited (Inh-FC) neurons. We first examined how functional clustering segregates neuron gene expression profiles.

A subset of gene expression profiles specifically segregated with functional response clusters (Fig. 7, A to C, and fig. S26). We performed differential gene expression analyses between the two FCs in each behavioral state (fig. S27A), which was measured by either the *P* value of the expression-level difference or by the ratio of expression level between FCs (Fig. 7D and fig. S27B). *Npy1r* was significantly enriched across 11 behavioral states and was ranked as the first or second enriched gene by *P* value. *Crh* and *Pdyn* were significantly enriched in eight and nine behavioral states, respectively. Moreover, if *Npy1r*, *Pdyn*, and *Crh* were enriched for a behavioral state, then they were found in the same FC because of partial coexpression in the same cells. For example, *Npy1r*, *Pdyn*, and *Crh* were enriched in the Act-FC for Eat-Hed (hedonic) and Fear states. By contrast, the Inh-FC was enriched with neurons expressing *Penk*. We also examined combinations of molecularly defined cell types within FCs in each behavioral state. For most behavioral states (eight of 11 states), activated or inhibited FCs also showed significant enrichment of cell types (fig. S28). The cell types with the highest FC selectivity were MC5-Crh and MC6-Pdyn, which also showed high consistent-responses in most behavioral states (Fig. 5H). These gene and cell type enrichment analyses demonstrated a strong association between neuron function during behavior and the molecular profile of individual neurons. However, this analysis lacks useful metrics for ranking the predictive power of gene expression for functional response class, and it does not define the optimal set of marker genes for predicting responses across the neural ensemble.

#### Gene expression prediction of FCs

**Fig. 2.**
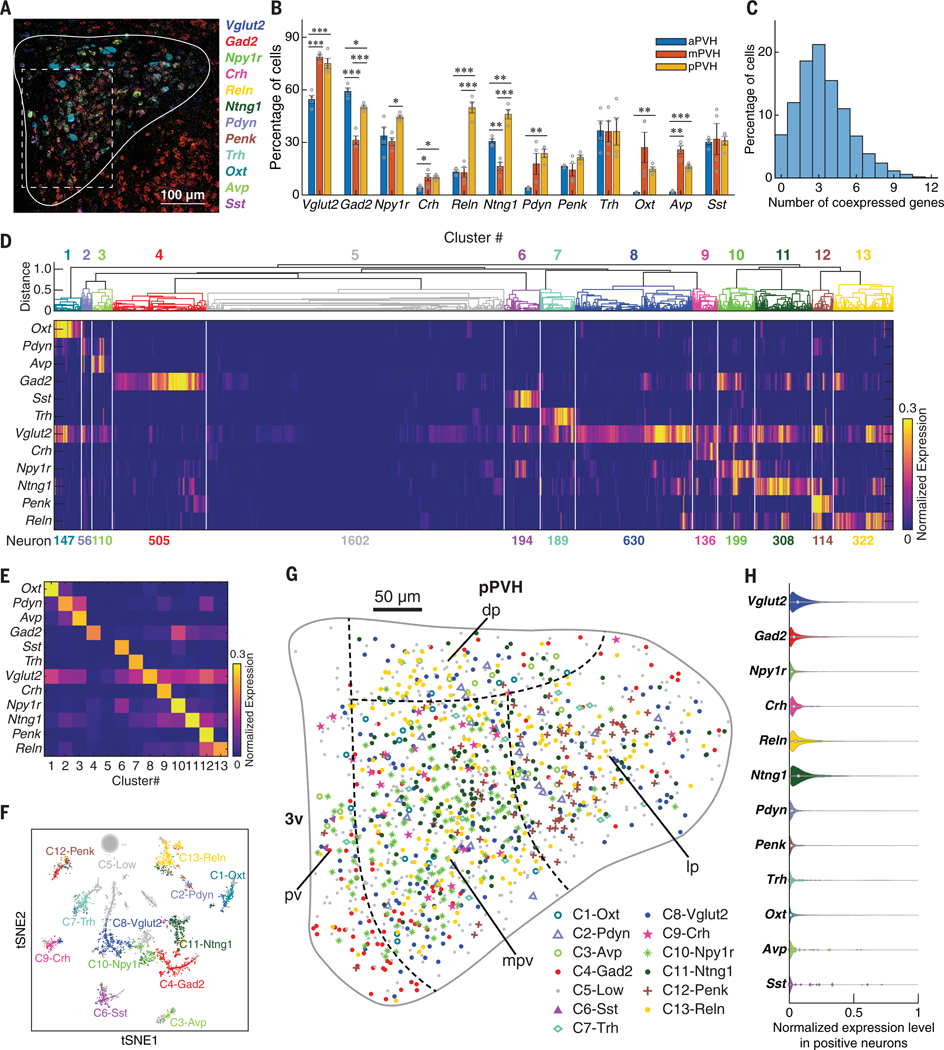
Molecular and spatial characterization of PVH neurons by multiplexed FISH with 12 marker genes. (**A**) Maximum intensity projection image of multiplexed FISH with 12 marker genes in pPVH. Right, mRNA puncta pseudocolor legend. Solid white line is the contour of pPVH; dashed white line is the region in fig. S4B. (**B**) Percentages of cells expressing each marker gene in aPVH, mPVH, and pPVH. Error bars indicate mean ± SEM. Gray circles are sample data. *P < 0.05, **P < 0.01, ***P < 0.001. Statistics are provided in table S2. (**C**) Histogram of PVH cells coexpressing various number of marker genes. (**D**) Gene expression profiles of 13 molecularly defined PVH cell types from hierarchical clustering of normalized expression of 12 marker genes. (**E**) Mean expression pattern of marker genes in 13 cell types. (**F**) Cell types in (D) plotted by *t*-distributed stochastic neighbor embedding (tSNE). “Cell types” are transcriptional clusters denoted as C*i*-*xxx*, where *i* is the cluster number in (D) and *xxx* is a highly expressed gene or “Low” (low expression for all probed genes). (**G**) Spatial organization of 13 molecularly defined cell types in pPVH (data are from four samples). Each symbol represents one neuron. PVH subregion boundaries are from reference (55). pv, periventricular; dp, dorsal parvicellular; lp, lateral parvicellular; mpv, medial parvicellular ventral zone; 3V, 3rd ventricle. (**H**) Normalized expression levels of marker genes from PVH neurons.

**Fig. 3.**
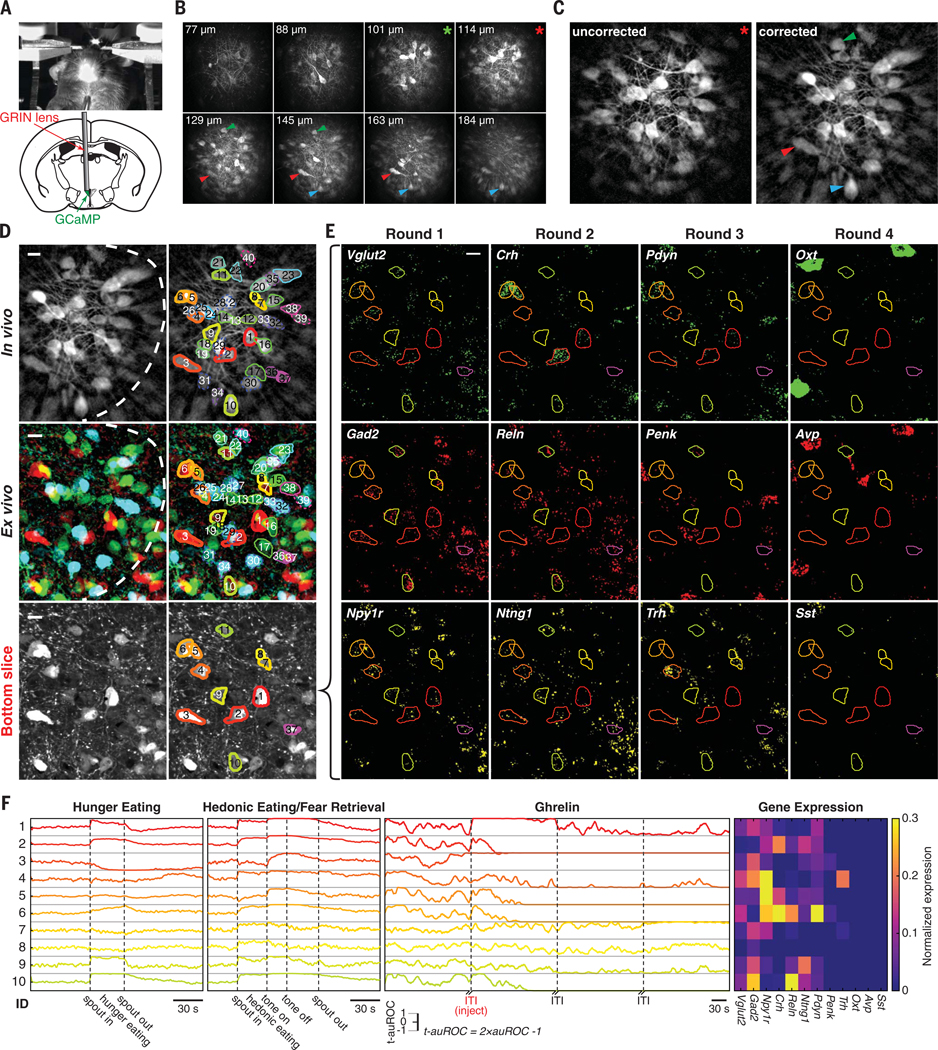
Ex vivo ↔ in vivo registration for CaRMA imaging. (**A**) Top, head-fixed mouse during two-photon calcium imaging of PVH neurons through a GRIN lens. Bottom, schematic of GRIN lens targeting GCaMP-expressing PVH neurons. (**B**) Eight planes from a two-photon imaging volume during behavior. Upper left, distances between imaging planes and GRIN lens. Arrowheads mark the corresponding neurons in (C). Red or green asterisks indicate the imaging plane in Fig. 3C or fig. S9A, respectively. (**C**) Computational correction of optical aberrations from in vivo imaging. Arrowheads indicate neurons from deeper imaging planes in (B) because of field-of-view (FOV) curvature correction. (**D**) Example neurons showing the ex vivo registration to a substack of the in vivo image volume. Ex vivo image is overlay of *z*-projected confocal stacks from three consecutive 14-μm brain slices (pseudocolors: cyan, green, and red indicate top, middle, and bottom slices). White dashed line is the resolvable in vivo FOV. Dashed, thin, and thick contours are neurons from the top, middle, and bottom slices, respectively. (**E**) Four rounds of three-plex FISH from neurons in the bottom slice in (D). (**F**) Calcium dynamics of neurons in (E) across multiple behavioral states and their 12-plex gene expression profiles. t-auROC, transformed auROC (see the materials and methods); ID, neuron number from (D); ITI, intertrial interval (2 min). Scale bars in (D) and (E), 15 μm.

**Fig. 4.**
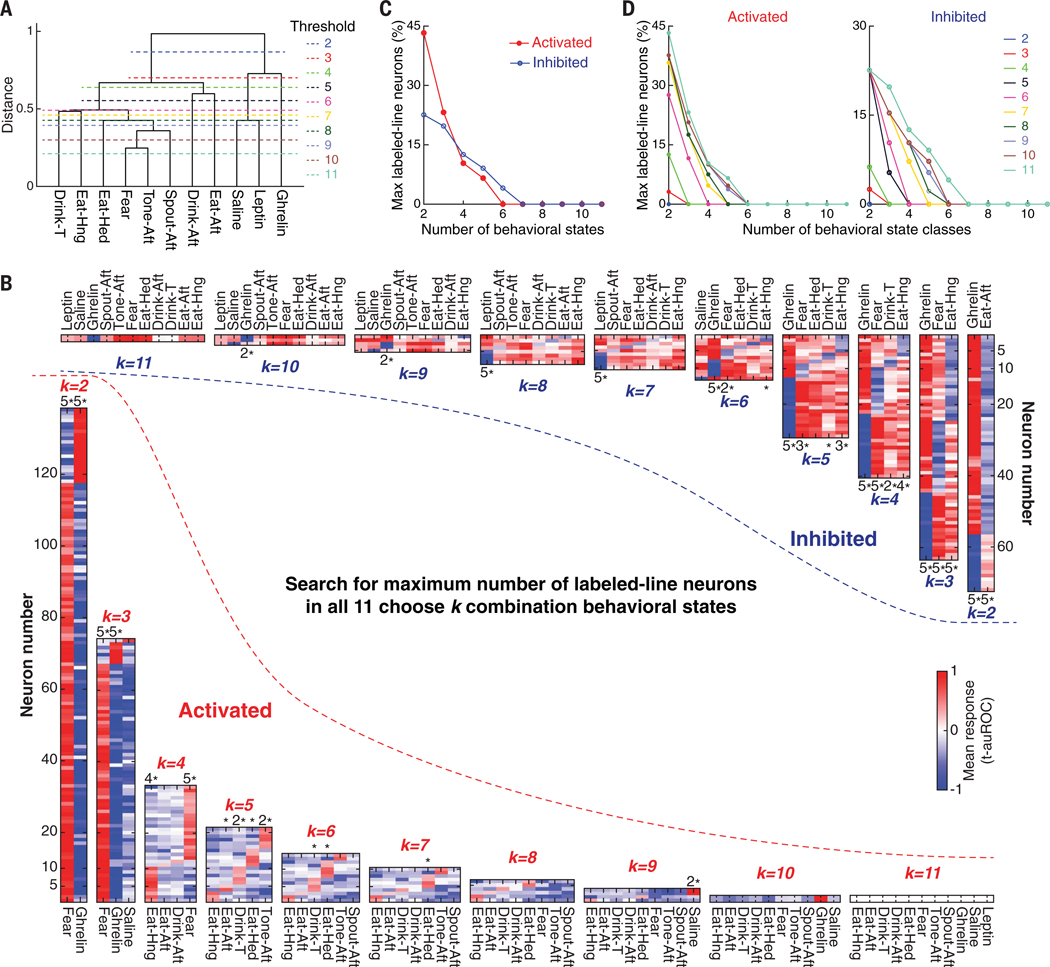
Screening for labeled-line neurons encoding multiple behavioral states. (**A**) Dendrogram of neuron ensemble response similarities across 11 behavioral states. Dashed lines are thresholds for grouping state classes. (**B**) Mean response maps of the maximum number of labeled-line neurons in all 11-choose-***k*** combinations of behavioral states. Bottom left, Activated labeled-line neuron sets. Top right, inhibited labeled-line neuron sets. Fisher’s exact test was used to evaluate whether neurons are significantly specialized for a behavioral state. (**C**) Number of labeled-line neurons depends on the number of behavioral states. (**D**) Number of labeled-line neurons depends on the number of behavioral state-classes [see (A)]. *, *P* < 0.05; 2*, *P* < 0.01; 3*, *P* < 0.001; 4*, *P* < 0.0001; 5*, *P* < 0.00001.

**Fig. 5.**
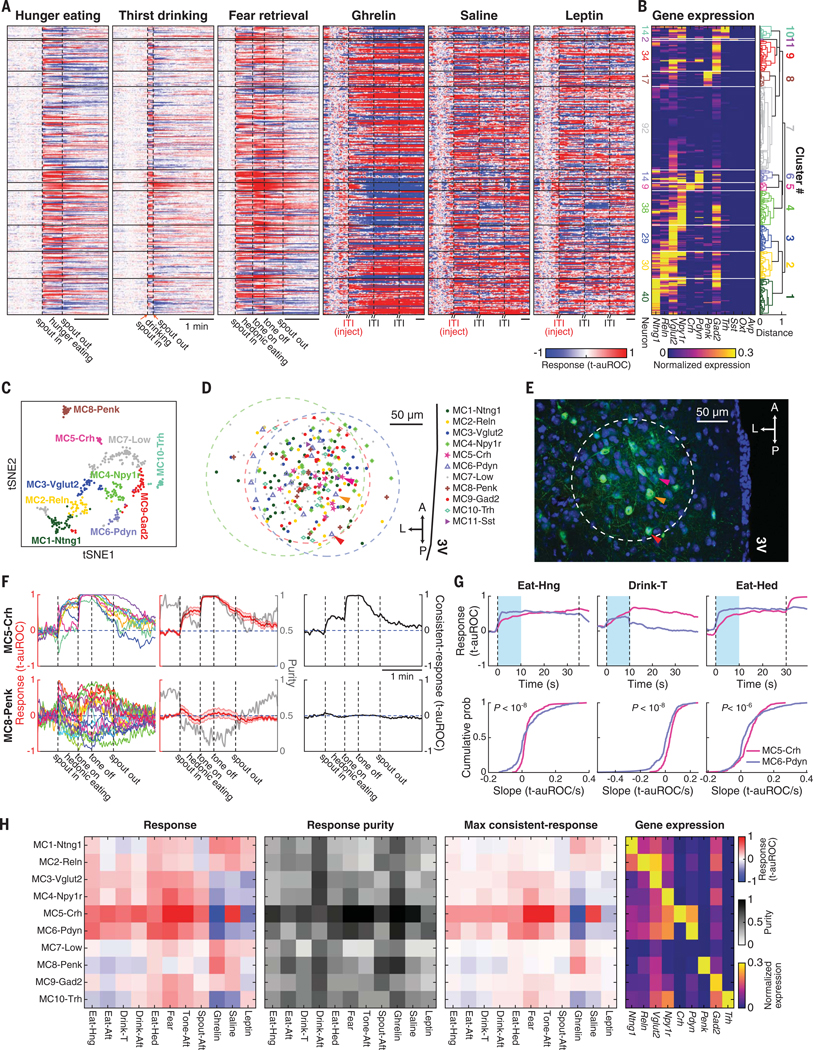
Calcium dynamics and gene expression profile of PVH neurons across 11 behavioral states. (**A**) Calcium response dynamics of the same PVH neurons (319 cells) during multiple behaviors from three mice. Neurons are clustered by gene expression profiles in (B). Temporal scale bars, 1 min. (**B**) Gene expression profile (12-plex RNA-FISH) of neurons in (A). Molecularly defined cell types are clustered based on the expression of the first nine genes. Cluster 11 has two neurons with high *Sst* expression even though *Sst* was not used for clustering (excluded for later analysis because of low neuron count; see the materials and methods). (**C**) Cell types in (B) plotted by tSNE. “Molecular clusters” are denoted as MC*i*-*xxx*, where *i* is the cluster number in (B) and *xxx* is a highly expressed gene or “Low” (low expression for all probed genes). (**D**) Spatial distribution of these cell types in the three imaging FOVs (dashed circles). Colored arrowheads indicate the corresponding neurons in (E). (**E**) Fluorescence image of GCaMP-expressing example neurons within a FOV (dashed circle). Blue is DAPI and green is GCaMP. (**F**) Responses of MC5-Crh and MC8-Penk neurons during fear retrieval. Left, response traces of individual neurons. Middle, red shaded lines are the mean responses ± SEM across neurons; gray lines are purities. Right, instantaneous consistent-responses. (**G**) Different response temporal profiles of MC5-Crh and MC6-Pdyn neurons. Top, Mean responses aligned with food or water presentation. Bottom, Cumulative distribution of instantaneous response slopes of individual neurons from these cell types during the light-blue-shaded periods from the top panel (two-sample Kolmogorov-Smirnov test). (**H**) Temporal maximum for consistent-response and corresponding response and purity of PVH cell types defined by combinatorial gene expression profiles across 11 behavioral states.

**Fig. 6.**
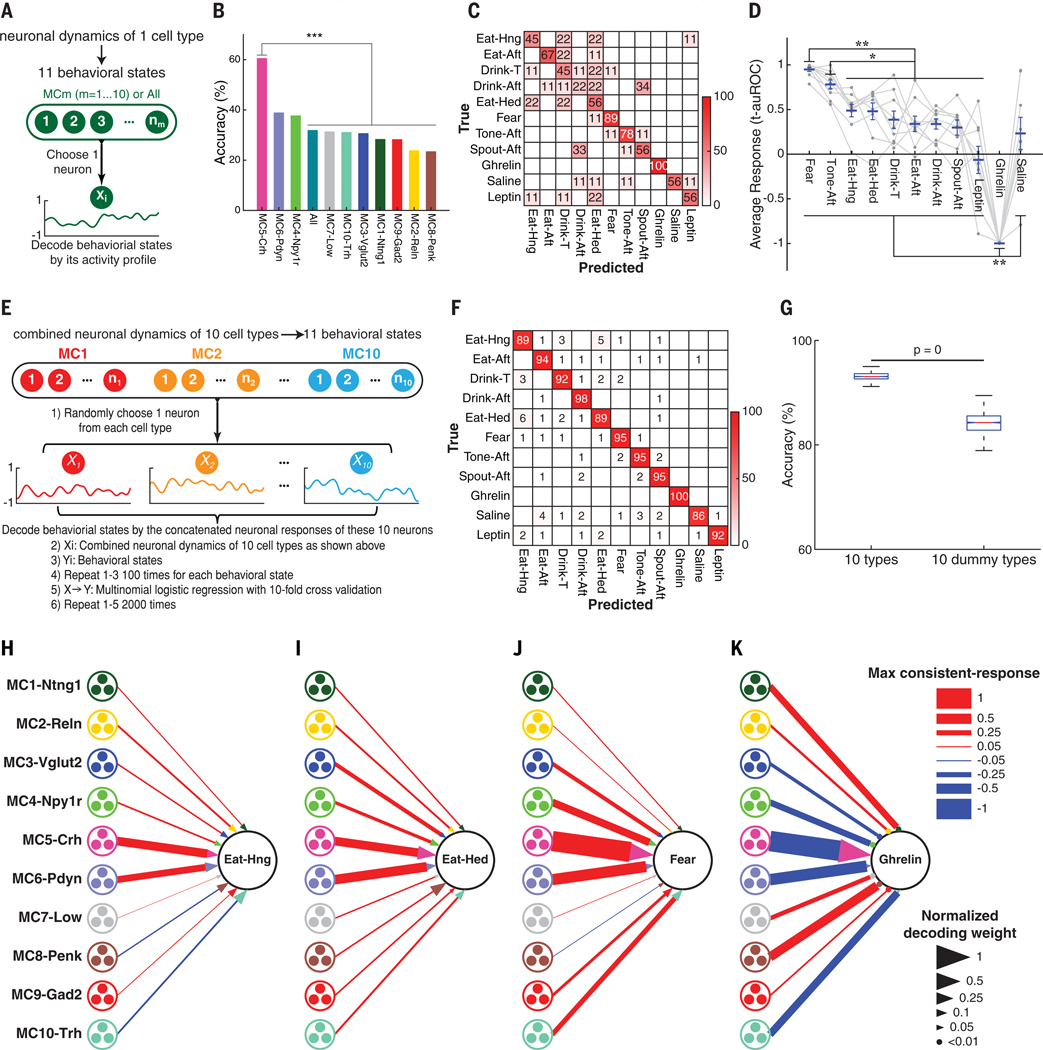
Decoding behavioral states with neuron dynamics of molecularly defined PVH cell types. (**A**) Schematic procedure for decoding behavioral states with the temporal dynamics from individual cell types or all neurons (All, disregards cell type). (**B**) Decoding accuracies for all behavioral states using the temporal dynamics of one cell type or All neurons (Chi-squared test followed by the Marascuillo procedure was used). (**C**) Normalized confusion matrix for behavioral state decoding using MC5-Crh neurons. (**D**) Response amplitude differences of MC5-Crh neurons across behavioral states. Blue lines are mean responses; error bars are SEM; gray connected circles are individual MC5-Crh neuron responses (one-way repeated-measures ANOVA followed by Tukey-Kramer test). (**E**) Schematic of procedure for decoding behavioral states with the combined temporal response profiles of the PVH cell types using one neuron from each cell type. (**F**) Average confusion matrix with the combined neuronal dynamics of 10 neurons using the procedure in (E). (**G**) Decoding accuracies with the combined neuronal dynamics of 10 neurons from 10 PVH cell types or from 10 dummy cell types that scramble cell type information (fig. S21B). Box plots show the median, interquartile range, and minimum to maximum values of the distributions of decoding accuracies (Wilcoxon rank-sum test). (**H** to **K**) Cell type ensemble response-decoding diagrams for homeostatic and hedonic eating, fear, and ghrelin injection. Diagrams with temporal maximum of consistent-response (proportional to line width) for each cell type and their decoding weights (proportional to arrowhead area) for hunger eating (H), hedonic eating (I), fear retrieval (J), and ghrelin injection (K). Behavioral state decoding is for all 11 behavioral states (also see fig. S24). *P < 0.05, **P < 0.01, ***P < 0.001. Statistics are provided in table S2.

**Fig. 7.**
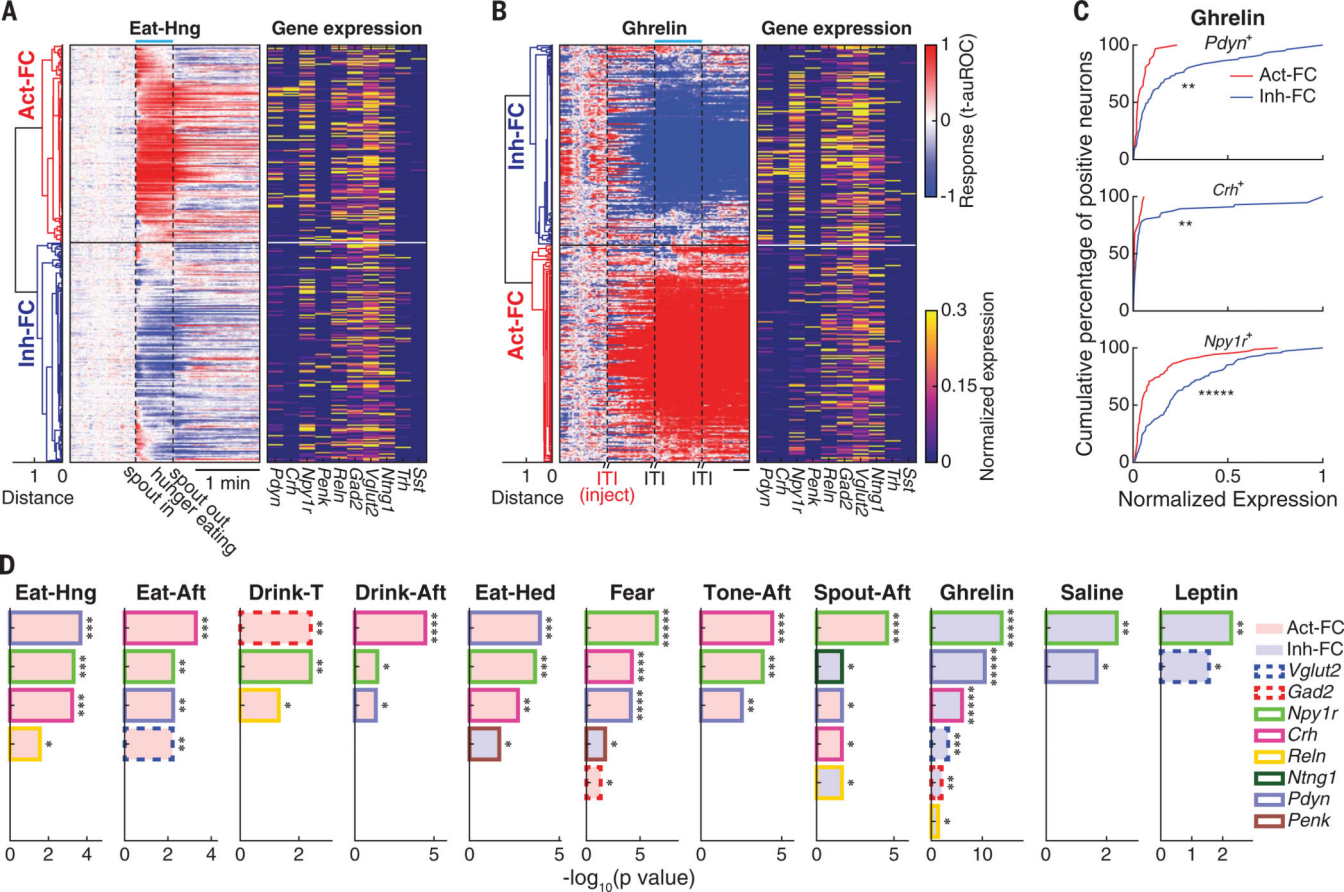
Functional clustering of PVH neurons and differential enrichment of marker genes in 11 behavioral states. (**A** and **B**) Hierarchical clustering of PVH neurons based on their responses while eating in a hunger state (A) and after ghrelin injection (B). Right, gene expression profiles of individual neurons. Blue bar marks the behavioral state. Temporal scale bar, 1 min. (**C**) Comparisons of expression-level distributions of *Pdyn* in *Pdyn*^+^ neurons (top), *Crh* in *Crh*^+^ neurons (middle), and *Npy1r* in *Npy1r*^+^ neurons (bottom) between FCs in (B) after ghrelin injection. (**D**) Enrichment of marker genes in the FCs across 11 behavioral states. Gene enrichments were ranked by –log_10_(*P* value). Gene identity is indicated by bar outline, and the bar fill color indicates the enriched FC. *P < 0.05, **P < 0.01, ***P < 0.001, ****P < 10^−4^, *****P < 10^−5^. Statistics are provided in table S2.

**Fig. 8.**
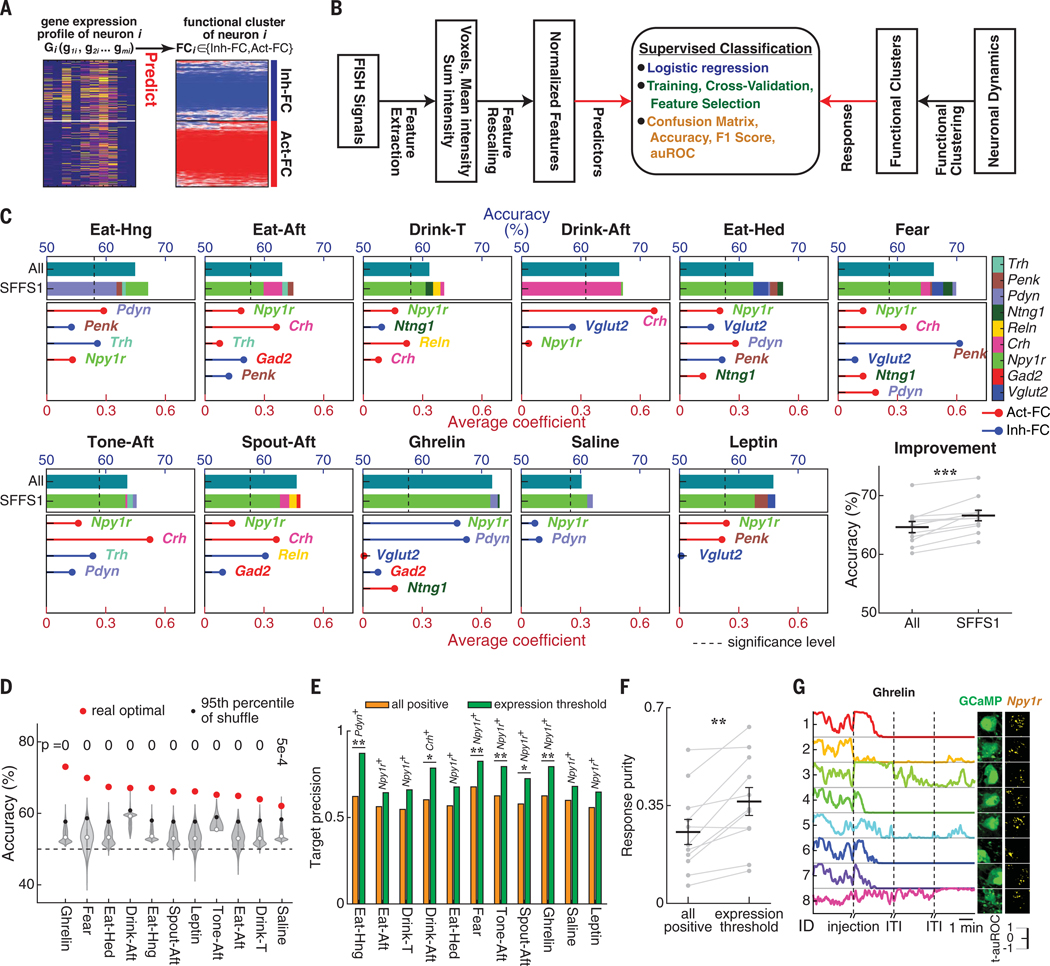
Gene expression predicts FCs in multiple behavioral states. (**A**) Schematic of FC_*i*_ prediction for each neuron solely using its gene expression profile (G_*i*_). (**B**) Supervised learning schematic using logistic regression to predict FCs of PVH neurons by their gene expression profiles (see the materials and methods). (**C**) Predictive accuracies (blue scale) for functional classes using all marker genes (All) and the optimal marker gene set from SFFS1, with stacked bars showing the contribution of individual genes (color legend, right). Bottom subpanels show the average coefficients (red scale) of the SFFS1 genes (see the materials and methods). Stem plot colors indicate to which FC the expression of a gene is positively related. Bottom right panel shows that SFFS1 significantly improved predictive accuracy over using the full marker gene set (All) across all states. (**D**) Predictive accuracies of the optimal gene set for FC prediction ranked for 11 behavioral states (real, red circles) and their corresponding 95th percentile of shuffled accuracies (violin plots). Dashed line indicates 50% accuracy. (**E**) Optimal precision for FC prediction based on expression of a single gene in each behavioral state [first gene in SFFS1 from (C)]. Comparisons between using all neurons expressing that gene (orange) and using the subset of neurons above the optimal expression threshold of that gene (green). (**F**) Comparison of response purities from all neurons expressing the most predictive gene and the subset of neurons above the optimal expression threshold. Each datapoint is response purity in one behavioral state. (**G**) Example responses from predicted Inh-FC neurons after ghrelin injection using *Npy1r*^+^ neurons above the expression threshold for a subset of cells in fig. S32A. Neuron 8 is an example of misclassification by the model (i.e., it was activated despite high *Npy1r* expression). *P < 0.05, **P < 0.01, ***P < 0.001. Statistics are provided in table S2.

We therefore determined the best gene combinations for predicting neuron functional response classes in each behavioral state (Fig. 8A). We used supervised machine learning to predict the FCs of individual PVH neurons solely by their gene expression profiles with a logistic regression classifier (Fig. 8B; see the materials and methods). Three normalized features represented the expression level of each gene (figs. S29 and S30A). This approach does not binarize gene expression by thresholding. Instead, it explicitly incorporates the broad continuous distribution of gene expression levels that we found (Fig. 2H) as the independent variables in the predictive model. Sequential forward feature selection (SFFS) determines the most predictive individual gene for FC classification and sequentially adds the next most predictive gene until predictive accuracy no longer increases. This approach identified optimal gene sets that best predicted the functional response class while minimizing the contribution of redundant gene coexpression information (Fig. 8C and fig. S30, B to D). We compared the predictive power of the molecular profiles for functional response class with the 95th percentile of predictive accuracies after shuffling the relationship between gene expression of individual neurons and their FCs (Fig. 8, C and D, and fig. S30B). The two behavioral states during which neuronal ensemble response could be best predicted by gene expression profiles were during ghrelin-induced hunger [accuracy: 73%, area under the receiver-operating characteristic (auROC): 0.784, *P* = 0) and fear retrieval (accuracy: 69.9%, auROC: 0.745, *P* = 0) states. The lowest predictive accuracy was for saline injection (accuracy: 62.1%, auROC: 0.570, *P* = 0.0005) (Fig. 8D and fig. S30D).

#### Predictive contribution of individual genes

Combinations of genes always had higher predictive power for functional response class than single genes (fig. S30B). In addition, for all behavioral states, the optimal accuracies from the first round of SFFS (SFFS1) with just two to six genes were higher than the accuracies predicted with all genes (Fig. 8C). This showed that a subset of genes provided higher prediction performance than the whole set of genes (41). Next, we quantitatively assessed and ranked the predictive accuracy of single genes for FCs across 11 behavioral states. *Pdyn*, *Npy1r*, or *Crh* showed the highest predictive power in the first round for all behavioral states. *Npy1r* was always in the optimal gene set from SFFS1 in all behavioral states. Moreover, *Npy1r* expression strongly predicted fear-activated neurons. We determined the roles of individual genes within the optimal gene set to improve prediction performance by measuring the average coefficients of those genes from logistic regression (Fig. 8C). For ghrelin-induced hunger, SFFS1 showed an additive relationship for *Npy1r* and *Pdyn* prediction of the inhibited functional response class (Fig. 8C). Both genes have been previously associated with appetite control by the PVH (42). For food consumption during hunger (Eat-Hng), *Pdyn* and *Npy1r* predicted the activated response type, whereas the inhibited response class was predicted by *Trh* and *Penk* (Fig. 8C). For fear retrieval, *Npy1r* and *Crh* best predicted activated neurons and *Penk* predicted fear-inhibited neurons (Fig. 8C).

Multiple rounds of SFFS (mSFFS; see the materials and methods) revealed genes that could compensate for the removal of the most predictive gene from the previous rounds (fig. S30B). This indicated that the most predictive genes could be largely compensated by combinations of additional genes in subsequent rounds. For example, *Pdyn* was most predictive during Eat-Hng, but this predictive accuracy was largely replaced by *Crh* in round 2 and *Npy1r* in round 3 of mSFFS, and we found that *Npy1r* neurons coexpressed *Pdyn* and *Crh* in a hierarchical relationship in pPVH (fig. S30, E and F). Consistent with this, *Npy1r* showed statistically significant predictive power for every behavioral state, but if *Npy1r* was removed from the analysis, its predictive power could be largely replaced by other genes (fig. S30B). Thus, the hierarchical gene expression relationship of these three genes in the pPVH was associated with similar neuron functional responses. Neurons with low or no *Npy1r* expression typically responded oppositely. We also found similar results for the predictive power of marker genes for neuron FCs concatenated across all 11 behavioral states (fig. S31).

#### Gene expression levels for improved FC prediction

Using the quantitative gene expression level to predict functional responses also offers a systematic method for defining the gene expression threshold that targets a functional response class with the best precision for each behavioral state. We identified optimal cut points for the gene expression threshold using Youden’s J statistic with the ROC curve (fig. S32). Prediction precision with a single gene was up to 40% higher, and response purity was significantly improved when the optimal expression threshold was used instead of a lower threshold based on expression over background (Fig. 8, E to G). Thus, neuronal functional identity is not a binary quality of gene expression.

#### Temporal profile of gene → functional response prediction

**Fig. 9.**
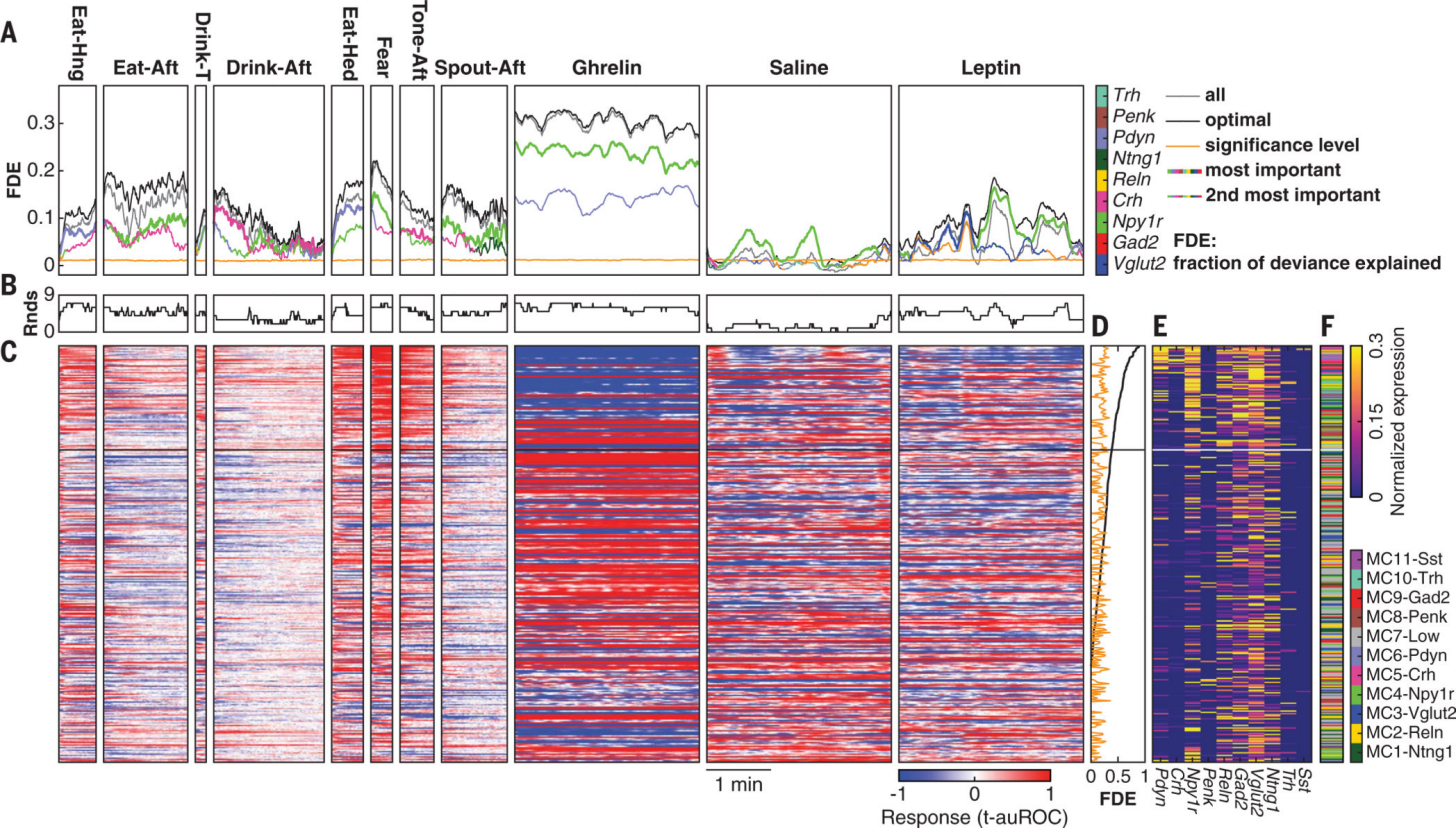
Gene expression profiles of individual PVH neurons predict their temporal responses in multiple behavioral states. (**A**) Prediction performance (fraction of deviance explained, FDE) of neuronal response at each timestamp using expression levels of all marker genes, the optimal gene set, and the two most important genes measured by mSFFS. The optimal gene set was composed of the genes that provided the highest FDE from mSFFS at each timestamp. Significance level is the 95^th^ percentile FDE after shuffling gene expression profiles. (**B**) Number of SFFS rounds with FDE above significance level in each timestamp. (**C**) Responses of PVH neurons ranked by FDE across behavioral states. (**D**) FDE ordered high to low of individual neurons for the entire time series across all behavioral states. (**E** and **F**) Gene expression profiles (E) and molecularly defined cell types (F) of the corresponding neurons in (D). Black or white lines in (C) to (F) indicate the boundary of the most highly predictive quartile.

Next, we investigated how the predictive power of gene expression profiles was related to neuron temporal dynamics. For example, does a gene have similar predictive value throughout a state? For each timestamp (0.4-s interval), we modeled the relationship of gene expression level to neuronal response (Fig. 9, A to C, and fig. S33A). Our results complemented those obtained from prediction of FCs, including (i) higher predictive power of combinatorial expression profile (Fig. 9A), (ii) improvement in prediction performance by mSFFS (Fig. 9A), (iii) redundant information in molecular profiles for predicting temporal response (Fig. 9B), and (iv) that *Npy1r* predictive power was high and statistically significant across all behavioral states (Fig. 9A). However, predictive power depended on cell type–specific neuronal dynamics. During eating and drinking, the predictive power of marker genes increased as consumption progressed. For example, *Pdyn* neuron activity rose more quickly than *Pdyn* predictive accuracy for Eat-Hed and Eat-Hng. In this case, the transient activation at onset of food presentation was not specific within *Pdyn*-expressing neurons but was also widespread in *Pdyn*-negative neurons (fig. S33B). This shows that cell type–specific responses develop progressively in some behaviors. By contrast, fear retrieval showed an immediate cell type–specific response that was best predicted by *Npy1r* and gradually became less well predicted by gene expression (fig. S33B).

In five of 11 behavioral states (Eat-Hng, Drink-Thirst, Eat-Hed, Fear, and Ghrelin), a single gene had the greatest predictive accuracy throughout a behavioral state. However, for six other states, the most predictive gene for the ensemble functional response switched with time. Moreover, using the optimal predictions of neural activity, we ranked neurons by the fraction of deviance explained with our linear regression models along the entire time series across behavioral states (Fig. 9, C and D, fig. S33A). A majority of neurons (65%) showed statistically significant predictive power. Neurons within the top quartile of predictive powers showed significantly higher expression levels of *Vglut2*, *Gad2*, *Npy1r*, *Crh*, *Reln*, *Pdyn*, and *Trh* (fig. S33D). Next, we remapped the corresponding gene expression profiles onto molecularly defined cell types (Fig. 9, E and F). MC4-Npy1r, MC5-Crh (exclusively), and MC6-Pdyn neurons were enriched in the most highly predictive quartile (fig. S33E), consistent with the high predictive accuracy associated with *Npy1r* coexpression.

## DISCUSSION

Neural coding by an ensemble-of-cell-types indicates that many molecularly defined neurons also form functional groupings that work in concert to encode behavioral state. It is unlikely that coding in the pPVH follows the labeled-line model because we could not find such neurons in the ensemble irrespective of molecular identity. Our results validate the use of molecularly defined cell types to reduce the dimensionality of the PVH neuronal ensemble to understand behavioral state coding. Ensemble-of-cell-types coding of behavioral states provides superior efficiency, scalability, and flexibility than labeled-line coding and offers superior robustness (due to redundancy) than cell type–independent, full-ensemble coding. Operationally, grouping by molecularly defined cell types is justified by high consistent-responses and is analogous to the pooling operation in artificial neural networks, which is used to extract invariant information (43–45). This may also be a key function when molecularly defined circuits are used for coding behavioral states, which should be invariant to irrelevant external conditions. Taken together, these cell types and the patterns of activity reported here provide a parts list for systematically examining the neural coding of behavioral state by the PVH. CaRMA imaging offers a quantitative method to evaluate the extent to which other brain regions rely on molecularly defined cell type ensemble coding.

**Fig. 10.**
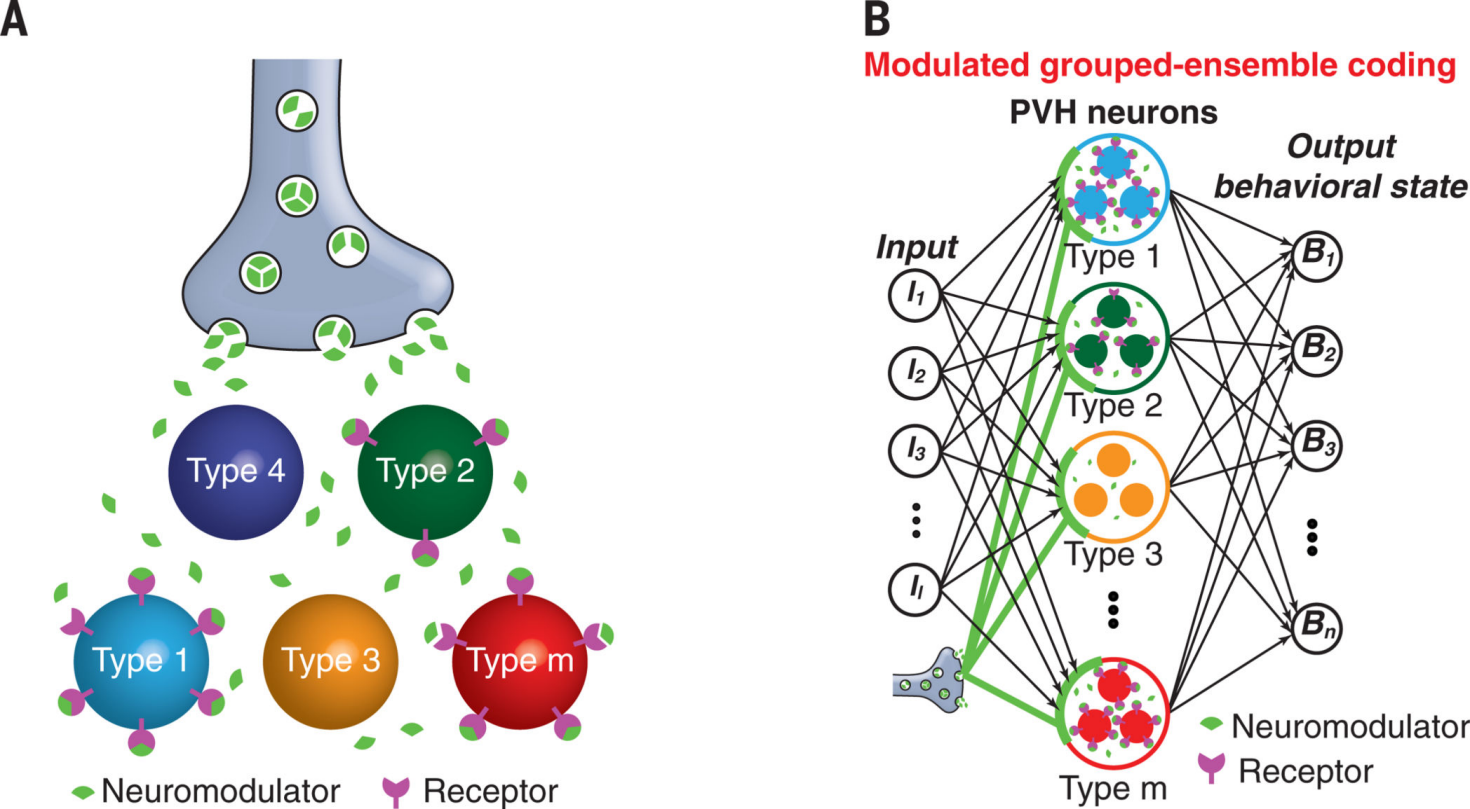
Cooperative regulation of multiple cell types by neuromodulation. (**A**) Diagram illustrating volume diffusion of a neuromodulator to selectively regulate subgroups of cell types expressing its receptor. (**B**) Schematic of modulated grouped-ensemble coding model for multiple behavioral states that includes the role of neuromodulation. Neuromodulator release cooperatively regulates multiple PVH cell types expressing its receptor. This coding configuration highlights the relationship between the hierarchical functional organization of PVH neurons and their molecular hierarchy.

Using CaRMA imaging, we demonstrate an important role for gene expression information to predict both neuron functional response class as well as temporal dynamics within the PVH neural ensemble. High predictive power of *Npy1r* for neuron activity was observed with all analysis methods. However, combinations of other cell type marker genes also showed significant prediction for functional responses. The highest consistent-response cell types, MC5-Crh and MC6-Pdyn, coexpressed *Npy1r*, and cell types that responded oppositely typically lacked *Npy1r*. scRNA-seq analysis revealed hierarchical relationships of gene expression within neuron populations, where the smallest subpopulations are designated as “types” and the largest subdivisions as “classes” that encompass many types (46). We found evidence that this molecular hierarchy extends to functional responses in the neuronal ensemble, in which similarly tuned neuron types form classes that can be well described by a neuromodulator receptor gene that is expressed across multiple molecularly defined cell types (fig. S30, E and F). This is interesting in the context of the neuromodulatory function of *Npy1r* and its endogenous agonist NPY (47). Because *Npy1r* is expressed across multiple cell types and NPY signals through volume transmission (48), this receptor can cooperatively regulate diverse cell types (Fig. 10A). This may enhance the generality of neural output in the presence of noisy neural inputs, which is important to facilitate behavioral state coding but is also a computational role akin to regularization in machine learning. Nevertheless, despite the response similarity between neurons that express *Npy1r*, there are reliable temporal differences that map onto cells with specific gene expression profiles. These temporal responses are important for distinguishing behavioral states and functional response types in the neuronal ensemble. In the analogy of an orchestral ensemble, *Npy1r* is a conductor selectively coordinating the responses of multiple cell types (Fig. 10B) that are like instruments that play together but maintain distinctive contributions to a score.

Marker-gene expression in the PVH can be dynamic (49, 50), but to the extent that this affects our results, it may degrade the predictive accuracy of gene expression level to a functional or behavioral response. Additional genetic markers (51, 52) may improve these predictions by offering increased coverage of all neurons in the ensemble. However, predictive accuracies solely from gene expression information are not expected to be perfect because functional responsiveness is also influenced by plasticity and connectivity mechanisms that are independent of cell type (13, 16, 28). Moreover, our findings also highlight potential limitations of site-specific recombinase (e.g., Cre) mouse lines that use low gene expression thresholds to specify cell type identity (53, 54).

Although we focused here on decoding behavioral states and predicting functional responses solely from molecular information about the underlying cells, we found that if functional information is used to supervise clustering, then molecularly defined cell types can be defined with higher purity (17). This may be useful in the future for better defining combinations of molecular markers and their expression levels that are optimal for targeting specific functional perturbations.

CaRMA imaging provides a solution to the general problem of assessing functional roles for the large number of new molecularly defined cell types that are being discovered routinely using single-cell transcriptomics. Because CaRMA imaging does not rely on genetic modifications, only viral targeting, it is applicable to any organism suitable for in vivo functional imaging. By producing unbiased and systematic datasets that integrate genes, neuron dynamics, and behavior, CaRMA imaging offers a path to discoveries about the organization of brain function at both the molecular level and the systems level.

## Supplementary Material

SM

Movie S1

MDAR Reproducibility Checklist

## Data Availability

The GEO submission ID for the single-cell PVH data is GSE148568. Code for CaRMA imaging analysis pipelines, example data, preprocessed multiplex FISH data, and preprocessed CaRMA imaging data are available at GitHub (56).

## References

[1] Stringer C , Spontaneous behaviors drive multidimensional, brainwide activity. Science 364, 255 (2019). doi: 10.1126/science.aav7893; pmid: 31000656 31000656 PMC6525101

[2] Allen JE, Sutherland TE, Crystal-clear treatment for allergic disease. Science 364, 738–739 (2019). doi: 10.1126/science.aax6175; pmid: 31123126 31123126

[3] Gründemann J , Amygdala ensembles encode behavioral states. Science 364, eaav8736 (2019). doi: 10.1126/science.aav8736; pmid: 31000636 31000636

[4] Chen X , Brain-wide organization of neuronal activity and convergent sensorimotor transformations in larval zebrafish. Neuron 100, 876–890.e5 (2018). doi: 10.1016/j.neuron.2018.09.042; pmid: 30473013 30473013 PMC6543271

[5] Lein ES , Genome-wide atlas of gene expression in the adult mouse brain. Nature 445, 168–176 (2007). doi: 10.1038/nature05453; pmid: 17151600 17151600

[6] Saunders A , Molecular diversity and specializations among the cells of the adult mouse brain. Cell 174, 1015–1030.e16 (2018). doi: 10.1016/j.cell.2018.07.028; pmid: 30096299 30096299 PMC6447408

[7] Zeisel A , Molecular architecture of the mouse nervous system. Cell 174, 999–1014.e22 (2018). doi: 10.1016/j.cell.2018.06.021; pmid: 30096314 30096314 PMC6086934

[8] Tasic B , Shared and distinct transcriptomic cell types across neocortical areas. Nature 563, 72–78 (2018). doi: 10.1038/s41586-018-0654-5; pmid: 30382198 PMC6456269

[9] Adamantidis AR, Zhang F, Aravanis AM, Deisseroth K, de Lecea L, Neural substrates of awakening probed with optogenetic control of hypocretin neurons. Nature 450, 420–424 (2007). doi: 10.1038/nature06310; pmid: 17943086 17943086 PMC6744371

[10] Atasoy D, Sternson SM, Chemogenetic tools for causal cellular and neuronal biology. Physiol. Rev. 98, 391–418 (2018). doi: 10.1152/physrev.00009.2017; pmid: 29351511 29351511 PMC5866359

[11] Magnus CJ , Ultrapotent chemogenetics for research and potential clinical applications. Science 364, eaav5282 (2019). pmid: 30872534 30872534 10.1126/science.aav5282 PMC7252514

[12] Jazayeri M, Afraz A, Navigating the neural space in search of the neural code. Neuron 93, 1003–1014 (2017). doi: 10.1016/j.neuron.2017.02.019; pmid: 28279349 28279349

[13] Hardcastle K, Ganguli S, Giocomo LM, Cell types for our sense of location: Where we are and where we are going. Nat. Neurosci. 20, 1474–1482 (2017). doi: 10.1038/nn.4654; pmid: 29073649 29073649 PMC6175666

[14] Stevens CF, Neuronal diversity: Too many cell types for comfort? Curr. Biol. 8, R708–R710 (1998). doi: 10.1016/S0960-9822(98)70454-3; pmid: 9778523 9778523

[15] Holy TE, “Yes! We’re all individuals!”: Redundancy in neuronal circuits. Nat. Neurosci. 13, 1306–1307 (2010). doi: 10.1038/nn1110-1306; pmid: 20975750 20975750

[16] Fusi S, Miller EK, Rigotti M, Why neurons mix: High dimensionality for higher cognition. Curr. Opin. Neurobiol. 37, 66–74 (2016). doi: 10.1016/j.conb.2016.01.010; pmid: 26851755 26851755

[17] Northcutt AJ , Molecular profiling of single neurons of known identity in two ganglia from the crab Cancer borealis. Proc. Natl. Acad. Sci. U.S.A. 116, 26980–26990 (2019). doi: 10.1073/pnas.1911413116; pmid: 31806754 31806754 PMC6936480

[18] Armstrong WE, “Hypothalamic supraoptic and paraventricular nuclei,” in The Rat Nervous System, Paxinos G, Ed. (Elsevier, 2004), pp. 369–388.

[19] Romanov RA , Molecular interrogation of hypothalamic organization reveals distinct dopamine neuronal subtypes. Nat. Neurosci. 20, 176–188 (2017). doi: 10.1038/nn.4462; pmid: 27991900 27991900 PMC7615022

[20] Swanson LW, Sawchenko PE, Hypothalamic integration: Organization of the paraventricular and supraoptic nuclei. Annu. Rev. Neurosci. 6, 269–324 (1983). doi: 10.1146/annurev.ne.06.030183.001413; pmid: 6132586 6132586

[21] Li MM , The paraventricular hypothalamus regulates satiety and prevents obesity via two genetically distinct circuits. Neuron 102, 653–667.e6 (2019). doi: 10.1016/j.neuron.2019.02.028; pmid: 30879785 30879785 PMC6508999

[22] Krashes MJ , An excitatory paraventricular nucleus to AgRP neuron circuit that drives hunger. Nature 507, 238–242 (2014). doi: 10.1038/nature12956; pmid: 24487620 24487620 PMC3955843

[23] Füzesi T, Daviu N, Wamsteeker Cusulin JI, Bonin RP, Bains JS, Hypothalamic CRH neurons orchestrate complex behaviours after stress. Nat. Commun. 7, 11937 (2016). doi: 10.1038/ncomms11937; pmid: 27306314 PMC4912635

[24] Saper CB, Lowell BB, The hypothalamus. Curr. Biol. 24, R1111–R1116 (2014). doi: 10.1016/j.cub.2014.10.023; pmid: 25465326 25465326

[25] Ishii KK , A labeled-line neural circuit for pheromone-mediated sexual behaviors in mice. Neuron 95, 123–137.e8 (2017). doi: 10.1016/j.neuron.2017.05.038; pmid: 28648498 28648498

[26] Graebner AK, Iyer M, Carter ME, Understanding how discrete populations of hypothalamic neurons orchestrate complicated behavioral states. Front. Syst. Neurosci. 9, 111 (2015). doi: 10.3389/fnsys.2015.00111; pmid: 26300745 PMC4523943

[27] Sternson SM, Hypothalamic survival circuits: Blueprints for purposive behaviors. Neuron 77, 810–824 (2013). doi: 10.1016/j.neuron.2013.02.018; pmid: 23473313 23473313 PMC4306350

[28] Hebb DO, The Organization of Behavior (Erlbaum, 1949/2002).

[29] Moffitt JR , Molecular, spatial, and functional single-cell profiling of the hypothalamic preoptic region. Science 362, eaau5324 (2018). doi: 10.1126/science.aau5324; pmid: 30385464 30385464 PMC6482113

[30] Lee D, Kume M, Holy TE, Sensory coding mechanisms revealed by optical tagging of physiologically defined neuronal types. Science 366, 1384–1389 (2019). doi: 10.1126/science.aax8055; pmid: 31831669 31831669 PMC7591936

[31] Lovett-Barron M , Multiple convergent hypothalamus-brainstem circuits drive defensive behavior. Nat. Neurosci. 23, 959–967 (2020). doi: 10.1038/s41593-020-0655-1; pmid: 32572237 32572237 PMC7687349

[32] Lovett-Barron M , Ancestral circuits for the coordinated modulation of brain state. Cell 171, 1411–1423.e17 (2017). doi: 10.1016/j.cell.2017.10.021; pmid: 29103613 29103613 PMC5725395

[33] Kerlin AM, Andermann ML, Berezovskii VK, Reid RC, Broadly tuned response properties of diverse inhibitory neuron subtypes in mouse visual cortex. Neuron 67, 858–871 (2010). doi: 10.1016/j.neuron.2010.08.002; pmid: 20826316 20826316 PMC3327881

[34] Barnett LM, Hughes TE, Drobizhev M, Deciphering the molecular mechanism responsible for GCaMP6m’s Ca^2+^-dependent change in fluorescence. PLOS ONE 12, e0170934 (2017). doi: 10.1371/journal.pone.0170934; pmid: 28182677 28182677 PMC5300113

[35] Li C , Defined paraventricular hypothalamic populations exhibit differential responses to food contingent on caloric state. Cell Metab. 29, 681–694.e5 (2019). doi: 10.1016/j.cmet.2018.10.016; pmid: 30472090 30472090 PMC6402975

[36] Kim J , Rapid, biphasic CRF neuronal responses encode positive and negative valence. Nat. Neurosci. 22, 576–585 (2019). doi: 10.1038/s41593-019-0342-2; pmid: 30833699 30833699 PMC6668342

[37] Mandelblat-Cerf Y , Bidirectional anticipation of future osmotic challenges by vasopressin neurons. Neuron 93, 57–65 (2017). doi: 10.1016/j.neuron.2016.11.021; pmid: 27989461 27989461 PMC5215952

[38] Cohen JY, Haesler S, Vong L, Lowell BB, Uchida N, Neuron-type-specific signals for reward and punishment in the ventral tegmental area. Nature 482, 85–88 (2012). doi: 10.1038/nature10754; pmid: 22258508 22258508 PMC3271183

[39] Betley JN , Neurons for hunger and thirst transmit a negative-valence teaching signal. Nature 521, 180–185 (2015). doi: 10.1038/nature14416; pmid: 25915020 25915020 PMC4567040

[40] Li Y , Neuronal representation of social information in the medial amygdala of awake behaving mice. Cell 171, 1176–1190. e17 (2017). doi: 10.1016/j.cell.2017.10.015; pmid: 29107332 29107332 PMC5731476

[41] Trunk GV, A problem of dimensionality: A simple example. IEEE Trans. Pattern Anal. Mach. Intell. 1, 306–307 (1979). doi: 10.1109/TPAMI.1979.4766926; pmid: 21868861 21868861

[42] Kask A, Rägo L, Harro J, Evidence for involvement of neuropeptide Y receptors in the regulation of food intake: Studies with Y_1_-selective antagonist BIBP3226. Br. J. Pharmacol. 124, 1507–1515 (1998). do 10.1038/sj.bjp.0701969; pmid: 9723965 9723965 PMC1565528

[43] Ranzato M, Huang FJ, Boureau Y, LeCun Y, “Unsupervised learning of invariant feature hierarchies with applications to object recognition,” in IEEE Conference on Computer Vision and Pattern Recognition (IEEE, 2007), pp. 1–8; 10.1109/CVPR.2007.383157.

[44] Hubel DH, Wiesel TN, Receptive fields, binocular interaction and functional architecture in the cat’s visual cortex. J. Physiol. 160, 106–154 (1962). doi: 10.1113/jphysiol.1962.sp006837; pmid: 14449617 14449617 PMC1359523

[45] LeCun Y, Kanter I, Solla SA, “Second order properties of error surfaces: Learning time and generalization,” in Advances in Neural Information Processing Systems 3, Lippmann RP, Moody JE, Touretzky DS, Eds. (NIPS, 1990), pp. 396–404.

[46] Zeng H, Sanes JR, Neuronal cell-type classification: Challenges, opportunities and the path forward. Nat. Rev. Neurosci. 18, 530–546 (2017). doi: 10.1038/nrn.2017.85; pmid: 28775344 28775344

[47] Herzog H, 30 Years of NPY research. Neuropeptides 46, 251 (2012). doi: 10.1016/j.npep.2012.10.002; pmid: 23141043

[48] Fetissov SO, Kopp J, Hökfelt T, Distribution of NPY receptors in the hypothalamus. Neuropeptides 38, 175–188 (2004). doi: 10.1016/j.npep.2004.05.009; pmid: 15337370 15337370

[49] Romanov RA, Alpár A, Hökfelt T, Harkany T, Molecular diversity of corticotropin-releasing hormone mRNA-containing neurons in the hypothalamus. J. Endocrinol. 232, R161–R172 (2017). doi: 10.1530/JOE-16-0256; pmid: 28057867 28057867

[50] Watts AG, Swanson LW, Diurnal variations in the content of preprocorticotropin-releasing hormone messenger ribonucleic acids in the hypothalamic paraventricular nucleus of rats of both sexes as measured by in situ hybridization. Endocrinology 125, 1734–1738 (1989). doi: 10.1210/endo-125-3-1734; pmid: 2788078 2788078

[51] Chen KH, Boettiger AN, Moffitt JR, Wang S, Zhuang X, RNA imaging. Spatially resolved, highly multiplexed RNA profiling in single cells. Science 348, aaa6090 (2015). doi: 10.1126/science.aaa6090; pmid: 25858977 25858977 PMC4662681

[52] Shah S, Lubeck E, Zhou W, Cai L, In situ transcription profiling of single cells reveals spatial organization of cells in the mouse hippocampus. Neuron 92, 342–357 (2016). doi: 10.1016/j.neuron.2016.10.001; pmid: 27764670 27764670 PMC5087994

[53] Harris JA , Anatomical characterization of Cre driver mice for neural circuit mapping and manipulation. Front. Neural Circuits 8, 76 (2014). doi: 10.3389/fncir.2014.00076; pmid: 25071457 25071457 PMC4091307

[54] Lee KY , Lessons on conditional gene targeting in mouse adipose tissue. Diabetes 62, 864–874 (2013). doi: 10.2337/db12-1089; pmid: 23321074 23321074 PMC3581196

[55] Biag J , Cyto- and chemoarchitecture of the hypothalamic paraventricular nucleus in the C57BL/6J male mouse: A study of immunostaining and multiple fluorescent tract tracing. J. Comp. Neurol. 520, 6–33 (2012). doi: 10.1002/cne.22698; pmid: 21674499 21674499 PMC4104804

[56] Xu S, Yang H, Menon V, Lemire AL, Wang L, Henry FE, Turaga SC, Sternson SM, Code for CaRMA imaging analysis pipelines, example data, preprocessed multiplex FISH data, and preprocessed CaRMA imaging data for: Behavioral state coding by molecularly defined paraventricular hypothalamic cell type ensembles, GitHub (2020); https://github.com/sternson-lab/CaRMA-imaging. 10.1126/science.abb2494 PMC11938375 33060330

