# Supplementary material for: Behavioral state coding by molecularly defined paraventricular hypothalamic cell type ensembles
Source: Science. Author manuscript; Available in PMC 2025 Mar 26. (PMC11938375; doi:10.1126/science.abb2494)
Supplement: SM [file NIHMS1900648-supplement-SM.pdf]

science.sciencemag.org/content/370/6514/eabb2494/suppl/DC1

Supplementary Materials for

Behavioral state coding by molecularly defined paraventricular hypothalamic cell type ensembles

Shengjin Xu*, Hui Yang, Vilas Menon, Andrew L. Lemire, Lihua Wang, Fredrick E. Henry, Srinivas C. Turaga, Scott M. Sternson*

*Corresponding author. Email: xus@janelia.hhmi.org (S.X.); sternsons@janelia.hhmi.org (S.M.S.)

Published 16 October 2020, *Science* **370**, eabb2494 (2020)
DOI: 10.1126/science.abb2494

This PDF file includes:

Materials and Methods
Figs. S1 to S33
Tables S1 and S2
Caption for Movie S1
References

Other Supplementary Material for this manuscript includes the following:
(available at science.sciencemag.org/content/370/6514/eabb2494/suppl/DC1)

MDAR Reproducibility Checklist
Movie S1

Materials and Methods

All experimental protocols were conducted according to U.S. National Institutes of Health guidelines for animal research and approved by the Institutional Animal Care and Use Committee at Janelia Research Campus.

Mice

Mice were housed on a 06:00-18:00h light cycle with water and mouse chow ad libitum (PicoLab Rodent Diet 20, 5053 tablet, TestDiet) unless otherwise noted. Ten male *Agrp-IRES-Cre* (Jackson Labs Stock 012899, *Agrp*$^{tm1(cre)Lowl}$/J) crossed with *Ai9* (Jackson Labs Stock 007909, B6.Cg-*Gt(ROSA)26Sor*$^{tm9(CAG\text{-}tdTomato)Hze}$/J) mice (> 7 weeks) were used for scRNA-Seq. Two adult male (> 8 weeks) *Sim1-Cre* (Jackson Labs Stock 006395, Tg(Sim1-cre)1Lowl/J) mice were used for the FISH-only experiment. Three adult male (> 8 weeks) *Sim1-Cre* (Jackson Labs Stock 006395, Tg(Sim1-cre)1Lowl/J) mice were used for CaRMA imaging. There was no randomization or blinding of experimental treatments. The sample size was determined based on conventions in the field.

Single cell RNA sequencing

Single cell suspensions were prepared as described previously (*57*). Briefly, brain sections were cut and treated with protease digestion. We used the axon projection field of AGRP neurons (from Agrp-IRES-Cre × Ai9 crosses) to identify the boundary of the PVH. The PVH tissue was microdissected, dissociated, and individual cells were manually purified. Each single cell was isolated into 3 µl mild lysis buffer (nuclease-free water with 0.2% Triton and 0.1 U/µl RNase inhibitor). Cells in lysis buffer were flash frozen on dry ice and stored at -80 °C. cDNA was prepared from sorted single cells using Smart-SCRB chemistry as described previously (*58*) with minor modifications. Reverse transcription by using barcoded RT primer, PCR amplification and purification, tagmentation, and library quantification were performed as described previously (*58*). Libraries were obtained by modified Nextera XT (Illumina) with P5NEXTPT5 and i7 primers and sequenced on a NextSeq 550 flowcell with 25 bases in read 1, 8 bases in the i7 index read, and 50 bases in read 2. PhiX control library (Illumina) was spiked in at a final concentration of 15% to improve color balance in read 1.

Analysis of scRNA-Seq data

To identify clusters from single-cell RNA-seq data, we used an iterative gene-clustering based approach, modified slightly from previous work in (*8, 59*). This approach consisted of the following steps:

1. Starting from the raw UMI values, identify genes with variance greater than technical noise, the latter based on ERCC spike-in control variance.
2. Use Weighted Gene Co-expression Network Analysis (WGCNA) (*60*) to cluster variable genes into modules.
3. Cluster cells using Euclidean distance on the module eigengenes (as the reduced dimension) with a hierarchical approach with Ward's method. Define the set of clusters using a dynamic tree cutting method.

4. For each pair of cell clusters in 3), identify differentially expressed genes, and sum the total log(p-values) of differential expression. If the sum is less than a specified threshold, merge these clusters together. Repeat until no more cluster pairs (or merged cluster pairs) can be merged.
5. Repeat steps 1-4 on each cluster until no further sub-clusters are identified. This may be due to a) no significant genes showing more variation than technical noise, b) no coherent WGCNA modules or c) no distinct clusters with differentially expressed genes.

This entire process was run on 100 bootstrapped subsets of 80% of the cells, thus providing a cell-cell co-clustering matrix. This co-clustering matrix allows for the determination of an average within- and between-cluster similarity, to further characterize the distinctness of clusters.

Within the data, a small subset of clusters was found to be non-neuronal, based on known glial markers (*Aqp4* for astrocytes, *Olig2* and *Opalin* for oligodendrocytes, *Pdgfra* for oligodendrocyte precursors, and *Ctss* and *Aif1* for microglia). These clusters were not included in the downstream analysis.

We identified differentially expressed genes across clusters using the DE-Seq2 package (*61*). To refine these genes into the set used for multiplexed FISH, we selected a subset that showed good on-off expression between individual clusters. "On" expression was defined as being detected in >40% of cells within a cluster, and "off" expression was defined as detection in <5% of cells within a cluster.

<u>Multiplex FISH of PVH neurons in FISH-only tissue</u>

To examine the spatial organization of gene expression profiles of PVH neurons along the anterior to posterior axis of the entire PVH, we used 2 mice of the same strain as in CaRMA imaging (*Sim1-Cre*) but not subjected to behavioral or surgical perturbations. We collected three coronal cryosections (14 µm) from aPVH, mPVH and pPVH (separated by 280 µm sequentially) in each mouse. We then performed 12-plex FISH experiments using RNAscope® Multiplex Fluorescent Assay on these 6 sections.

We modified the RNAscope® protocol to enable image registration across 4 rounds of 3-plex FISH. Specifically, prior to the RNAscope® assay, we stained the sections with DAPI, applied 4% paraformaldehyde post-staining, and then digested genomic DNA with RNase-free DNase I (Qiagen Cat # 79254, 2 K units/µl, using 75-100 µl to cover the entire section) for 4 hours at 37°C, exchanging for fresh DNase I after the first 2 hours. DNase I digestion at this stage (after DAPI initial staining, paraformaldehyde fixing, but before FISH) allows to generate consistent DAPI staining patterns throughout all rounds of imaging. We then pre-treated sections according to RNAscope® protocol but extended protease IV digestion (105 min) and inactivated residual protease IV activity by incubating the sections with 1 mM phenylmethylsulfonyl fluoride (PMSF, Millipore Sigma-Aldrich SKU 93482) at room temperature (30 min). We performed 3-plex FISH to detect 3 marker-genes according to the manufacturer's instructions, imaged DAPI (405 nm laser) and FISH signals (488, 561, and 633 nm lasers). We then removed FISH oligonucleotides by 4-hr DNase I digestion as described above (fig. S2) and conducted the next round of 3-plex FISH for another 3 marker-genes. Twelve marker-genes were detected by 4 rounds of 3-plexed FISH. Before probe hybridization in each round, we incubated the sections with 1 mM $CuSO_4$ in 1× SSC at room temperature for 30 minutes

to reduce lipofuscin autofluorescence and then with 10 mg/mL yeast tRNA (Thermo Fisher Scientific Cat # AM7119) at 40°C (30 min) to reduce non-specific probe and oligonucleotide binding.

To draw ROIs of all neurons by cell segmentation and register these ROIs to FISH images, we performed a round of DAPI and Syto staining after FISH (fig. S3I). We found Syto 81 (Thermo Fisher Cat. No. S11360) could stain cell boundaries (thus enabling segmentation) and was compatible with DAPI staining (it did not alter the DAPI staining pattern, which enabled registration). Syto staining was followed by briefly photo-bleaching to improve image contrast and showed, for each nucleus, a bright spot surrounded by a ring of Syto fluorescence in most neurons (fig. S3A). This pattern was useful for cell segmentation (see *3D segmentation*). Syto 81 was diluted 1:400 in molecular grade water, applied to the section for 15 minutes, and washed twice. The PVH region on each section was photo-bleached on the confocal microscope by 561 nm laser illumination throughout the entire section thickness until the "nucleus-bright-spot-within-a-ring" pattern became apparent and had sufficient contrast with the background. Then we acquired DAPI and Syto images in Round 5 after FISH as shown in fig. S3A.

All images were acquired on a Zeiss LSM880 NLO upright confocal microscope with a 20× water-dipping objective (1.0 NA) using PBS as immersion media. DAPI staining from these 5 rounds of imaging were associated with some residual DAPI-bound DNA and DAPI bound to RNA ("DAPI residual" signal after DNase I digestion and protease treatment, DAPI_R in fig. S4A) and used for registering marker-gene expression of the same neurons across rounds (fig. S3I).

<u>Imaging processing of multiplex FISH in FISH-only tissue</u>

3D segmentation

To generate gene expression profiles of individual neurons, we first performed semi-automatic 3D cell segmentation using Syto signal in Round 5 (fig. S3, A to H; step 1 in fig. S3I)). We found a specific pattern of Syto staining after DNase I digestion that there was only one nucleus-bright-spot in most neurons. We used the nucleus-bright-spots as seeds for 3D watershed segmentation. We obtained the maximum intensity projection (MIP) 2D image of 3D Syto image and their corresponding z-indices using Matlab max function (fig. S3A, [img2D, indZ] = max(img3D, [], 3), where img3D is the matrix of 3D Syto image, img2D is the matrix of 2D Syto MIP image and indZ is the z-indices of the img2D in img3D. The interactive machine learning image segmentation software (ilastik) was used to generate 2D probability maps of background, cell bodies and nucleus bright spots from 2D Syto MIP image (fig. S3B). The 2D probability map was then binarized, visually inspected and manually corrected (fig. S3C). In the manual correction, we ensured that each neuron had only one nucleus bright spot. Each correct 2D nucleus-bright-spot was mapped to 3D at z-depth, where z-depth is the median of indZs within 2D nucleus-bright-spot pixels. The 3D nucleus-bright-spots were the seeds for 3D segmentation (3D_seeds). We duplicated the masks of 2D cell bodies (including red and green labels in fig. S3C) to all depth to generate 3D masks of cell bodies (3D_cell_mask). Binarized 3D Syto fluorescence (3D_Syto_b) was generated using local adaptive Otsu's threshold. The 3D cell body foreground for segmentation was the intersection of 3D_cell_mask and 3D_Syto_b. This 3D cell body foreground was segmented into individual cells with the 3D_seeds using 3D watershed plugin in Fiji (*62*) (fig. S3F). We

randomly chose 1200 ROIs (100 ROIs in each location, 2 locations (left and right) from each of the 3 PVH regions (aPVH, mPVH and pPVH) in 2 mice, visually inspected the segmentation results, and found 90% ROIs were correct (4% under-segmentation and 6% over-segmentation).

Multi-round registration of brain slices using DAPI fluorescence for multiplex FISH

After 3D cell segmentation, we generated gene expression profiles of individual neurons in the FISH-only tissue (fig. S3I). Matlab scripts based on ANTs toolkit (*63*) (http://picsl.upenn.edu/software/ants/) running in Janelia Computer Cluster were used for 3D image registrations and 3D image transformations. We used the image stack of DAPI residual from Round 3 as the reference image stack. We aligned image stacks of DAPI residual from other rounds with the reference stack and obtained their transformations (*Transformation_Ri*, $i = \{1, 2, 4, 5\}$; step 2 in fig. S3I; and fig.S4A). Images of FISH signals probed in other rounds and neuron ID (3D ROIs) were transformed to aligned images using their corresponding *Transformation_Ri* (step 3 in fig. S3I). Finally, we merged the aligned neuron ID and aligned FISH signals to generate an image stack of gene expression profiles of individual neurons (step 4 in fig. S3I).

Multi-round FISH signal extraction

Background was estimated for each FISH image using the 99th percentiles of pixel fluorescence intensity signals within background regions (i.e., fluorescence signals without cellular shape by visual inspection). The background was subtracted from each FISH image. mRNA transcript signals were identified using Otsu’s threshold. Nonspecific fluorescence signals were detected as colocalization pixels of the 3 mRNA transcripts from the same FISH round and removed from the FISH images. We found that the *Avp* probe from RNAScope could hybridize with *Oxt* mRNA. To remove the false positive of *Avp* signal, we subtracted *Oxt* images from their *Avp* images. Three features (voxels, mean intensity and sum intensity, where sum intensity = voxels * mean intensity) of each mRNA signal were extracted from individual neurons in the clean FISH images. To reduce the false positive of *Avp* detection, we further corrected the 3 features of *Avp* signal using a conservative procedure: if *Avp_S*/*Oxt_S* > 10, keep the 3 feature values of *Avp*; otherwise set the 3 feature values of *Avp* to zeros, where *Avp_S* and *Oxt_S* are the sum intensities of *Avp* and *Oxt* respectively. To reduce the batch effect of individual animals, these 3 features of each mRNA transcript signal were further normalized to [0 1] by (*fval_i* – *fval_n_min*) / (*fval_n_max* – *fval_n_min*), where *fval_i* is the feature value from Neuron *i*, *fval_n_min* and *fval_n_max* are the minimum and maximum feature values among neurons from the same Animal *n* as Neuron *i*.

Classification of molecular clusters for multiplexed FISH data

Unsupervised hierarchical clustering algorithm was used to classify these neurons using their sum intensities of all genes (z-score normalized values). The optimal cluster number was 13, chosen with Calinski-Harabasz index (Fig. 2D). tSNE visualization of these molecularly defined cell types (Fig. 2F) were based on their sum intensities of all genes (normalized values) using tsne function in Matlab (Distance = correlation, Standardize = true, Perplexity = 40). To generate the spatial organization map of these 13 molecularly defined cell types in the 3 PVH regions (Fig. 2G, and fig. S6, A and B), for each PVH region, we horizontally flipped the images from the left side and manually aligned them with the images from the right side using TrakEM2 with rigid transformation. The 3rd ventricle contour and cell density were used as guidance in the

manually alignment of images from different mice. We imported the transformation matrices from TrakEM2 into Matlab, applied the transformations to individual neurons and symbolized the neurons with their cell type markers.

Threshold for "positive" gene expression

We considered a neuron as being positive for expression of a gene *xxx* (xxx^+) using a criterion $Voxel\_xxx > 4$, where $Voxel\_xxx$ is the number of voxels with FISH signal of gene *xxx* in that neuron.

Correspondence between scRNA-seq and multiplex FISH

To quantify the correspondence between gene expression profiles measured with scRNA-seq and multiplexed FISH, we identified Seq-neurons that significantly correlated with FISH-clusters and FISH-neurons that significantly correlated with Seq-clusters. To identify the significant Seq-neurons, we first computed the pairwise Pearson's correlation coefficient between the average expression profiles (z-score normalized values) of each FISH-cluster and the expression profiles (z-score $\log_{10}$(TPM+1)) of Seq-neurons. We then estimated the significant correlation coefficient baseline by calculating the 90th percentile of correlation coefficients from the bootstrap replicates (shuffle the gene labels associated with the average expression profiles of FISH-clusters 1000 times). Next, we identified the Seq-neurons with correlation coefficients greater than the significant baseline ($r > 0.47$) and statistically significant p values ($p < 0.05$). These significantly correlated Seq-neurons were ordered by their correlation coefficients along the FISH-clusters (fig. S6C) and exclusively filtered by their lowest p values and highest correlation coefficients (fig. S6D). Similarly, FISH-neurons significantly correlated with Seq-clusters were identified ($r > 0.41$ and $p < 0.05$; fig. S6, E and F). We further evaluated the anatomic distributions of the significantly correlated FISH-neurons for each Seq-cluster and all Seq-clusters (fig. S6G).

Viral injections and GRIN lens implantation

Viral injections and implantations of GRIN lens (Part ID: GLP-0584; 0.5 mm in diameter, and 8.2 mm in length; Inscopix) were performed as described previously (*39*). Briefly, AAV2/1-Syn-FLEX-GCaMP6m (1.2×10^{13} GC/ml, Janelia) viral vector was diluted (1:5, final 2.4×10^{12} GC/ml) with PBS and injected at two depths in the left PVH (bregma: -0.75 mm; midline: 0.3 mm; dorsal surface: -4.55 mm and -4.75 mm (100 nl/site)). After 3-4 weeks for transgene expression, a GRIN lens was implanted to target the GCaMP-expressing PVH neurons (bregma: -0.8 mm; midline: 0.3 mm). The target depth (dorsal surface: -4.80 mm to -4.65 mm) was determined by observing fluorescent signal through a miniature microscope (nVista HD v2, Inscopix). Then, the GRIN lens and a head bar were fixed with black dental cement (Lang Dental Manufacturing). A layer of parafilm covered the top end of the lens. A silicone adhesive (Kwik-Sil; World Precision Instruments) was applied above the parafilm to protect the lens.

Two-photon calcium imaging in head-fixed behaving mice

After one-week recovery from the GRIN lens implantation surgery, the mice started food-restriction (3 g of food pellets per day, *ad libitum* access to water) with daily health monitoring and body weight assessments. While under food-restriction, the mice were habituated to head-fixation and eating liquid food (40% paste-like liquid food by

warming liquid diet with distilled water; PMI Micro-stabilized rodent liquid diet LD101) through a lick-spout on a treadmill (Janelia). The habituation training continued until the head-fixed animals could stay on the treadmill without frequent struggling movement for more than 1 hour. During the habituation training period, the neuronal fluorescence intensity was monitored (once a day, only at the end of habituation) with the Inscopix miniature microscope to examine the recovery and stability of the brain tissue under GRIN lens. Once the habituation criterion was reached and the fluorescent structure under the GRIN lens stayed the same across 3 days, mice were moved to a two-photon microscopy rig to acclimatize (2 days, 1 hour/day) to the two-photon imaging environment. Then, a set of calcium imaging experiments involving eating during hunger, drinking water during thirst, hedonic eating (*ad lib* fed, Ensure palatable food), fear retrieval, and hormone-induced hunger (ghrelin injection) or energy surfeit (leptin injection) were sequentially performed over 10 days (fig. S7, see Behaviors).

Calcium imaging was performed using a custom-built two-photon microscope (Modular In Vivo Multiphoton Microscope, MIMMS, Janelia) controlled by ScanImage (Vidrio Technologies). The two-photon microscope was equipped with some customized components for deep-brain imaging with a graded-index (GRIN) lens, which included a fast resonant scanner module (12 kHz, Cambridge Technology), an assistant epifluorescence imaging module (Thorlabs) for GRIN lens alignment, a high dispersion compensation module (Janelia) and a FastZ scanner module (P-725 PIFOC Objective Scanner, PI, 400 µm travel range). The light source was a Chameleon Ultra femtosecond pulsed laser (Coherent) running at 920 nm. The objective was a 10× air lens (CFI Plan Apo Lambda 10×, Nikon, 0.45 NA, 4 mm working distance). The power used was 90-100 mW measured after the objective lens and before the GRIN lens.

To facilitate aligning the GRIN lens with the objective lens, the head-bar holder and the treadmill were fixed on αβ axis goniometers (OptoSigma). Before each calcium imaging experiment, the parafilm and silicone adhesive above the GRIN lens were removed. The GRIN lens top surface was cleaned with lens paper. Then, the GRIN lens was aligned with the objective lens by adjusting the goniometers. The criterion for a good alignment was when the entire top circular edge of the GRIN lens simultaneously became sharp by epifluorescence imaging. After the alignment, the objective position focusing on the GRIN lens top surface was set as the reference zero for the later aberration corrections. In each imaging experiment, besides the functional trials with fast volumetric imaging (FastZ image: 256 × 256 pixels, 8 imaging planes within 400 µm objective travel range, 7.75 Hz volume rate, sawtooth scanning mode) of neuronal activity in the defined behaviors, there were several meta trials with slow z-stack imaging (ZStack image: 512 × 512 pixels, 5 µm step size, 600 µm objective travel range covering the travel range in functional trials) of some fluorescent fiducial structures, such as the distinct neurites, before and after the functional trials. These meta trials were used to assess the motion of the brain tissue, facilitate longitudinally tracking the same neurons along all imaging experiments and the *ex vivo* → *in vivo* image registration. After each imaging experiment, a new layer of parafilm and silicone adhesive were used to protect the GRIN lens.

Stimulus delivery and behavior control and log system

A custom-built behavior-control-log-system (BCLS) was used to control all behavior procedures in the imaging experiments (fig. S7), record the behavior events and synchronize behavior events, two-photon imaging and behavior video acquisitions. The system included 3 Arduino boards (2 Arduino Mega 2560 and 1 Arduino Due). One Mega 2560 generated TTL signals to trigger two-photon imaging acquisition and frame acquisitions of behavior videos in two cameras (front and side, Basler acA640-120um). The other Mega 2560 controlled the behavior logic procedures. The Due board synchronized (20 µs precision) all behavior events and the onsets of frame acquisitions from two-photon imaging and behavior videos and sent these synchronized events to a computer. The computer then stored the synchronized events. All the programs running on the Arduino boards were coded with Arduino IDE. The program running in the computer to communicate with Arduino boards and log synchronized events was coded with C++ (Visual studio community, Microsoft). Behavior video acquisition program was developed based on iSpy (https://www.ispyconnect.com) with C# (Visual studio community, Microsoft). In experiments shown in fig. S7, A, B, C and E, a linear step motor (Zaber Technologies Inc.) was used to extend and retract the lick spout, which was controlled and recorded by the BCLS. Licking was detected with a capacitive sensor and recorded by the BCLS.

Food and water delivery

Liquid food, water and Ensure were delivered with a syringe pump (NE-500, New Era Pump Systems, Inc), which was closed-loop controlled (3 µl every 3 licks) and recorded by the BCLS.

Behavioral experiments

Day1: Eating during hunger (food restriction)

For the Hunger-Eat experiment (fig. S7A), the lick spout was extended near the mouse's mouth 1-min after the trial onset. Mice immediately started eating the paste-like liquid food. The lick spout was retracted 35-s after the spout extension. The trial stopped 80-s after spout retraction. After the Hunger-Eat experiment (7-9 trials), animals were *ad libitum* fed until all experiments were completed.

Day 2: Drinking water during thirst (16-h water deprivation)

For the Thirst-Drink experiment (fig. S7B), mice were moderately water deprived (16 hours). This was because in 24-h water deprived mice we found that rehydration by drinking could dramatically change the imaging planes in PVH (surrounding the 3rd ventricle), likely due to brain expansion following changes in blood osmolarity. The spout was extended 1-min after the trial onset. Mice immediately started drinking water. The lick spout was retracted 10-s after the spout extension. The trial stopped 105-s after spout retraction. After the session (5-7 trials) mice were given *ad libitum* water.

Day 3-5: Hedonic eating and fear retrieval

Mice were habituated to eat palatable Ensure in the imaging rig (fig. S7C). During the habituation training, the spout was extended 30-s after the trial onset. Mice immediately started licking the palatable Ensure. The lick spout was retracted 83-s after the spout extension. The trial stopped 62-s after the spout retraction. In the meantime, mice also underwent 2 days of training (5 trials/day) with auditory fear conditioning in an electrical foot-shock arena (Coulbourn Instruments, fig. S7D). During the fear conditioning training (fig. S7D), a 20-s auditory CS stimulus (8 kHz tone, 80 dB) was

presented 60-s after the trial onset. After termination of the CS stimuli, mice received a 2-s, 0.75-mA electrical foot shock. The trial stopped 93-s after the foot shock. The BCLS generated the auditory stimuli and controlled the timing of foot shock. For the fear retrieval experiment (fig. S7E), lick spout was extended 30-s after the trial onset. Mice immediately started licking the palatable Ensure. The same CS stimulus (20 s, 8 kHz, 80 dB) was presented 30 s after the spout extension. Mice stopped eating the palatable Ensure immediately after the onset of the CS tone presentation. The lick spout was retracted 33-s after termination of the CS stimuli. The trial stopped 62-s after the spout retraction. In the fear retrieval experiment, suspension of licking after the onset of CS stimuli presentation to the spout retraction (Fear and Tone-Aft state in fig. S7E) was used as an alternative measurement of freezing response to fear. Once mice started eating during CS stimulus presentation (5-6 trials), then the experiment switched from functional trial acquisition to meta trial acquisition. Inter-trial intervals in the experiments above were randomly chosen (90 - 180 s)

Day 6-10: Hormone injections

After the fear retrieval experiment, mice were acclimatized to stay on the treadmill without lick spout for food or water delivery (fig. S7F). Then, ghrelin (1 µg/g, Bachem Americas, Inc.), saline and leptin (1 µg/g, R&D Systems) injection experiments were performed on 3 consecutive days (fig. S7G). In these injection experiments, intraperitoneal injections (ghrelin and leptin were diluted with saline and injected at a volume of 10 µL/g; saline: 10 µL/g) were administered between the 1st and 2nd trials. Each trial is a 175-s-long imaging acquisition. Inter-trial intervals in these injection experiments were 2-min.

Post hoc tissue processing

After all imaging experiments were completed in a mouse, several meta trials were acquired in the live mouse before perfusion (ZStack_Bef_Perf images). To avoid possible circadian effects on gene expression, mice were perfused at the same time of day (2 pm) with PBS and then with 4% paraformaldehyde (PFA). Because the perfusion changes intracellular calcium concentration, the fluorescence intensities of individual neurons can be considerably different before and after the perfusion. To facilitate the *ex vivo* → *in vivo* image registration, we also acquired several meta trials in the perfused lifeless animal (ZStack_Aft_Perf images). As an intermediary between live *in vivo* images and *post hoc* confocal microscopy images, these ZStack_Bef_Perf and ZStack_Aft_Perf images facilitated aligning neuron in the *in vivo* imaging volume with the *ex vivo* cryosections (see below). For brain removal, it was important to take care to maximally preserve the integrity of brain tissue right beneath the lens. For this, the brain was removed out of the skull from the bottom of the skull while the GRIN lens was still fixed on top of the skull. The brain gently slid off the GRIN lens after it was freed from surrounding skull.

After GRIN lens removal, the harvested brain was post-fixed in 4% PFA with RNase Away (Thermo Scientific™ Cat # 7002) for 16 hours, cryopreserved (4 ℃) in 15% and 30% sucrose sequentially, and embedded in Fisher Healthcare™ Tissue-Plus™ O.C.T. Compound (Cat # 23-730-571). Consecutive horizontal cryosections (14 µm in thickness) were collected to encompass the dorsal-ventral distance of *in vivo* imaged volume. Each section was mounted onto an individual Fisherbrand™ Superfrost™ Plus

microscope slide (Cat # 12-550-15) with the same orientation as *in vivo* (ventral side facing the glass slide). All slides were dried overnight at 4°C.

We then proceeded to obtain DAPI and GCaMP images to find the *in vivo* imaged volume on *ex vivo* tissue sections. Sections were moved from 4°C to room temperature (RT), further dried at RT for 30 minutes, fixed with 4% PFA for 30 min on slides, DAPI stained, and imaged on a Zeiss LSM880 NLO upright confocal microscope with a 20× water-dipping objective (1.0 NA). For each section, we first chose a single focal plane in the middle of the optical Z-stack and imaged DAPI ("DAPI full", without DNase I digestion and protease treatment) and GCaMP signals on this plane (fig. S12). This single-plane image encompassed a large area (AP: ~2.66 mm, the entire 3rd ventricle lining on the GRIN lens implant side; ML: ~ 860 µm, larger than the diameter of the GRIN lens). We then acquired a full z-stack of a smaller area (AP: ~1.11 mm; ML: ~ 850 µm) sufficiently covering the GRIN lens track (with a hole) or the field-of-view (FOV) underneath the GRIN lens (Round F in fig. S10). After imaging DAPI and GCaMP, slides were dried and stored at -80°C, while we performed the *ex vivo* → *in vivo* image registration by identifying GRIN lens imaged neurons on confocal microscopy images (See *Ex vivo* to *in vivo* image registration).

Calibration and correction of two-photon-GRIN-lens imaging system

To calibrate the optical properties of our two-photon-GRIN-lens (TPGL) imaging system, we built a calibration setup (fig. S8A). A custom-built one-photon and two-photon excitable grid target (fig. S8B) was held on a micromanipulator (MP285, Sutter Instrument) and aligned with the objective lens. The GRIN lens was aligned with the objective lens using epifluorescence imaging (as described above). Once the grid target, GRIN lens, and objective lens were well aligned, we moved up the grid target until it touched the GRIN lens and set the target position as 0 for sample position. Water was used as immersion medium between GRIN lens and the target. We then moved the objective lens to focus on the top surface of the GRIN lens and set the position as reference zero for the objective lens (air interface, the same as in the *in vivo* imaging experiment). For each objective lens position (270 to 1280 µm; step size: 270-985 µm, 5 µm; 990-1280, 10 µm), we acquired a two-photon image stack of the grid target by changing the target position with the micromanipulator (step size: 1 µm).

An ImageJ script was used to segment and track individual grid elements on the target. Axial resolution at each grid position (fig. S8E) was measured with the full-width at half maximum of the Gaussian fit for the average intensity of that grid along Z-depth. The sample position (Z) at each grid position (red dots in fig. S8F) was measured with maximum of the Gaussian fit for the average intensity of that grid along Z-depth. For each objective lens position, we computed the imaging plane (surface in fig. S8F) with a paraboloid surface fit of the measured sample positions. After we had the grid positions, axial resolutions and imaging planes from all objective lens positions, we fitted the objective-lens-position-dependent mean axial resolution (fig. S8G), vertex of imaging plane (fig. S8H) and relative magnification (fig. S8I). Then, we built a calibration model (fig. S8J) to describe the objective-lens-position-dependent transformation from the TPGL imaging space to real physical space by integrating the fits in (fig. S8, F, H and I). Finally, we corrected all ZStack images acquired in meta trials with the calibration model using the scatteredInterpolant (linear interpolation) function in Matlab.

<u>*Ex vivo* to *in vivo* image registration</u>

Alignment of ex vivo brain slices

To identify the neurons in the *in vivo* imaging volume from the images of *ex vivo* sections, we first reconstructed the imaging volume by stitching the *ex vivo* images. Because the PVH surrounds the 3rd ventricle, we coarsely stitched the large single-plane images of *ex vivo* sections using the contour of the 3rd ventricle. We then used the stitched large single-plane images as a map and coarsely aligned the smaller full z-stack images of sections. The coarsely stitched image stacks have ~200 µm error (mostly in A/P direction) between consecutive sections because of the tissue distortions caused by tissue processing (sectioning, mounting, etc.). We further adjusted the coarsely stitched image stacks, using the corrected *in vivo* ZStack images. With the help of corrected ZStack_Aft_Perf *in vivo* images and GRIN lens track (hole on the superficial sections), we usually could find a few neurons with distinct features (cell body shape, neurites, etc.) in the *in vivo* imaging volume that could be confidently recognized in the *ex vivo* confocal images of each section. For example, in Fig. 3D, we found distinct neuron 29, 30, 32 and 33 in the top slice, neuron 15, 16 and 21 in the middle slice and neuron 1, 2 and 3 in the bottom slice; in fig. S9B, we found distinct neuron 9, 20 and 35 in the top slice, neuron 1, 2 and 4 in the middle slice and neuron 10, 11 and 26 in the bottom slice. We then adjusted relative positions of these coarsely stitched slices so that the spatial patterns of these identified distinct neurons could be well matched between *in vivo* and *ex vivo* images (left columns in Fig. 3D and fig. S9B). Stitching of *ex vivo* images was performed manually using TrakEM2 with rigid transformation.

Aligning neuron ROIs between ex vivo and in vivo image volumes

After stitching *ex vivo* images, we used these identified distinct neurons as starting points to find their neighbor neurons matched between the corrected ZStack images in fear retrieval experiment (middle of the entire imaging experiments) and *ex vivo* images (right columns in Fig. 3D and fig. S9B). In addition, the matched neurons were mapped to the FastZ imaging planes from the corrected ZStack images. The ROIs of the matched neurons (*ex vivo* → correct ZStack → FastZ) were manually drawn in 3D using TrakEM2. Using this *ex vivo* to *in vivo* image registration strategy, two independent observers found the neurons matched between *in vivo* and *ex vivo* with 96% correspondence. Identifications of all neurons for further analyses were discussed and confirmed by these two independent observers.

<u>Multiplex FISH in CaRMA-imaged tissue after *in vivo* calcium imaging</u>

After *in vivo* to *ex vivo* registration, we performed 12-plex FISH on sections where *in vivo* imaged neurons had been found to reside using the FISH protocol as described in (<u>Multiplex FISH of PVH neurons in FISH-only tissue</u>) with the following 3 modifications.

These modifications resulted from GCaMP-expressing neurons in CaRMA-imaged tissue. First, after 4-hour DNase I digestion before FISH, we re-applied DAPI stain and then imaged all sections for DAPI and GCaMP fluorescence (Round P in fig. S10). DNase I digestion prior to tissue permeabilization generated a DAPI pattern that we called "DAPI partial" (with DNase I digestion but without protease treatment). This "DAPI partial" pattern was used as an intermediary between a "DAPI full" pattern

obtained in the earliest confocal imaging session and a “DAPI residual” (after DNase I digestion and protease treatment) pattern obtained after DNase I was used to strip FISH oligonucleotides on fully permeabilized tissue sections. This intermediate pattern was necessary for registering GCaMP expression and marker-gene expression of the same neurons (fig. S10, B to D). Second, to eliminate GCaMP fluorescence in the 488 nm channel, SSC buffered citric acid (1 volume of citric acid added into 9 volumes of 1.18X SSC, yield a pH between 5.6 and 5.7) was used as immersion media during FISH imaging. This pH was sufficiently acidic to quench GCaMP6m fluorescence. Third, because we already had ROIs of GCaMP-expressing neurons, the round of DAPI and Syto staining after FISH that we used for FISH-only tissue was removed for calcium activity imaged tissue.

Image processing of multiplex FISH in CaRMA-imaged tissue

Multi-round registration of brain slices using DAPI fluorescence for multiplex FISH

After *ex vivo* to *in vivo* image registration and multiplex FISH in the same brain tissue, we generated gene expression profiles of individual neurons registered between *ex vivo* and *in vivo* (fig. S10B). Matlab scripts based on ANTs toolkit (*63*) (http://picsl.upenn.edu/software/ants/) running in Janelia Computer Cluster were used for 3D image registrations and 3D image transformations. We used the image stack of DAPI residual from Round 1 as reference image stack for the 3D registration. Because of the change in the shape of DAPI staining before (DAPI full from Round F) and after (DAPI residual from Round 1) DNase I digestion and protease treatment, we aligned DAPI full with the reference DAPI residual in 2 steps to improve the registration precision (step 2 in fig. S10B). We first aligned the image stack of GCaMP from Round F with the image stack of GCaMP from Round P (fig. S10C) and obtained its corresponding transformation (*Transformation_F*). We then aligned the image stack of DAPI partial from Round P with the image stack of DAPI residual from Round 1 (fig. S10D) and obtained its corresponding transformation (*Transformation_P*). Next, we transformed the neuron ID identified in the GCaMP image from Round F (3D ROIs drawn during *Ex vivo* to *in vivo* image registration, step 1 in fig. S10B) to aligned neuron ID (aligned 3D ROIs) using the concatenated transformation (*transformation_FP*, the concatenation of *transformation_F* and *transformation_P*, step 3 in fig. S10B). In addition, we aligned the image stacks of DAPI residual from Round 2-4 with the reference image stack (step 4 in fig. S10B and fig. S10D) and obtained their corresponding transformations (*Transformation_Ri*, i = 2 to 4). Images of FISH signals probed in Round 2-4 were transformed to aligned images of FISH signals using their corresponding *Transformation_Ri* (step 5 in fig. S10B). Finally, we merged the aligned neuron ID and aligned FISH signals to generate an image stack of gene expression profiles of individual neurons (step 6 in fig. S10B).

Multi-round FISH signal extraction, issues with AAV tropism

Three features (voxels, mean intensity and sum intensity, where sum intensity = voxels × mean intensity) of each mRNA signal were extracted from individual neurons as described for FISH-only tissue. Then, these extracted FISH signal features were manually validated by visual inspection. Finally, these 3 features of each mRNA transcript signal were further normalized to [0 1] in the same way as for FISH-only tissue.

We did not observe GCaMP expression in *Oxt*$^+$ and *Avp*$^+$ neurons in the CaRMA tissue because of the viral tropism of the AAV serotype 1 that we used. The viral tropism was confirmed by the evidence that we found GCaMP expression in *Oxt*$^+$ and *Avp*$^+$ neurons by injecting AAV-DJ-EF1a-DIO-GCaMP6m (fig. S15, GVVC#: GVVC-AAV-92, Stanford Gene Vector and Virus Core). In general, this is a potential challenge with CaRMA imaging where AAV tropism issues may arise for capturing all cell types, thus the broadest neural tropism is advantageous for this technique. We only imaged a few *Sst*$^+$ neurons because *Sst*$^+$ neurons are distributed along the 3rd ventricle in the PVH (Fig. 2G, and fig. S6, A and B), which is not accessible to GRIN lens placements without damaging the ventricle. Thus, we used the gene expression profiles of the remaining 9 genes (without *Oxt*, *Avp* and *Sst*) to cluster all neurons into molecularly defined cell types (Fig. 5B).

Classification of molecular clusters (MCs) from multiplexed FISH data

Unsupervised hierarchical clustering was used to classify these neurons using their sum intensities of the 9 genes (without Oxt, Avp and Sst; z-score normalized values). The optimal cluster number 11 was chosen with 3 criteria 1). Calinski-Harabasz index; 2). Aggregation of neurons with clusters by visual inspection of t-SNE plot; 3). Identification of cluster 11 with the only 2 high Sst-expressing neurons, even though Sst was not used for clustering (Fig. 5B). tSNE visualization of these molecularly defined cell types (MCs, Fig. 5C) were based on their sum intensities of the 9 genes (z-score normalized values) using tsne function in Matlab (Distance = correlation, Standardize = true, Perplexity = 15).

To evaluate the validity of molecular clustering of the CaRMA-imaged neurons, we combined the gene expression profiles from pPVH FISH-only and CaRMA imaged tissue as a merged multiplex FISH dataset and classified the neurons in the merged dataset into 13 molecular clusters (posterior PVH Clusters, pCs) using their sum intensities of the 12 genes (z-score normalized values, fig. S16A). Next, for each MC, we found its corresponding pC according to their high-expressed genes or "Low". For all CaRMA-imaged neurons, we used their MCs as true classes and their corresponding pCs as predicted classes and computed the confusion matrix. We defined this confusion matrix as a consistency matrix representing the consistency between the molecular clusters determined with CaRMA dataset and the clusters determined with the merged FISH dataset (fig. S16C). Consistency ratio of a CaRMA cluster is the recall of the confusion matrix for that CaRMA cluster, while consistency ratio of a cluster from the merged datasets is the precision of the confusion matrix for that cluster (fig. S16D).

Threshold for "positive" gene expression

We considered a neuron as being positive for expression of a gene *xxx* (*xxx*$^+$) using the same criterion as for FISH-only tissue.

<u>*In vivo* calcium imaging preprocessing</u>

Alignment of in vivo image volumes across multiple behavioral states

For the FastZ images in each imaging plane, brain tissue movement under the GRIN lens was corrected using Matlab scripts based on ANTs toolkit in Janelia Computer Cluster. To improve the signal-noise ratio for further analyses, the motion corrected images were temporally downsampled by averaging every 3 frames to 1 frame (temporal sample rate: 7.75 Hz → 2.58 Hz).

For each imaging experiment, the average intensity projection image of the motion-corrected FastZ images in each imaging plane was used as the template image of that imaging plane. For the fear retrieval imaging experiment, the ROIs of the *ex vivo → in vivo* matched neurons were manually drawn on these template images (see *Ex vivo* to *in vivo* image registration). To longitudinally track the same neurons in different imaging experiments, the template image of each imaging plane in the fear retrieval experiment was aligned with its corresponding template images in other experiments using Matlab scripts based on ANTs toolkit. This template from fear retrieval experiment was selected for alignment across states because it was midway through the sequence of behavioral state experiments. To get the neuron ROIs in other experiments, the alignment transformations (linear and nonlinear) were applied to the ROI images of individual corresponding imaging planes. Next, these neuron ROIs in other imaging experiments were overlaid with their template images, manually examined and corrected.

Removal of overlapping ROI areas and contamination from background signals

Because of the relative elongated axial resolution (~10 µm, fig. S8G) of our TPGL imaging system, the images of some neurons may be overlapped (fig. S11A). To prevent the signal interference among these overlapped neurons, cleaned ROIs (ROIs_clean) were generated for individual neurons by excluding the overlapped region from the ROIs of these overlapped neurons (fig. S11C). To correct the contamination from background signals (*64*), the calcium fluorescence signal of neuron *i* was measured as $F_i(t)=F_{ROI\_clean\_i}(t)-F_{Bkg\_i}(t)$, where $F_{ROI\_clean\_i}(t)$ is the average fluorescence intensity within the ROI_clean of neuron *i*, while $F_{Bkg\_i}(t)$ is the average fluorescence intensity within a ~ 20 µm (20 pixels in the FastZ images) region from the neuron center (excluding all ROIs, fig. S11, B and C).

ROC analysis of neuron calcium fluorescence responses

We used receiver-operating characteristic (ROC) analysis to measure neuron temporal responses (*38*). The temporal response of neuron *i* was calculated as $R_i(t) = 2\times(auROC_i(t)-0.5)$, where $auROC_i(t)$ is the area under the ROC curve using fluorescence signals {$F_i(base)$, $F_i(t\_wnd)$} to discriminate current state from baseline state. $F_i(base)$ were fluorescence signals of neuron *i* during a baseline period, while $F_i(t\_wnd)$ were fluorescence signals within a sliding time window at time *t*. The baseline period was 20-40 s after trial onset across trials in hunger eating and thirst drinking experiments, 40-120 s of the first trial in the injection experiments. The sliding time window was 2-s (centered at time *t*) across trials in hunger eating, thirst drinking and fear retrieval experiments (multiple repeat trials) and 8-s (centered at time *t*) in the injection experiments (singular continuous trials). The range of auROC value is [0 1], where 0.5 indicates no responses. To calculate consistent-response by scaling the responses with purity, we need to set response metric = 0 for no response. Thus, we transformed the auROC value to 2×auROC-1 as the response metric. In addition, for neural activity data, the transformed auROC has a range [-1 1], which is also intuitive with widely used notations of neuron inhibition depicted with negative values and activation with positive values. We called the transformed auROC as t-auROC and set $R_i(t) = t\text{-}auROC_i(t)$. $R_i(t) = 1$ represents reliable activation (all fluorescence signals within the time window from all trials were greater than all fluorescence signals during the baseline period from all trials). $R_i(t) = -1$ represents reliable inhibition (all fluorescence signals within the time window from all trials were less than all fluorescence signals during the baseline period from all trials).

$R_i(t) = 0$ indicates no response (the distribution of fluorescence signals within the time window from all trials was similar to the distribution of all fluorescence signals during the baseline period from all trials). In general, $R_i(t) > 0$ indicates activation, while $R_i(t) < 0$ indicates inhibition. In addition, this measurement of neuron temporal response normalizes neuronal responses by considering the response amplitude and incorporates reliability of response changes across trials between time *t* and baseline.

ΔF/F activity map in Movie S1

ΔF/F was calculated for each pixel's time series as described in (65) with minor modifications. Briefly, for each pixel, baseline fluorescence (F_{bs}) was estimated with the 20th percentile of the pixel's time series. For pixel *i* at time *t*, ΔF/F (*i*,*t*) = ($F_{raw}(i,t)$ – F_{bs} - 50)/(F_{bs}+20), where $F_{raw}(i,t)$ is the raw fluorescence intensity of pixel *i* at time *t*. The addition of 20 to the denominator was used to prevent division by baseline values very close to zero. The subtraction offset to the numerator (-50, similar results were observed by using other values in [-20 -100]) and addition offset to the denominator (+20, similar results were observed by using other values in [10 50]) of the ΔF/F calculation underestimates true ΔF/F but reduces the rate of artifacts. To generate the ΔF/F activity images, the ΔF/F activity pixels in each frame were filtered with a Gaussian filter (radius = 2 pixels).

Analysis of *in vivo* imaging responses

Unsupervised clustering of behavioral states

For unsupervised clustering behavioral states based on their neuronal ensemble responses without regard to cell type (Fig. 4A), responses of each neuron in individual behavioral states were temporally averaged into 26 bins (26-bin responses, the minimum number of timepoints among all states). The binned responses of all neurons (319 neurons) in each state were concatenated to a response vector (26 × 319 data points) as neuronal response representation of that state. Hierarchical clustering (correlation distance and single linkage) generated the dendrogram of behavioral states similarities based on their neuronal response representations.

Screening for labeled-line neurons

To search for labeled-line neurons encoding the 11 behavioral states (Fig. 4B), a labeled-line neuron was defined as specifically activated or inhibited in one state but not in other states. Neuron *i* was assigned as activated in behavioral state S_n using the criterion $Rm\_S_{n,i} – Rm\_base_i > 3×Std\_base_i$, where $Rm\_S_{n,i}$ is the mean response of neuron *i* in state S_n, Rm_base_i is the mean response of neuron *i* in baseline state and Std_base_i is the standard deviation of the responses of neuron *i* in baseline state. In contrast, neuron *i* was assigned as inhibited in behavioral state S_n using the criterion $Rm\_base_i – Rm\_S_{n,i} > 3×Std\_base_i$. The baseline state was before the lick spout extension in Hunger-Eat, Thirst-Drink and fear retrieval experiments and was the 1st trial in the injection experiments. For each *k*-state combination from all 11 choose *k*-combinations (C_{11}^{k}) (*k* = 2 to 11), we searched for labeled-line neurons among all 319 neurons and counted the number of labeled-line neurons (n_{ln}) and the number of behavioral states (n_s) encoded by these labeled-line neurons. The *k*-state combination with maximum n_s and maximum n_{ln} (n_s was prior to n_{ln}) in all C_{11}^{k} *k*-state combinations was displayed in Fig. 4B to indicate the maximum number of labeled-line neurons for encoding *k*-states.

To search for labeled-line neurons encoding behavioral state-classes, we grouped these 11 behavioral states (S_n, n = 1 to 11) into m "state-classes" (SC_j, j = 1 to m) either subjectively based on ideas about behavioral similarity (m = 7, fig. S13A) or systematically based on response similarity metrics derived from the PVH neural ensemble used for unsupervised clustering (m = 2 to 11, Fig. 4A). Subjective grouping was based on the rationale that 1) food consumption behaviors might be similar, 2) a shock-associated CS often elicited fear-like freezing responses during its delivery and shortly after the CS is terminated, and 5) leptin is thought to mimic aspects of an energy surfeit state, so it was grouped with post-food consumption states.

Systematic grouping was performed as follows. Given $SC_j = \{S_{j1}, \ldots, S_{js}\}$, neuron i was assigned as activated or inhibited in state-class SC_j when it was activated or inhibited in all element behavioral states ($S_{j1}, \ldots, S_{js}$), respectively. For each k-state-class combination from all m choose k-combinations (C_m^k) (k = 2 to m), we exhaustively searched for labeled-line neurons among all 319 neurons and counted the number of labeled-line neurons (n_{ln}) and the number of state-classes (n_{sc}) encoded by these labeled-line neurons. The k-state-class combination with maximum n_{sc} and maximum n_{ln} (n_{sc} was prior to n_{ln}) in all C_m^k k-state-class combinations was used to indicate the maximum number of labeled-line neurons for encoding k-state-classes. fig. S13 and fig. S14 are examples of searching for labeled-line neurons encoding 7 behavioral state-classes.

Purity and consistent-response

Response purity is a linearly normalized entropy used to measure the response similarity of a group of neurons (fig. S17). The value of maximum entropy (3 bits) was determined by $floor(log_2(min\_nSamp))$, where *min_nSamp* is the minimum sample size from all groups (*min_nSamp* = 9 for MC5-Crh neurons). Examples and formulas for calculating response purity and consistent-response were shown in fig. S17B. When computing the consistent-responses of single gene defined cell types, we assigned a neuron as *xxx* cell type (xxx^+) using a criterion $Voxel\_xxx > 4$, where *Voxel_xxx* is the number of voxels with FISH signal of gene *xxx* in that neuron. The same criterion was applied to gene co-expression pattern of *Crh* subtypes (fig. S20A).

Decoding behavioral states with neuronal responses

Multinomial logistic regression with L1 regularization from Glmnet in Matlab toolbox (http://web.stanford.edu/~hastie/glmnet_matlab/index.html) was used to decode behavioral states with neuronal responses. To explicitly include the neuronal dynamics in these prediction models, we used the binned temporal responses (26 response bins in each state as described in *Unsupervised clustering of behavioral states*) of neurons as the predictor inputs of the multinomial regression. To assess the generalization ability of these prediction models, all prediction performance metrics were measured with cross-validation (leave-one-out or 10-fold as noted below)

Decoding behavioral states with responses of a single molecularly defined cell type

For decoding behavioral states with the neuronal responses from cell type MC_m (m = 0 to 10; MC_0 is all neurons, which ignores cell type; Fig. 6A), each labeled sample in the dataset (DS_single) consisted of a response array as input (X_i) and a label as output (Y_i). X_i is the 26-bin temporal responses of a MC_m neuron $MC_m\_N_j$ in behavioral state B_k, where j = 1 to MC_m_nSamp and *MCm_nSamp* is the number of MC_m neurons (*MC0_nSamp = 319*). Y_i is the label of behavioral state B_k ($Y_i = k$, k = 1 to 11). Thus, the

sample size of DS_single (*Ds_single_nSamp*) is 11*MCm_nSamp and i = 1 to *Ds_single_nSamp*. Decoding performance, including accuracy (Fig. 6B), confusion matrix (Fig. 6C) and F1 score (fig. S21A), was evaluated by leave-one-out cross-validation using DS_single dataset.

Decoding behavioral state by 10-neuron ensembles combining one of each cell type

For decoding behavioral states with the combined responses of each PVH molecularly defined cell type (Fig. 6E), each labeled sample in the dataset (DS_comb) consisted of a combined response array as input (X_i) and a label as output (Y_i). X_i is the 10-cell-ensemble, the concatenated responses of 10 neurons (a 260-long array; 10 neurons, each with 26 time bins), which were randomly chosen by 1 neuron from each cell type, in behavioral state B_k. Y_i is the label of behavioral state B_k ($Y_i = k$, k = 1 to 11). We randomly chose 100 (X_i, Y_i) pairs with replacement for each behavioral state (the same neuron combinations but with different responses from different states). This resample size (100 pairs) was determined by *10*ceil(max_nSamp/10)*, where *max_nSamp* is the maximum sample size of PVH cell types (*max_nSamp* = 92 for MC7-Low neurons) and 10 is from 10-fold cross-validation. Thus, the sample size of DS_comb is 1100 and i = 1 to *1100*. For statistical analysis, we generated 2000 DS_comb datasets and evaluated each for decoding performance metrics, including confusion matrix (Fig. 6F) and accuracy (Fig. 6G), by 10-fold cross-validation using individual DS_comb datasets.

For decoding behavioral states with the combined responses of the dummy PVH cell types (fig. S21B), we first shuffled all neurons to generate 10 dummy PVH cell types. The sample size of each dummy cell type is the same as that of its corresponding real cell type, but the dummy cell type consists of a random selection from all PVH neurons independent of molecularly defined cell type identity, thus this dummy cell type could include any of the different molecularly defined PVH cell types. Next, the dataset (DS_dummy) was generated from the dummy PVH cell types using the same procedure as that with the real cell types (as described in the preceding paragraph). We repeated the DS_dummy dataset generation following dummy cell types generation 2000 times to facilitated statistical comparisons. Finally, we evaluated the decoding accuracies by 10-fold cross-validation for each of the 2000 DS_dummy datasets (accuracy: 84.20 ± 0.04%) and compared these accuracies with those generated using real cell types (Fig. 6G). Because the analyses of decoding behavioral states with the combined responses (real and dummy) were computationally intensive, they were coded with Matlab running in Janelia Computer Cluster. Predictive accuracy using 10-cell-ensembles in the absence of molecularly defined cell type information was consistent with past results decoding behavioral states using neuronal ensemble dynamics without molecular information (*3, 66, 67*) and was better than decoding accuracy from a single cell (Fig. 6B). However, taking cell type information into account gave significantly greater predictive accuracy than when molecularly defined cell type information is scrambled (p=0), which has not been previously observed because it had not been possible to simultaneously evaluate activity patterns in many molecularly defined cell types (Fig. 6G).

Effect of the number of molecular clusters on the robustness of behavioral state decoding

To examine the effect of the number of molecular clusters on the accuracy of behavioral state decoding, we systematically adjusted the threshold for hierarchical molecular clustering in Fig. 5B and classified the CaRMA-imaged neurons into m cell types (m = 1 to 13). If the Sst cluster with only 2 neurons was one of the m cell types, it

was excluded from the decoding analysis. Thus, the effective number of cell types *n* = *m* or *m-1* (fig. S23A).

Next, we decoded the behavioral states with the combined neuronal dynamics of *n* neurons from the *n* cell types (one from each cell type) using the same procedure as in *Decoding behavioral state by 10-neuron ensembles combining one of each cell type* but different number of cell types. Similarly, we also shuffled the neurons to generate *n* dummy cell types and performed behavioral state decoding analysis with the combined neuronal dynamics of *n* neurons from the *n* dummy cell types (one from each dummy cell type). Improvement in decoding accuracies by using the molecular information was the difference between mean decoding accuracies with PVH cell types and dummy cell types.

Response-decoding diagrams

Response-decoding diagrams illustrate the temporal maximum of consistent-response quantitatively using line width. The temporal maximum was used because the purity and response amplitude can change with time, based on the characteristic dynamics of a molecularly defined cell type, but the maximum consistent-response is likely to have greatest relevance to the capacity of a cell type to contribute to behavioral state coding.

The decoding weights for each molecular cluster (MC) in each behavioral state were shown as proportional to arrowhead size. These weights were derived from the average multinomial regression coefficients for the temporal response (26 bins) of individual cell types in each behavioral state (average value of the corresponding coefficients from the 2000 prediction models). These average coefficients were transformed into decoding weights of a given molecularly defined cell type for each behavioral state by the summation of the absolute values of the average decoding coefficients of that cell type for that behavioral state. Then the decoding weights of all cell types for each behavioral state were normalized, so that the summation of the normalized decoding weights of all cell types for each behavioral state was 1.

Clustering of PVH neuron functional responses measured by CaRMA imaging

For functional clustering of neurons based on their responses in a behavioral state (Fig. 7, A and B, and fig. S26), we first denoised the responses by dimensionality reduction using principle component analysis (PCA) to extract the most important *n* principle components (PCs), where *n = max(3, n_ev90)*, *n_ev90* is the number of PCs that could explained >90% variance of the responses. We then used unsupervised hierarchical clustering to classify these neurons based on the *n* PCs of their responses (Distance = correlation). The optimal cluster number of 2 in every behavioral state was chosen with Silhouette index.

For functional clustering neurons based on their concatenated responses across all behavioral states (fig. S31A), to balance the weight of response in each behavioral state, we first temporally averaged the response in each behavioral state into 26 bins (26-bin responses, the minimum number of timepoints among all states) and concatenated them across all behavioral states for each neuron. PCA was used to reduce the dimension of concatenated responses to the most important 4 important PCs which explained 75% variance of the concatenated responses. We then used unsupervised hierarchical

clustering to classify these neurons based on these 4 PCs of their responses (Distance = correlation). The optimal cluster number, 5, was chosen with Calinski-Harabasz index.

Prediction of functional clusters

We developed a quantitative analytic approach to predict the functional cluster of each neuron solely using its gene expression profile. The quantitative analysis had 4 highlighted characteristics. 1) Gene expression levels were taken into account by 3 normalized features of each gene. 2) Multiple rounds of sequential forward feature selection (mSFFS) were used to identify the optimal gene combinations that best predicted the functional clusters while minimizing the contribution of redundant gene co-expression information. 3) Predictive powers of gene expression profiles for functional clusters were statistically evaluated by shuffling the relationship between gene expression of individual neurons and their functional clusters. 4) All prediction performances were measured with leave-one-out cross-validation to assess the generalization ability of the predictions.

Normalization of gene expression features

For most machine learning algorithms, normalizing input features to normal distribution with similar scale range improves the algorithm performance. We used 3 features (voxel, mean intensity and sum intensity) to represent the expression level of each gene. We found that the raw mean intensity features of all genes largely had normal distributions with a big gap between feature value = 0 and value > 0, but the raw voxel and sum intensity features of most genes were not normally distributed (fig. S29A). To improve the algorithm performance, we normalized these features with *N01_ReLu_ln* function (fig. S29B). This function achieved 3 goals of feature normalization (fig. S29C). 1) The voxel and sum intensity features became largely normal distributed. 2) The dynamic range of mean intensity features was increased. 3) All features were in the same scale range [0 1].

Prediction of 2 functional clusters in each behavioral state

For each behavioral state, we used logistic regression with L1 regularization from Glmnet in Mablab toolbox (http://web.stanford.edu/~hastie/glmnet_matlab/index.html) to predict the functional cluster of each neuron solely using its gene expression profile (Fig. 8, A and B). For Neuron *i* in behavioral state B_k (i = 1 to 319, k = 1 to 11), we used its normalized gene expression features ($G_i = (g_{1i}, g_{2i} \dots g_{9i})$, where $g_{mi} = (gf_{mV\_i}, gf_{mM\_i}, gf_{mS\_i})$ are the 3 normalized expression features of gene m (g_m, m = 1 to 9)) as input and its functional cluster ($FC_{ik} \in$ {Act-FC, Inh-FC}) as output. Thus, for behavioral state B_k, its sample dataset (DS_B_k) consisted of 319 pairs of (G_i, FC_{ik}) (fig. S30A). All prediction performances, including confusion matrix, area under the receiver-operating characteristic (auROC) curve and accuracy, were measured with leave-one-out cross-validation using DS_B_k dataset to assess the generalization ability of the predictions.

Multiple rounds of sequential forward feature selection (mSFFS)

We developed mSFFS to optimize the prediction performance and quantitatively assess the importance of individual genes and their combinations in predicting neuron functional clusters. The mSFFS assessed the importance of a gene in predicting functional clusters by gain-of-function (add it in) and loss-of-function (remove it) manipulations of its expression features (add in or remove its 3 expression features together) in the prediction models. In the 1st round of mSFFS, we used all genes as

candidate gene set ($GC^1 = \{g_1, g_2 \ldots g_9\}$, where g_m is represented by the 3 normalized features of gene m (m = 1 to 9) from all neurons (319×3 matrix).). We then sequentially added 1 gene from the candidate gene set to the select the best gene set (GS^1, gain-of-function analysis), so that the combination of the normalized features of the selected genes had highest accuracy. We continued adding 1 gene into GS^1 until there was no improvement in prediction accuracy. In the nth (n = 2 to 8) round of sequential forward feature selection, the most predictive gene from the previous round (gp_j, $j<n$) was removed from the candidate gene set ($GC^n = \{g_1, g_2 \ldots g_9\} - \{gp_1, gp_2 \ldots gp_{n-1}\}$, loss-of-function analysis of $\{gp_1, gp_2 \ldots gp_{n-1}\}$). We obtained the selected best gene set (GS^n) from GC^n using the same sequential forward feature selection as that in Round 1. The selected best gene set (GS^n, n = 1 to 8) contains the optimal gene combinations that best predicted the functional clusters while minimizing the contribution of redundant gene co-expression information in GC^n. To make it easy to evaluate the predictive power of any sequential combination of the selected genes, the predictive power in each round of mSFFS was shown as stacked bar graphs with the sequentially additive contributions of the selected genes (Fig. 8C and fig. S30B).

Statistical evaluation of predictive power

To statistically evaluate the predictive power of gene expression profiles for predicting functional clusters, for each behavioral state, we shuffled the relationship between gene expression of individual neurons and their functional clusters to generate a shuffled sample dataset (DS_shuffle). Prediction accuracy was calculated using the DS_shuffle dataset as described for the real dataset. We repeated the shuffling and prediction procedure 2000 times and obtained 2000 shuffled prediction accuracies. The 95th percentile of the shuffled accuracies was set as the statistical significance threshold for $p < 0.05$.

Average coefficients of the SFFS1 optimal genes

For each FC, the average coefficient of gene *xxx* (Fig. 8C) was the average value of coefficients of *xxx*-V, *xxx*-M and *xxx*-S from logistic regression. Positive average coefficient of gene *xxx* for FC*y* indicated the expression of gene *xxx* positively related to the prediction probability as FC*y*. To show how gene combinations improved prediction, only positive coefficients were plotted using 2 colors to indicate their corresponding FCs.

Prediction of 5 functional clusters across all behavioral states

Multinomial logistic regression with L1 regularization from Glmnet in Mablab toolbox (http://web.stanford.edu/~hastie/glmnet_matlab/index.html) was used to predict the function cluster across all behavioral state of each neuron solely using its gene expression profile. For Neuron i (i = 1 to 319), we used its normalized gene expression features (G_i, as described in *Prediction of the 2 functional clusters in each behavioral state*) as input and its functional cluster ($FC_i \in \{FCn \mid n = 1 \text{ to } 5\}$) as output. Thus, the dataset for all behavioral states (DS_All) consisted of 319 pairs of (G_i, FC_i) (fig. S31A). All prediction performances were measured with leave-one-out cross-validation using DS_All dataset. We optimized the prediction performance, quantitatively assessed the importance of individual genes and their combinations in predicting their functional clusters across all behavioral states and statistically evaluate the predictive power of gene expression profiles as described above.

Optimal expression threshold of the most predictive gene

The optimal expression threshold of the most predictive gene was systematically evaluated to improve the precision of functional classification. The most predictive gene (*gp*) is the first selected gene in the 1st round of mSFFS. For each behavioral state, the functional cluster with larger number of gp^+ neurons was the target functional cluster (tFC) for gene *gp*. Next, we computed the ROC curve using *gp* expression level (normalized sum intensity of *gp*) of gp^+ neurons to predict *tFC*. The Youden's optimal cutpoint of the ROC curve was calculated. We then used the Youden's optimal cutpoint as *gp* expression threshold to classify all gp^+ neurons. To mimic the target precision for targeting the functional class that would be appropriate for hypothetical optogenetic or chemogenetic manipulation, the prediction precision was used to measure the prediction performance. For prediction of tFC, the precision using optimal *gp* expression threshold is $nTP/(nTP+nFP)$, where *nTP* is the number of true positive neurons and *nFP* is the number of false positive neurons, while the precision using all gp^+ neurons is $n\_gp^+\_tFC/n\_gp^+$, where $n\_gp^+\_tFC$ is the number of gp^+ neurons in tFC, and n_gp^+ is the total number of gp^+ neurons.

Prediction of temporal responses

We examined how well the temporal responses of the PVH ensemble could be predicted solely by their gene expression profiles. Linear regression with L1 regularization from Glmnet in Mablab toolbox (http://web.stanford.edu/~hastie/glmnet_matlab/index.html) was used to directly predict temporal responses of each neuron solely using its gene expression profile (Fig. 9, A, C and E). Prediction of temporal responses was implemented on prediction of the neuronal responses at each timestamp (0.4 s interval) and was repeated for the entire timecourse of behavioral measurements.

Direct prediction of neuronal responses at each timestamp

At timestamp *t*, for Neuron *i* (*i* = 1 to 319), we used its normalized gene expression features (G_i, as described in *Prediction of the 2 functional clusters in each behavioral state*) as input and its functional response ($FR_{ti} \in [0\ 1]$) as output. Thus, for timestamp *t*, the sample dataset (DS_R_t) consisted of 319 pairs of (G_i, FR_{ti}). Fraction of deviance explained (FDE) was used as prediction performance metric and measured with leave-one-out cross-validation using DS_R_t dataset to assess the generalization ability of the predictions. The formula to calculate FDE at timestamp *t* was $FDE_t = 1 - var(R_t-Rp_t)/var(R_t)$, where R_t are the responses of all neurons, Rp_t are the corresponding predicted responses from validation set and *var* is the variance function.

We used mSFFS to optimize the prediction performance, quantitatively assess the importance of individual genes and their combinations in predicting the neuronal responses at each timestamp and statistically evaluate the predictive power of gene expression profiles by shuffling the relationship between G_i and FR_{ti}. mSFFS and statistical evaluation of predictive power were implemented as described in Prediction of functional clusters with 2 modifications. The first one was changing the model from logistic regression to linear regression. The second one was changing the prediction performance metric from accuracy to FDE. Because both analyses for all timestamps were computationally intensive, they were coded with Matlab running in Janelia Computer Cluster. Once we had the optimal predictions of neuronal responses from mSFFS (fig. S33A), we calculated the FDE for each neuron along the entire time series

across behavioral states. To balance the weight of each behavioral states in the FDE calculation, for each behavioral state, we temporally averaged the neuronal responses and their predictions into 26 bins (Rb and Rpb, 26 response bins per state). The formula to calculate FDE for neuron *i* was $FDE_i = 1 - var(Rb_i\text{-}Rpb_i)/var(Rb_i)$, where Rb_i are the binned responses of neuron *i* from all behavioral states, Rp_i are the corresponding binned predicted responses from validation set and *var* is the variance function. The 95th percentile of the 2000 shuffled FDEs at each timestamp (2000 shuffled FDE_t) or for each neuron (2000 shuffled FDE_i) was set as the statistical significance threshold for $p < 0.05$ (Fig. 9, A and D).

Response specificity of a cell type defined by single gene

To investigate how the predictive power of gene *xxx* was related to the underlying temporal dynamics of xxx^+ neurons, we defined and calculated response specificity of xxx^+ cell type at each timestamp. The purpose of defining response specificity was to evaluate how specific the responses of xxx^+ neurons are among the responses of all neurons (xxx^+ and xxx^- neurons). At timestamp *t*, we computed the ROC curve using the responses of all neurons to identify xxx^+ neurons and measured its auROC ($auROC_t$). Response specificity at timestamp *t* (RS_t) was defined as $RS_t = 2\times auROC_t - 1$ (fig. S33C). We then calculated the correlation coefficient between the response specificities with the FDEs of the neuronal response predictions using gene *xxx* at all timestamps within a behavioral state (fig. S33B).

Statistics

Statistical analysis was performed in Matlab and summarized in Table S2. P-values were corrected for multiple comparisons. Unless stated otherwise, data was presented as mean ± SEM.

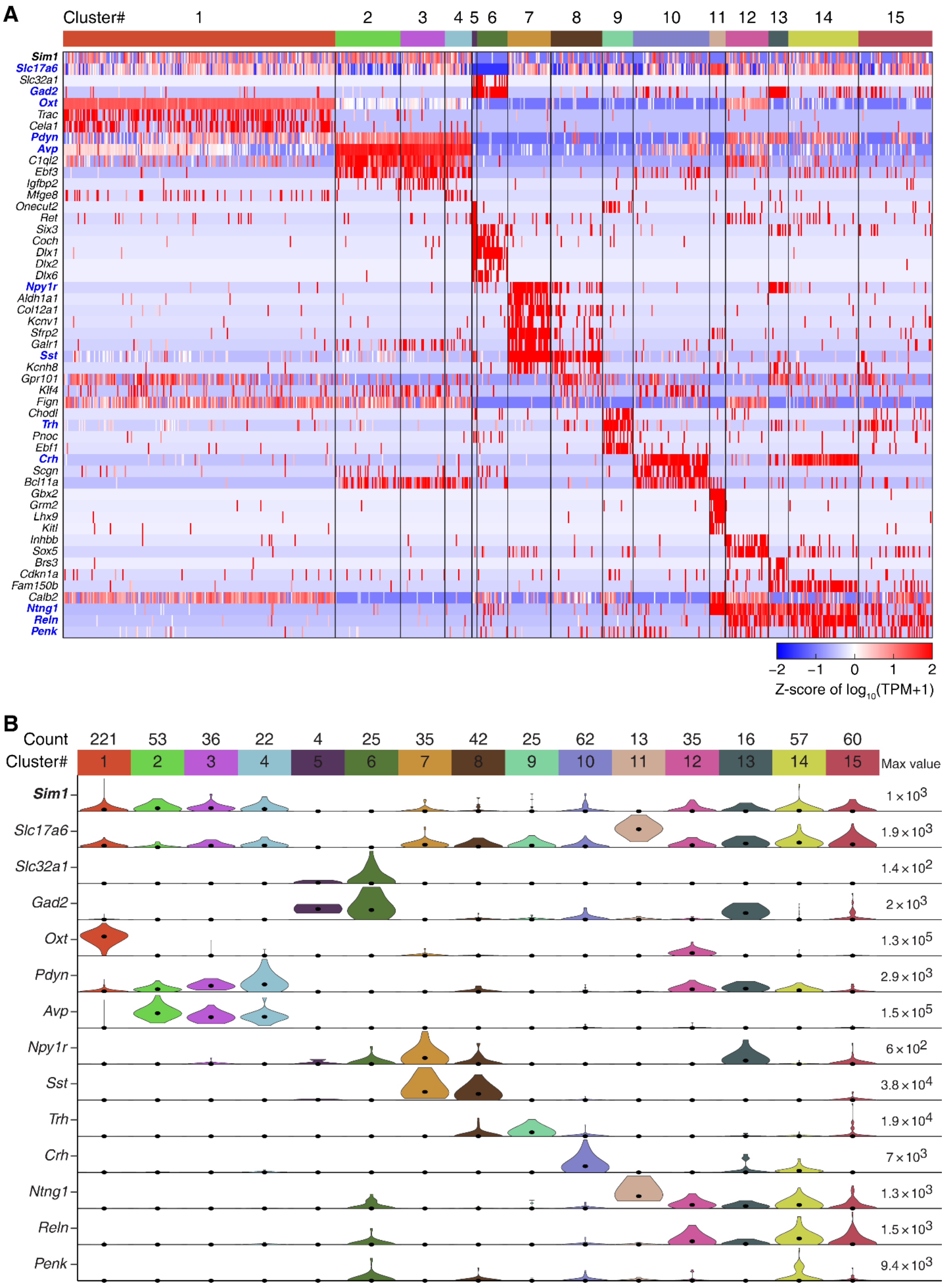
A
Cluster#
1 2 3 4 5 6 7 8 9 10 11 12 13 14 15
Sim1
Slc17a6
Slc32a1
Gad2
Oxt
Trac
Cela1
Pdyn
Avp
C1ql2
Ebf3
Igfbp2
Mfge8
Onecut2
Ret
Six3
Coch
Dlx1
Dlx2
Dlx6
Npy1r
Aldh1a1
Col12a1
Kcnv1
Sfrp2
Galr1
Sst
Kcnh8
Gpr101
Klf4
Fign
Chodl
Trh
Pnoc
Ebf1
Crh
Scgn
Bcl11a
Gbx2
Grm2
Lhx9
Kitl
Inhbb
Sox5
Brs3
Cdkn1a
Fam150b
Calb2
Ntng1
Reln
Penk
−2 −1 0 1 2
Z-score of log10(TPM+1)
B
Count 221 53 36 22 4 25 35 42 25 62 13 35 16 57 60
Cluster# 1 2 3 4 5 6 7 8 9 10 11 12 13 14 15
Max value
Sim1 1 × 10^3
Slc17a6 1.9 × 10^3
Slc32a1 1.4 × 10^2
Gad2 2 × 10^3
Oxt 1.3 × 10^5
Pdyn 2.9 × 10^3
Avp 1.5 × 10^5
Npy1r 6 × 10^2
Sst 3.8 × 10^4
Trh 1.9 × 10^4
Crh 7 × 10^3
Ntng1 1.3 × 10^3
Reln 1.5 × 10^3
Penk 9.4 × 10^3

Fig. S1. Molecularly defined cell types in the PVH from single-cell RNA sequencing.
(**A**) Heat map of the expressions of 51 genes (48 differentially expressed marker-genes, as well as *Slc17a6*, *Slc32a1*, and *Sim1*) for 15 molecularly defined cell types by clustering single cell transcriptome profiles. Twelve marker-genes (blue bold) were chosen to combinatorially represent the major molecularly defined cell types and serve as marker-genes in the multiplexed FISH readout of CaRMA imaging. “Molecularly defined” cell type is defined here to mean transcriptional clusters from single-cell RNA-Seq. The relationships to more holistic definitions of cell type require extensive additional data and analysis, which is one purpose of CaRMA imaging. (**B**) Violin plots of the expression of 14 genes (11 PVH cell type differentially expressed genes, *Slc17a6*, *Slc32a1*, and *Sim1*) in these molecularly defined PVH cell types. *Vglut2* and *Vgat* are the common names for *Slc17a6* and *Slc32a1* used elsewhere in this study. Because clusters 5, 6 and 11 were primarily *Sim1*$^{-}$ neurons, and their primary marker genes were apparent outside the PVH based on data in the Allen Mouse Brain Atlas, we defined these 3 clusters as non-PVH neuron classes. The other 12 clusters were used as scRNA-seq PVH clusters in additional analyses.

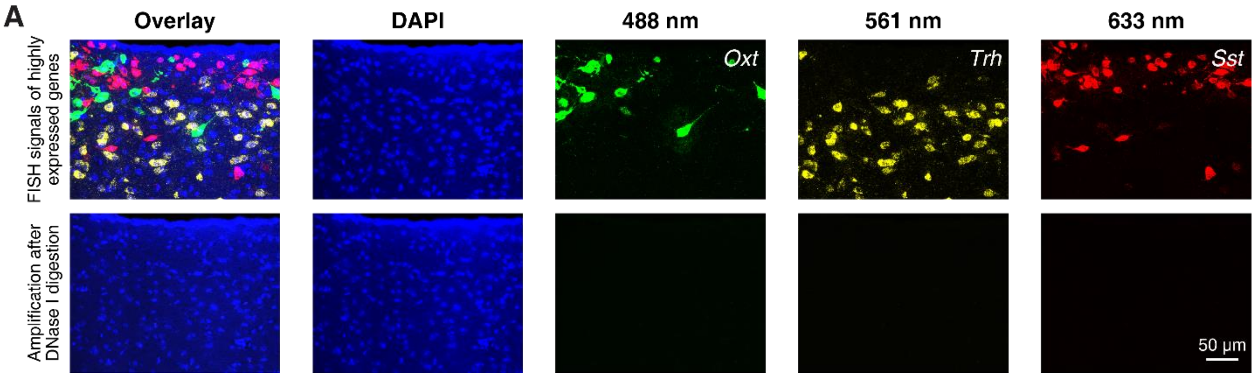

Fig. S2. Validation of fluorescently labeled probe stripping from one round of 3-plex FISH with DNase I.

(**A**) Top row: maximum intensity projection (MIP) images of the FISH signals of 3 highly expressed genes (*Oxt*, *Trh*, *Sst*) in the PVH, including an overlay of DAPI and FISH signals, as well as images from each acquisition channel. Bottom row: MIP images of the same field-of-view after DNase I digestion (see Methods) followed by RNAScope signal amplification steps without adding gene-specific probes. The images in both rows were acquired with the same microscope setting for FISH channels and displayed in the same intensity range. The absence of FISH signal after stripping, without gene-specific oligonucleotide probes, but with amplification demonstrates removal of the probes and the amplification oligonucleotides by DNase I.

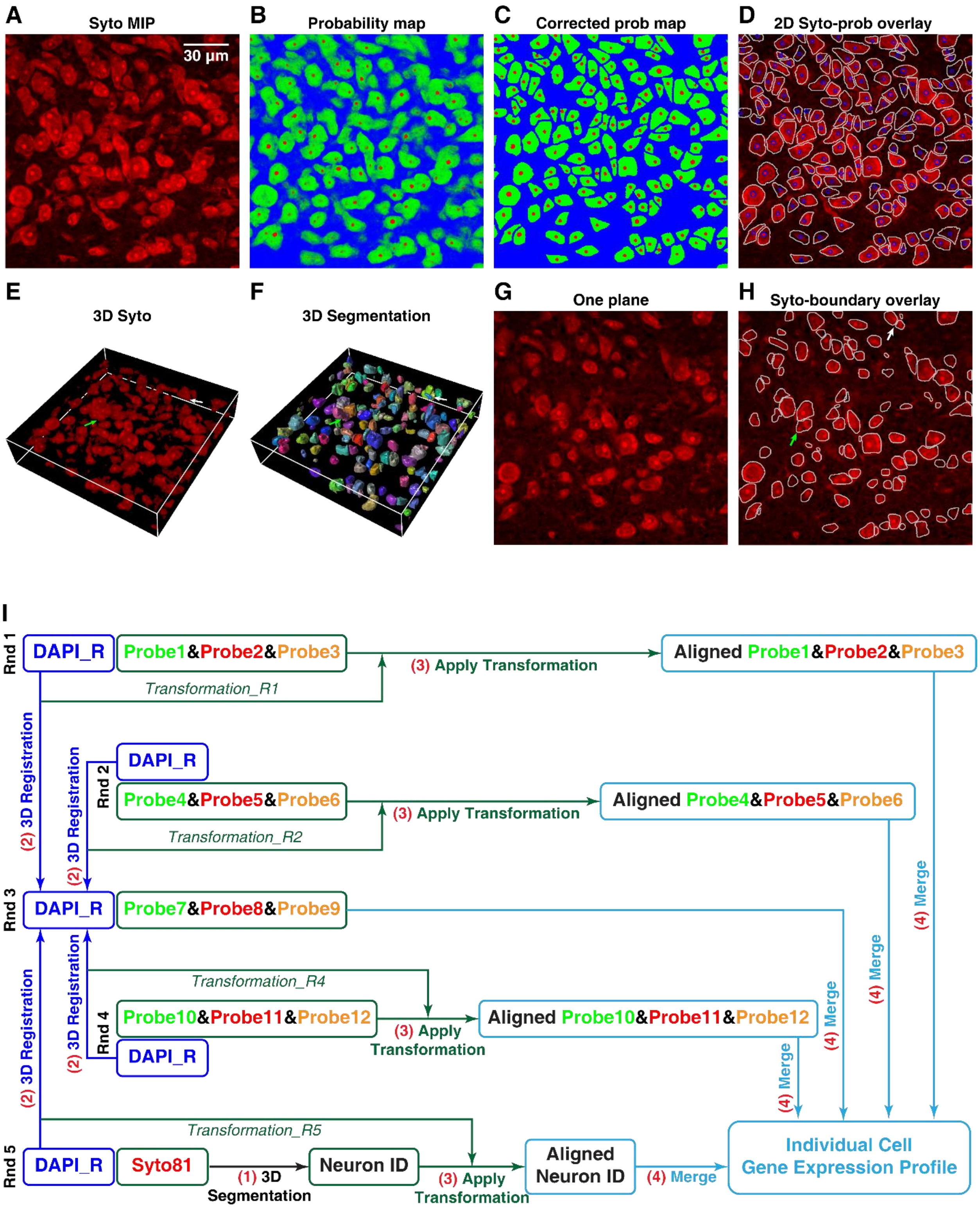

Fig. S3. Analysis pipeline of multiplexed FISH in FISH-only tissue

(**A-H**) Images demonstrating the procedure of 3D cell segmentation. Interactive machine learning image segmentation software (ilastik) was used to generate the 2D probability map (B) of background (blue), cell body (green) and intense fluorescent spot found in all nuclei after Syto

staining (red) from the maximum intensity projection (MIP) image of Syto staining (A). (**C**) The corrected probability map by thresholding and manually correcting the 2D probability map. (**D**) Overlay of MIP image of Syto and contours of corrected probability map of cell body (white) and nucleus bright spot (blue). (**E**) 3D view of Syto image stack. (**F**) 3D watershed segmentation of Syto image stack. Each colored volume represents one cell. The seeds for 3D watershed segmentation were mapped from the 2D nucleus bright spot in (C) by their depth indices of MIP. The 3D cell body foreground for segmentation were calculated from the intersection of binarized 3D Syto fluorescence using local adaptive Otsu's threshold and 3D cell body constraints, which were generated by extending the 2D cell bodies (red and green in (C)) to all depths (see Methods). This 3D cell body foreground was segmented into individual cells from the seeds using 3D watershed algorithm. (**G**) Syto image in one imaging plane. (**H**) Overlay of Syto and the segmented cell boundaries in one imaging plane. Green arrow: under-segmentation; White arrow: over-segmentation. (**I**) Diagram of analysis pipeline for generating gene expression profiles of individual cells from 4 rounds of 3-plexed FISH. Syto 81 staining in round 5 was used for 3D cell segmentation. DAPI_R: residual DAPI fluorescence after DNase I digestion and protease treatment, which was used for automated registration across imaging rounds.

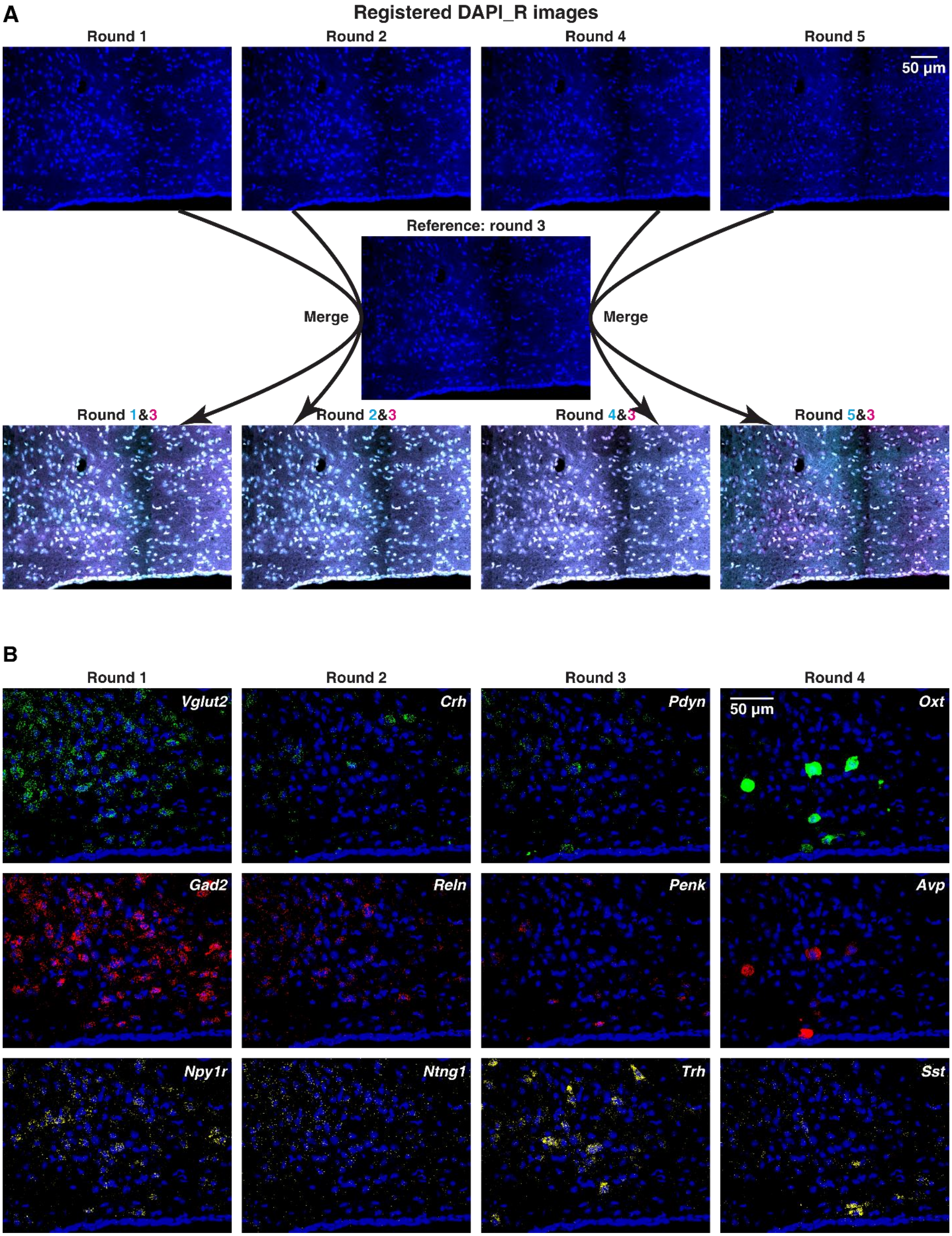

A
Registered DAPI_R images
Round 1
Round 2
Round 4
Round 5
50 μm
Reference: round 3
Merge
Merge
Round 1&3
Round 2&3
Round 4&3
Round 5&3
B
Round 1
Round 2
Round 3
Round 4
Vglut2
Crh
Pdyn
50 μm
Oxt
Gad2
Reln
Penk
Avp
Npy1r
Ntng1
Trh
Sst

Fig. S4. Examples of multi-round image registration and multiplexed FISH in FISH-only tissue

(**A**) Overlay of MIP of reference image (DAPI_R signal in round 3) and MIPs of 3D-registered images (DAPI_R signals) from other rounds. The overlay images were pseudocolored to illustrate alignment (text color corresponds to pseudocolor for that round). (**B**) MIP images of 4 rounds of 3-plexed FISH for the 12 marker-genes (the region with dashed white line in Fig. 2A).

A

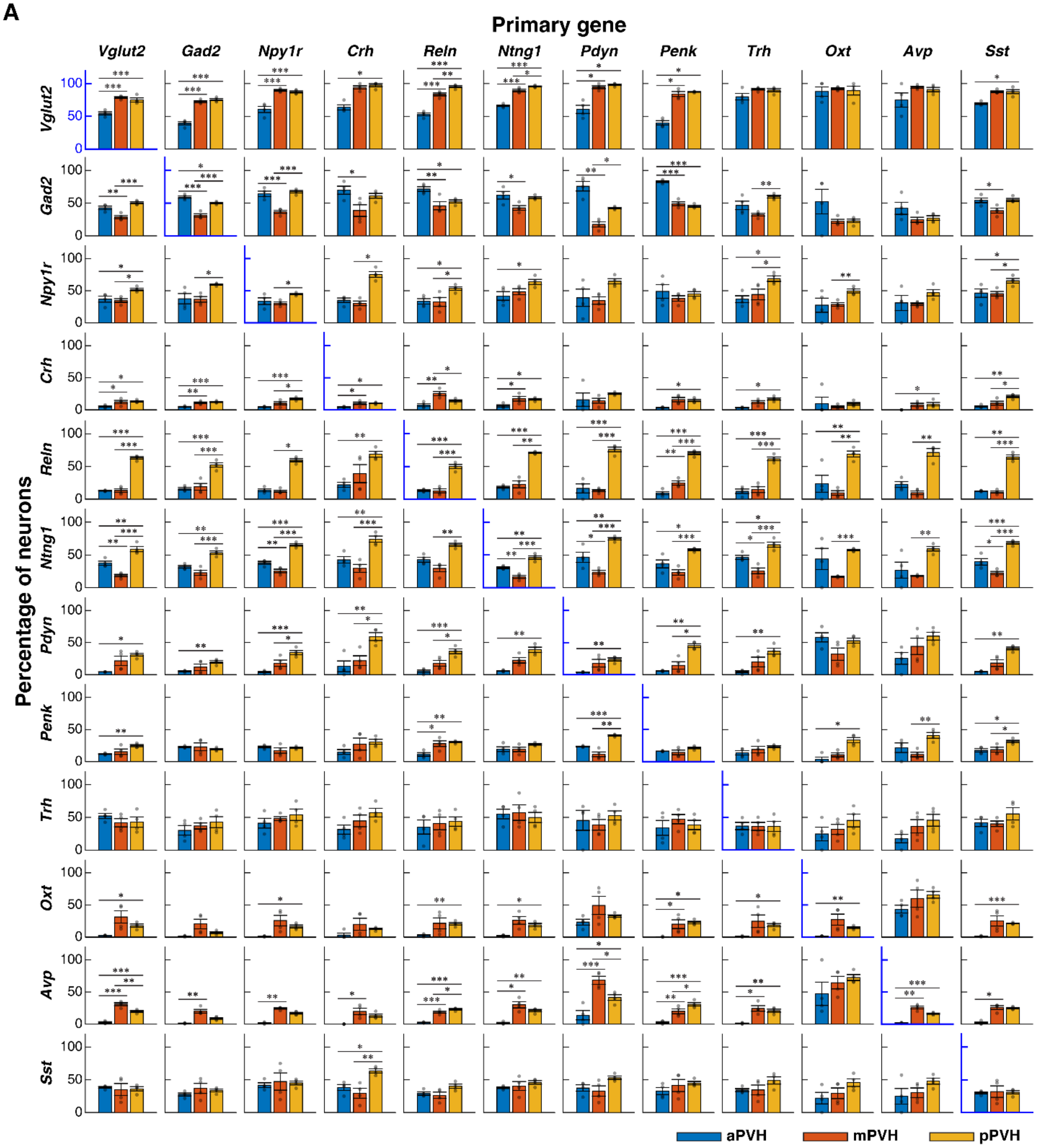

Fig. S5. Pairwise marker-gene co-expression.
(**A**) Black axes: percentage of the cells co-expressing primary gene (column) and secondary gene (row) in the cells expressing primary gene from the aPVH, mPVH and pPVH. Diagonal blue axes: same as Fig. 2B. Error bars: mean ± SEM. Gray circles: sample data. * $p < 0.05$, ** $p < 0.01$, *** $p < 0.001$. Statistics in Table S2.

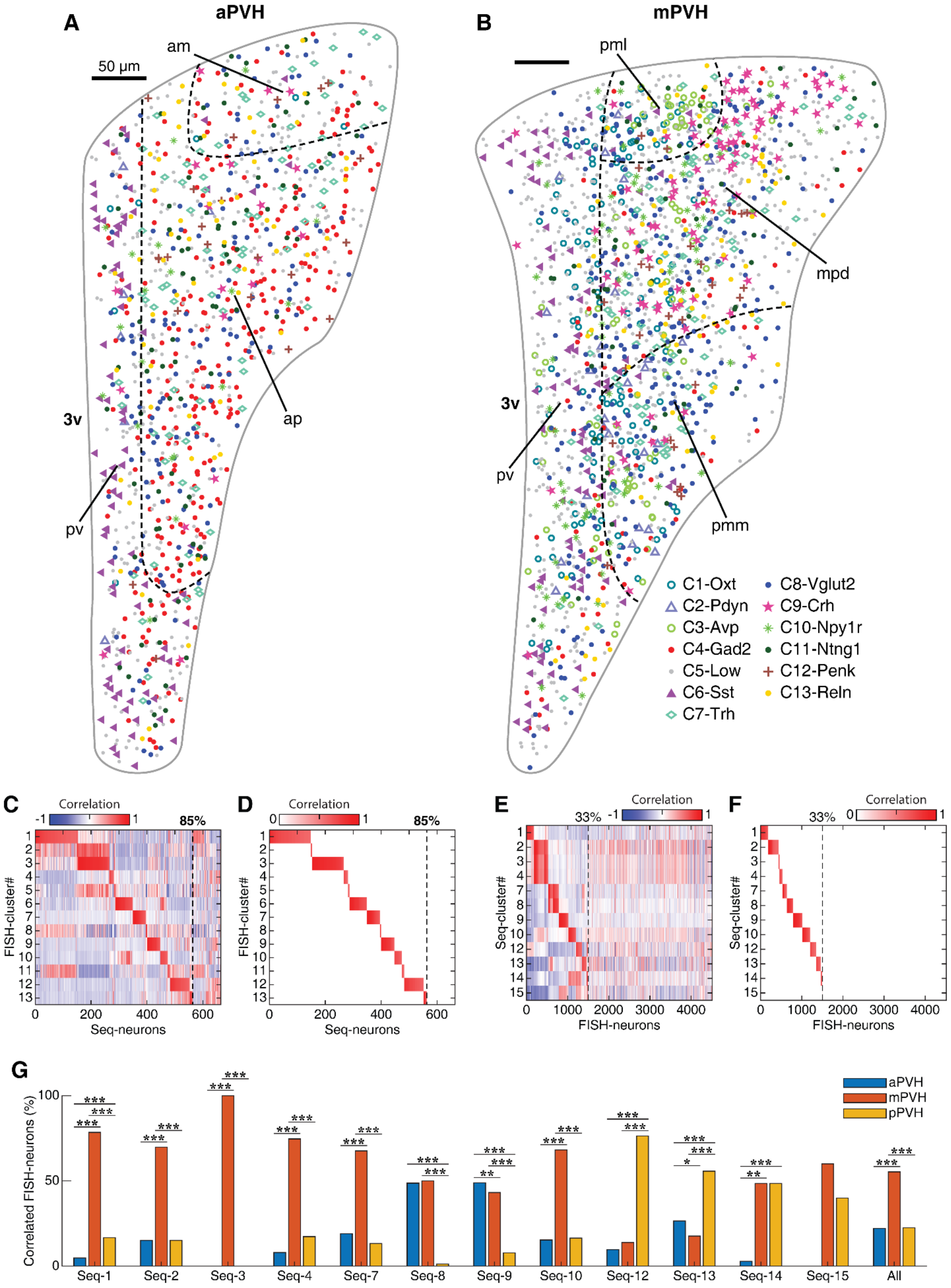
A
aPVH
am
50 μm
ap
3v
pv
B
mPVH
pml
mpd
3v
pv
pmm
C1-Oxt
C2-Pdyn
C3-Avp
C4-Gad2
C5-Low
C6-Sst
C7-Trh
C8-Vglut2
C9-Crh
C10-Npy1r
C11-Ntng1
C12-Penk
C13-Reln
C
Correlation
85%
FISH-cluster#
Seq-neurons
D
Correlation
85%
FISH-cluster#
Seq-neurons
E
Correlation
33%
Seq-cluster#
FISH-neurons
F
Correlation
33%
Seq-cluster#
FISH-neurons
G
Correlated FISH-neurons (%)
aPVH
mPVH
pPVH
Seq-1
Seq-2
Seq-3
Seq-4
Seq-7
Seq-8
Seq-9
Seq-10
Seq-12
Seq-13
Seq-14
Seq-15
All

Fig. S6. Spatial organization of PVH cell types and correspondence between scRNA-seq and 12-plex FISH.

(**A, B**) Spatial organization of 13 molecularly defined cell types (from clustering the FISH-only dataset) in aPVH (A) and mPVH (B). The cell type maps were generated by overlaying data from 4 samples. Each symbol represents one neuron. PVH subregions and their boundaries were identified in accordance to a previous study (*55*). am: anterior magnocellular part; ap: anterior parvicellular part; pmm: posterior magnocellular part, medial zone; mpd: medial parvicellular part, dorsal zone; pml: posterior magnocellular part, lateral zone. (**C**) The pairwise correlation coefficients between the expression profiles of FISH-clusters and individual Seq-neurons. Seq-neurons were ordered by their most significant correlation coefficient (See methods) within the FISH-clusters. (**D**) The correlation coefficients in (C) were filtered to show only the most significant correlation coefficient for each cell. (**E**) The pairwise correlation coefficients between the expression profiles of Seq-clusters and individual FISH-neurons. FISH-neurons were ordered in the same way as in (C). (**F**) The correlation coefficients in (E) were filtered as in (D). Dashed line in (C to F): the right boundary of significantly correlated neurons. (**G**) Anatomic distributions of the significantly correlated FISH-neurons in (F) for each Seq-cluster (see fig. S1) and all Seq-clusters. Note, there are only 5 FISH-neurons significantly correlated with Seq-15, thus they are not readily visualized in the Figure. The difference in the proportions of the correlated cell types from these datasets (C to G) is likely because close dissection of the PVH reduces inclusion of neurons from the PVH boundaries, and because hand-picking neurons, which has the benefit of being compatible with small sample volumes and avoiding most-nonneuronal cells, is biased to large cells. For example, magnocellular neurons (*Oxt* and *Avp* neurons) were much more abundant in the scRNA-Seq dataset (Seq-clusters 1-4 in scRNA-seq: 47%; FISH-clusters 1-3 in multiplexed FISH: 7%) and were also overrepresented in the mPVH. * $p < 0.05$, ** $p < 0.01$, *** $p < 0.001$. Statistics in Table S2.

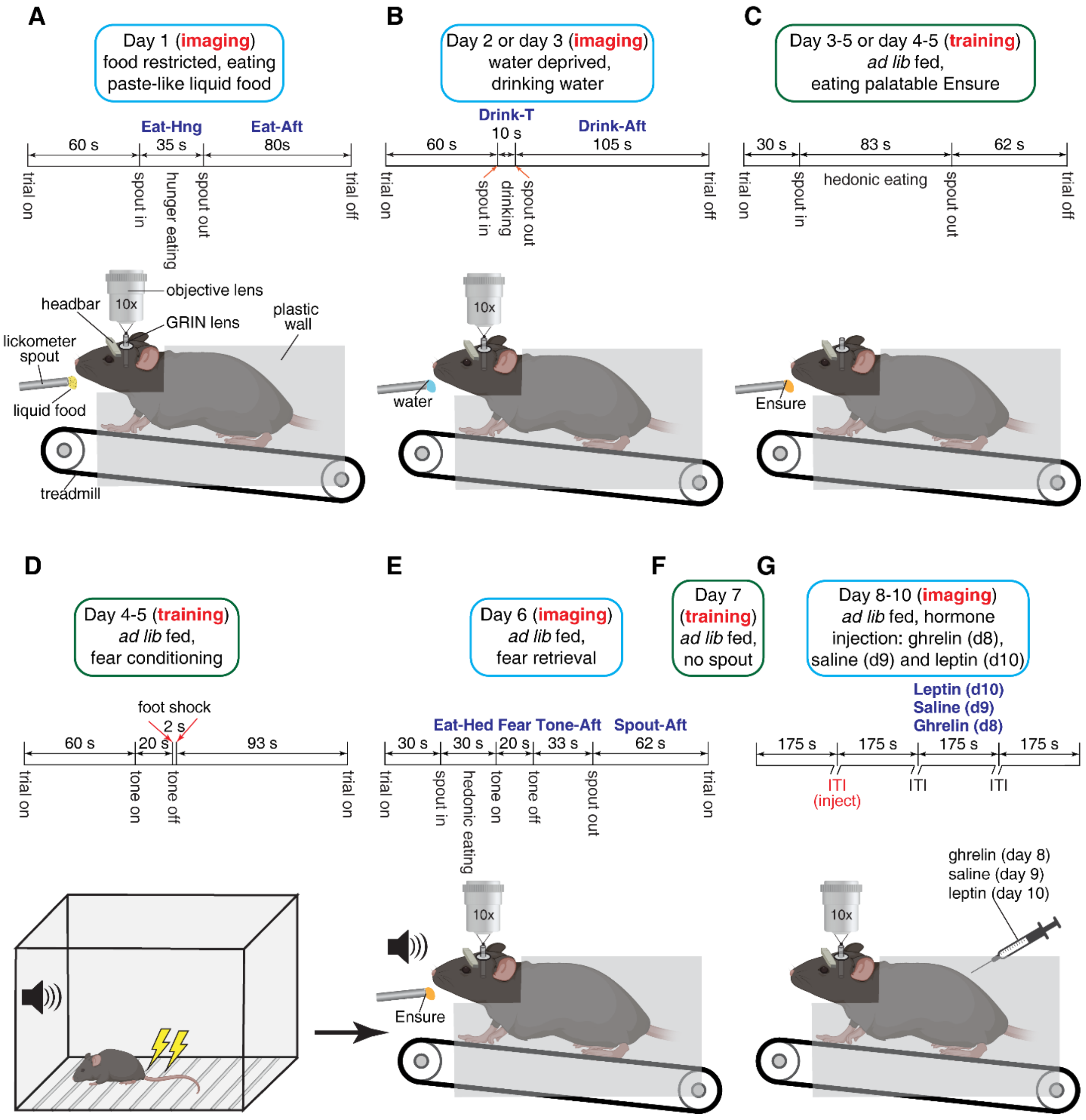

Fig. S7. Schematics of experimental sequence for calcium imaging and animal training across multiple behaviors.

(**A-G**) Schematics of experimental setups, trial structures, physiological conditions. Order of experiments is shown along with abbreviations for 11 behavioral states (blue text). (**A**) Imaging in food-restricted mouse eating moderate palatability liquid food in hunger state. (**B**) Imaging water-deprived mouse drinking water in thirst state. (**C**) *Ad lib* fed mouse trained to eat palatable Ensure. (**D**) Auditory fear conditioning (tone paired with foot-shock). (**E**) Imaging *ad lib* fed mouse that was first hedonic eating, then heard a tone (fear-retrieval). The tone stopped while the food spout was still present and lick suspension during eating indicated the fear emotional state. After a delay, the spout was withdrawn. (**F**) *Ad lib* fed mouse was habituated to head fixation with no spout for food and water delivery. (**G**) Injection of ghrelin, saline and leptin were on

successive days. ITI in (G): inter-trial interval (2 min). Inter-trial intervals in (A-E) were randomly set between 90 to 180 s.

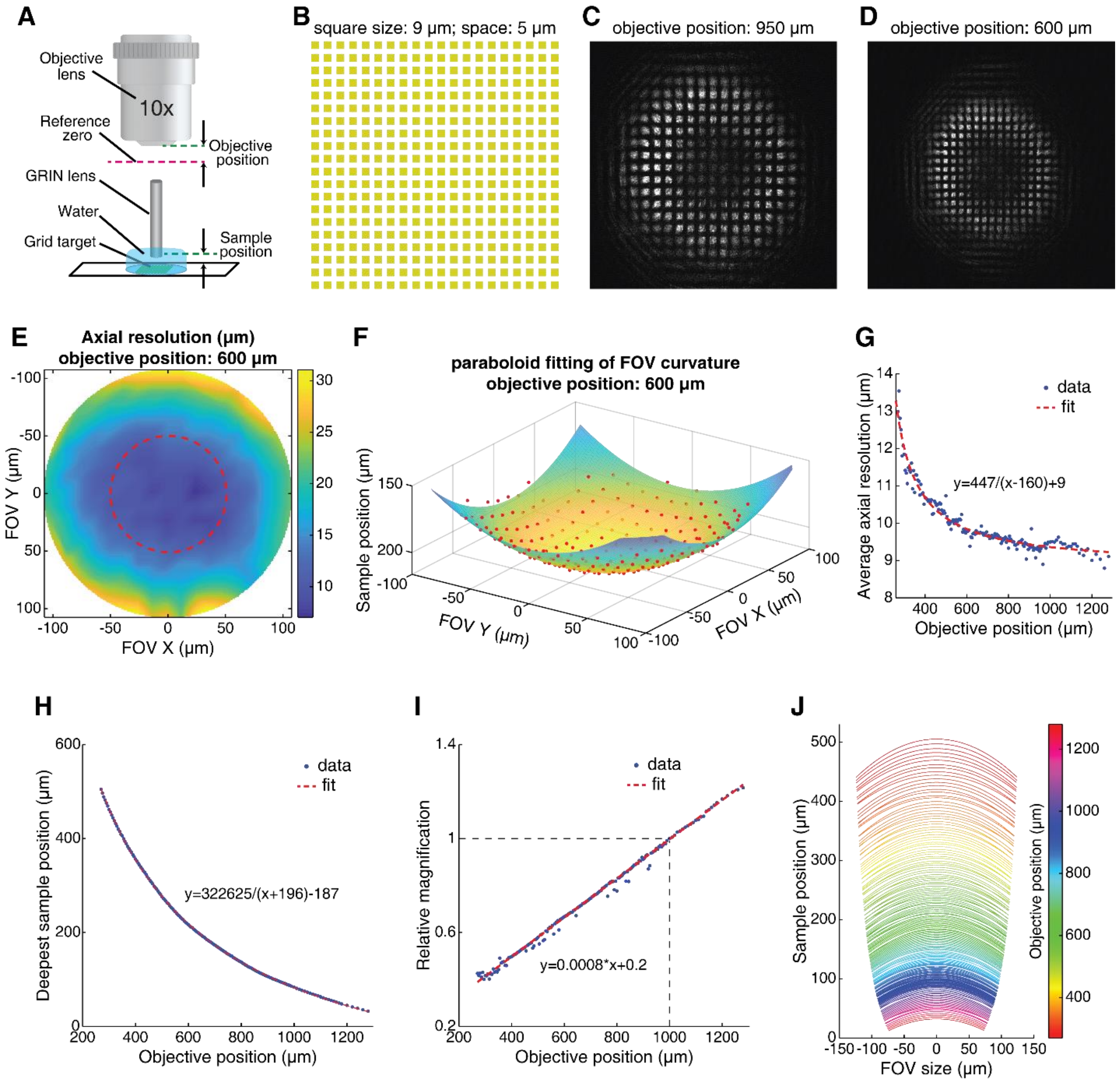

Fig. S8. Optical properties of 2P-GRIN lens imaging system.
(**A**) Schematic configuration of the calibration setup. (**B**) Two photon excitable grid target. (**C-D**) Example images showing the field curvature and depth-dependent magnification. (**E**) Axial resolution map of an example FOV. (**F**) Field curvature of an example FOV. Red dots: data; surface: paraboloid fit. (**G**) Relationship between mean axial resolution and objective lens position. The mean axial resolution was the mean value of axial resolutions within the center 100 µm diameter circle, such as the red dashed circle in (E). (**H**) Relationship between the deepest sample position (bottom of curved FOV) and objective lens position. (**I**) Relationship between magnification and objective lens position. Magnification is set to 1 when objective lens is at 1000 µm. (**J**) Relationship between imaging surface, including the field curvature, sample position, and objective lens position.

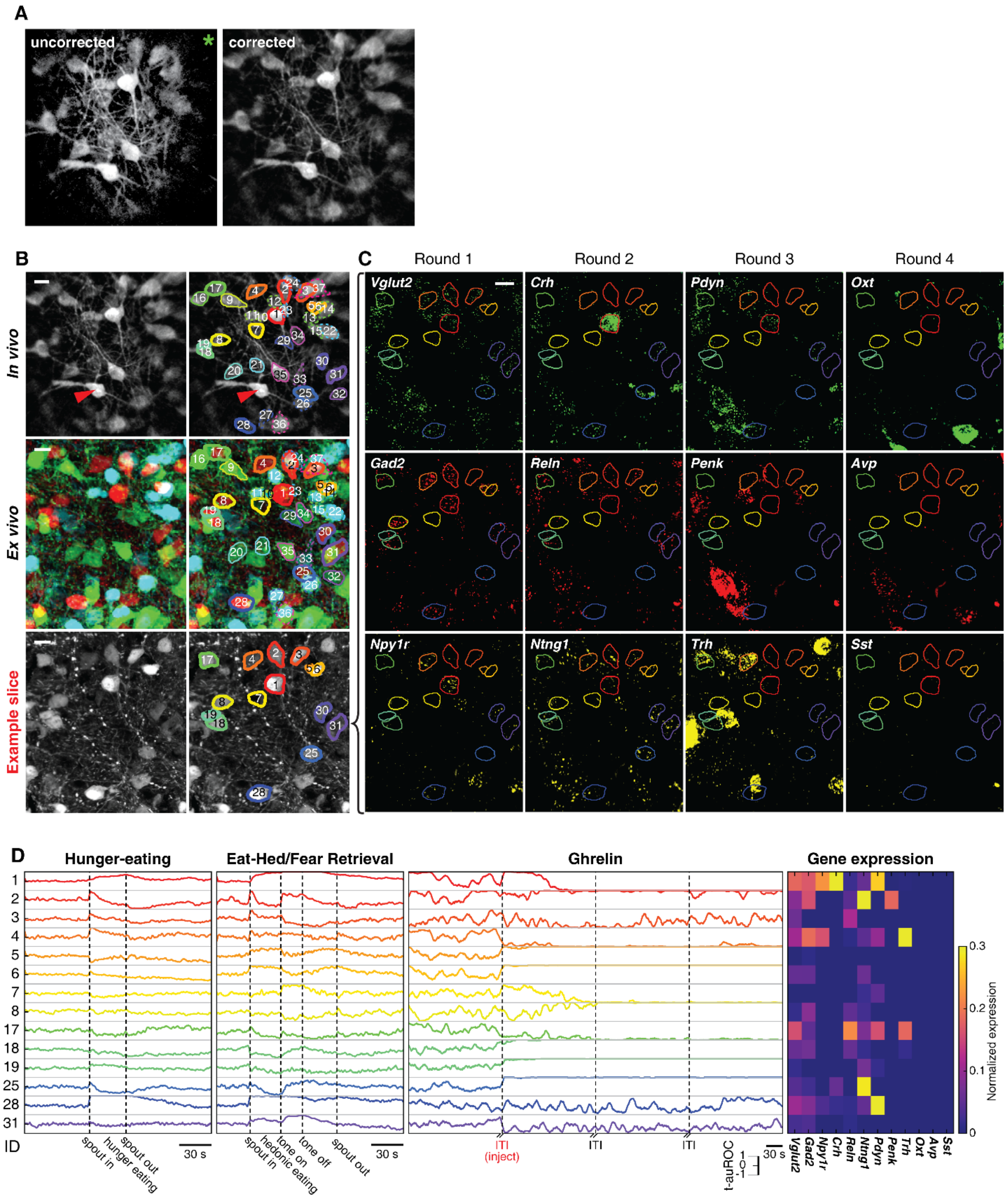

Fig. S9. Second example of ex vivo to in vivo registration for CaRMA imaging.
(**A**) Computational correction of optical aberrations from *in vivo* imaging. Green *: imaging plane with the same marker in Fig. 3B. (**B**) Example neurons showing the *ex vivo* registration to a substack of the *in vivo* image volume. *Ex vivo* image is overlay of z-projected confocal stacks from 3 consecutive 14 µm brain slices (pseudocolor: green, top slice; red, middle slice; cyan,

bottom slice). Thin contours: neurons in the top slice; thick contours: neurons from the middle slice; dashed contours: neurons in the bottom slice. Red arrowhead: Neuron which could not be found in confocal images (lost during sectioning). (**C**) Four rounds of 3-plex FISH signals of the neurons in the middle slice from (B). (**D**) Responses of the neurons in (C) across multiple behavioral states and their 12-plex gene expression profiles. ID: neuron identification number; ITI: inter-trial interval (2 min). Scale bars in (B) and (C): 15 μm.

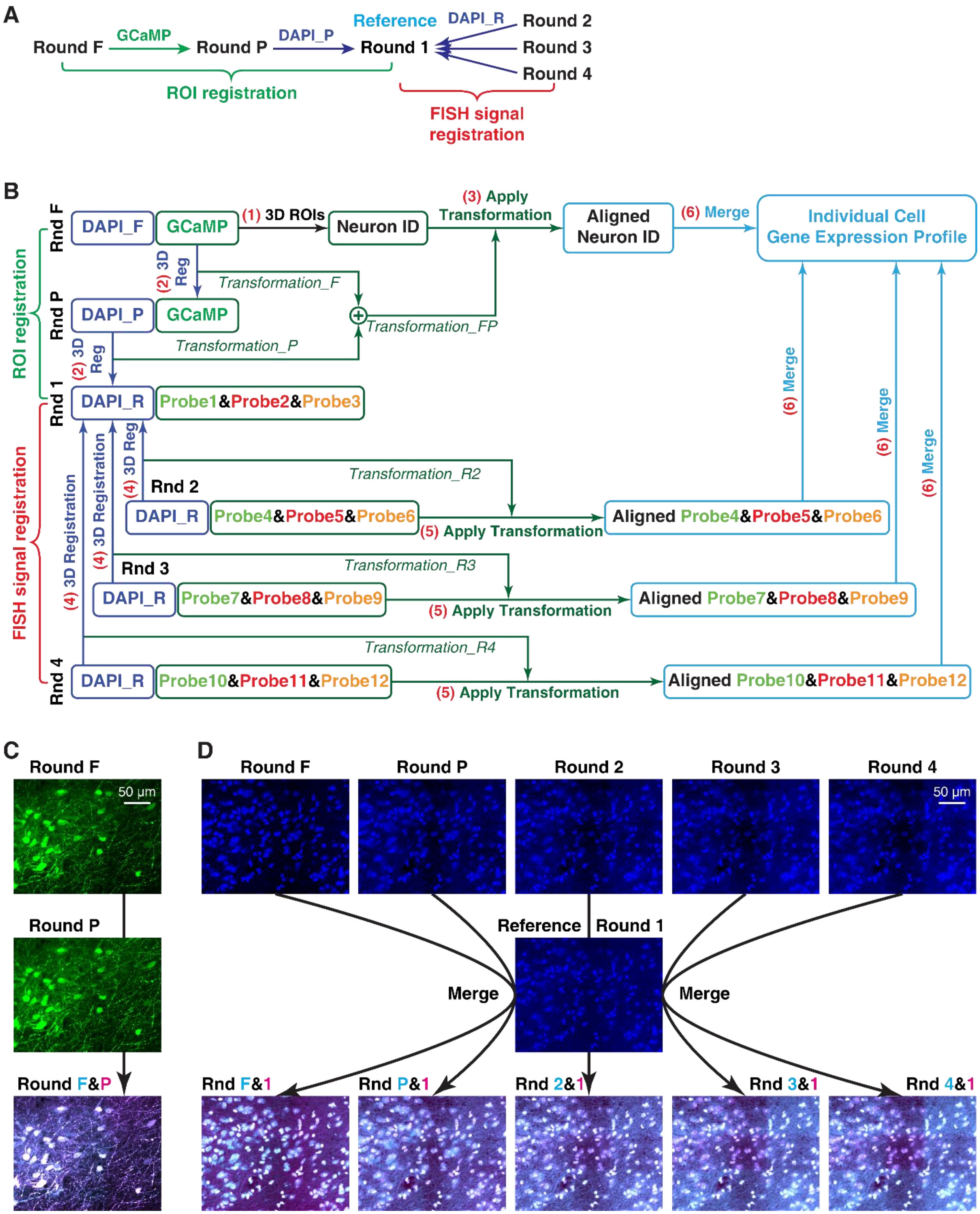
A
Round F
GCaMP
Round P
DAPI_P
Reference
Round 1
DAPI_R
Round 2
Round 3
Round 4
ROI registration
FISH signal registration
B
Rnd F
DAPI_F
GCaMP
(1) 3D ROIs
Neuron ID
(3) Apply Transformation
Aligned Neuron ID
(6) Merge
Individual Cell Gene Expression Profile
(2) 3D Reg
Transformation_F
Rnd P
DAPI_P
GCaMP
Transformation_FP
Transformation_P
ROI registration
Rnd 1
DAPI_R
Probe1&Probe2&Probe3
FISH signal registration
(4) 3D Reg
(4) 3D Registration
Rnd 2
DAPI_R
Probe4&Probe5&Probe6
Transformation_R2
(5) Apply Transformation
Aligned Probe4&Probe5&Probe6
Rnd 3
DAPI_R
Probe7&Probe8&Probe9
Transformation_R3
Aligned Probe7&Probe8&Probe9
Rnd 4
DAPI_R
Probe10&Probe11&Probe12
Transformation_R4
Aligned Probe10&Probe11&Probe12
C
Round F
50 μm
Round P
Round F&P
D
Round F
Round P
Round 2
Round 3
Round 4
50 μm
Reference
Round 1
Merge
Rnd F&1
Rnd P&1
Rnd 2&1
Rnd 3&1
Rnd 4&1

Fig. S10. Image registration pipeline for multi-round multiplexed FISH after CaRMA imaging.
(**A**) Overview of the sequence of multi-round registration steps. Arrows indicate the registration direction and the signals used for the registrations. The ROI registration includes 2 steps (details as shown in (B)) (**B**) Detailed flowchart of registration pipeline for generating gene expression profiles of individual cells in CaRMA imaging from 4 rounds of 3-plexed FISH. Transformation_FP: the concatenation of transformation_F and transformation_P. DAPI_F: DAPI full, DAPI fluorescence without DNase I digestion and protease treatment; DAPI_P: DAPI partial, DAPI fluorescence with DNase I digestion but without protease treatment; DAPI_R: residual DAPI fluorescence after DNase I digestion and protease treatment (see Methods). (**C**) Overlay of maximum intensity projections (MIPs) of reference GCaMP image (Round P) and 3D-registered GCaMP image (Round F) from the same region as (D). (**D**) Overlay of MIP of reference DAPI image (DAPI_R signal in round 1) and MIPs of 3D-registered DAPI images from other rounds (DAPI_F signal from round F, DAPI_P signal from round P and DAPI_R signals from round 2-4). The overlay images were pseudocolored to illustrate alignment (text color corresponds to pseudocolor for that round). Overlap of DAPI_F and DAPI_R in round 1 signals validated the 2-step ROI registration. Notably, after DNase I digestion, nuclear DAPI staining became fragmented and nonspecific fiber-like DAPI staining appeared in background. These staining features provided reliable fiducial landmarks for the 3D registration.

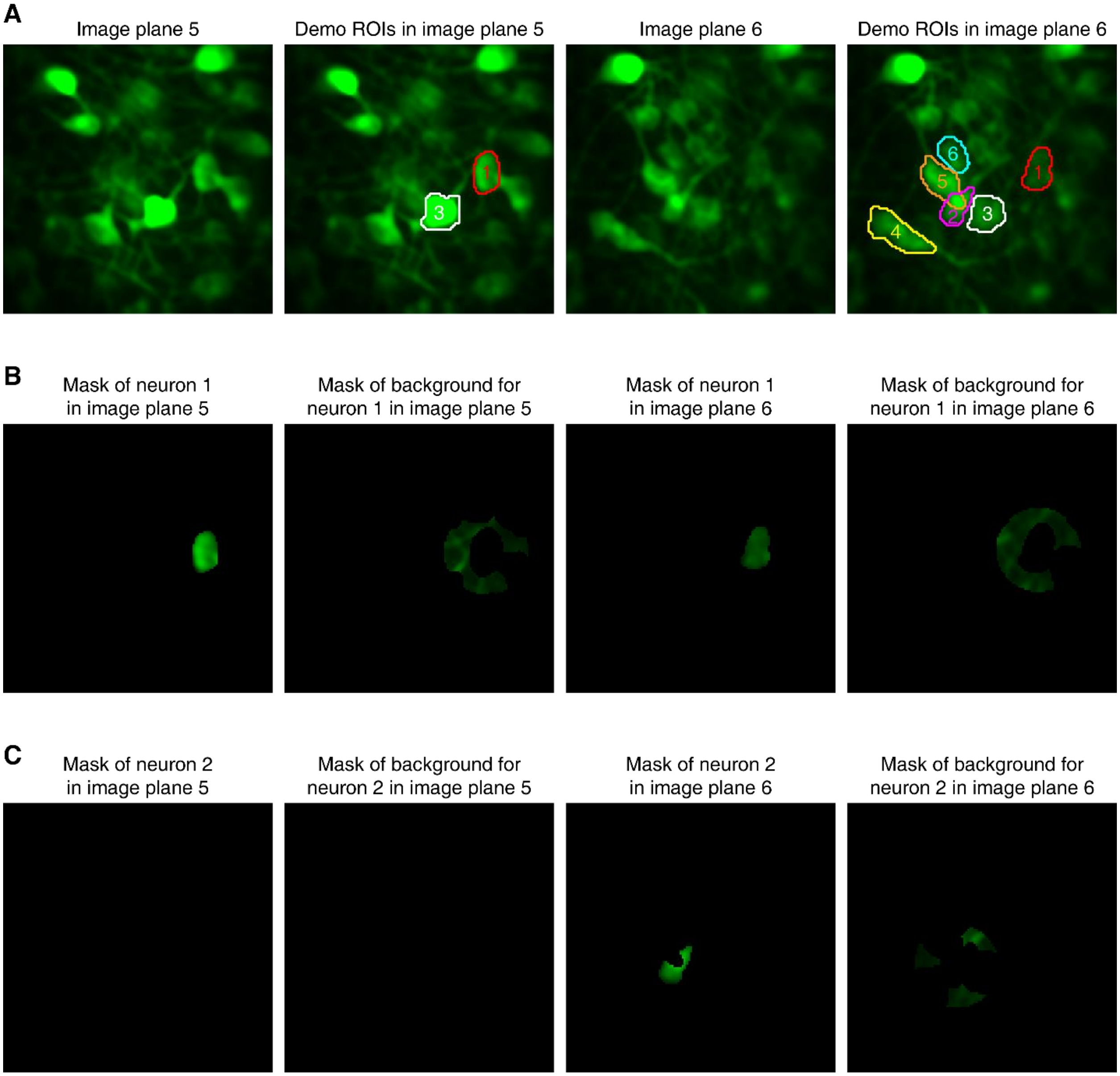

Fig. S11. Extraction of neuronal calcium signals from two-photon-GRIN-lens volumetric imaging.
(**A**) Example neurons in 2 consecutive imaging planes to show the image characteristics of two-photon-GRIN-lens volumetric imaging. Because of volumetric scanning and elongated axial resolution (fig. S8, E and G), some neurons were imaged across imaging planes (Neuron 1 and 3) or spatially overlapped (Neuron 2 and 5). (**B**) Masks of example neuron 1 and its background (see Methods) in these 2 imaging planes. (**C**) Masks of example neuron 2 and its background in these 2 imaging planes. The overlapped region between neuron 2 and 5 was excluded from the mask of neuron 2.

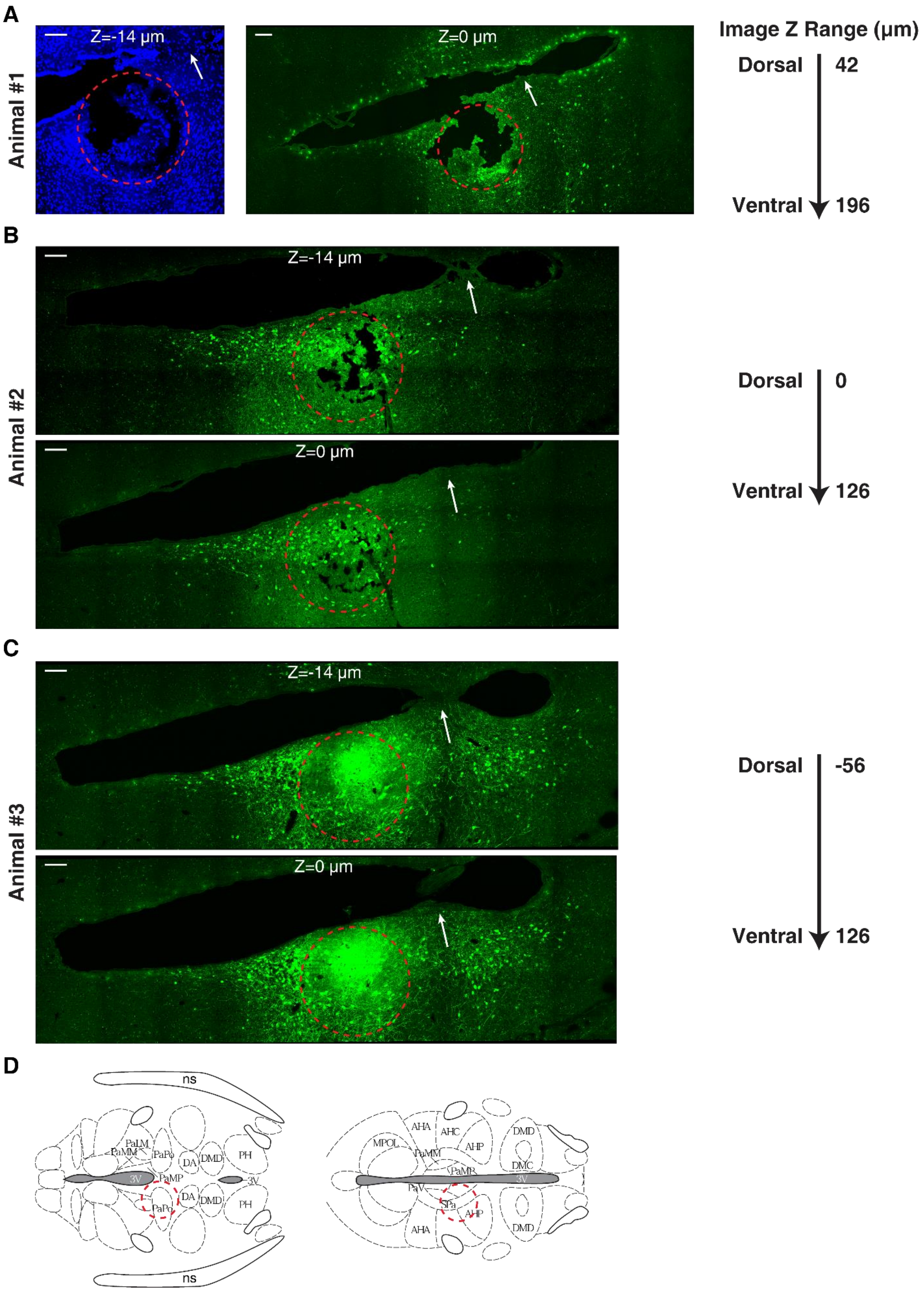
A
Animal #1
Z=-14 µm
Z=0 µm
Image Z Range (µm)
Dorsal
42
Ventral
196
B
Animal #2
Z=-14 µm
Z=0 µm
Dorsal
0
Ventral
126
C
Animal #3
Z=-14 µm
Z=0 µm
Dorsal
-56
Ventral
126
D
ns
PaLM
PaMM
PaPo
DA
DMD
PH
3V
PaMP
AHA
AHC
AHP
MPOL
DMC
PaV
SPa

Fig. S12. Anatomic locations of CaRMA imaging regions in 3 animals.
(**A-C**) Anatomic identification of the PVH was used to define dorsal/ventral position in horizontal brain slices and the depth ranges of our CaRMA imaging experiments. Left confocal images show GRIN lens target regions at depth -14 µm and 0 µm slices. The white arrows point to the merge of 3rd ventricle from 2 holes into 1 hole. The reference depth 0 µm for our CaRMA imaging analysis was the z-position of the first ventral slice with one merged 3rd ventricle. The right black arrows indicate the depth ranges (in µm) of CaRMA imaging volumes relative to this reference depth. Scale bar: 100 µm. (**D**) CaRMA imaging regions on mouse horizontal brain atlas. Atlas images are reproduced from (*68*) (left: -4.72 mm; right: -4.88 mm; distance from skull surface at Bregma. The 0 µm reference depth in our CaRMA imaging analysis is between these 2 images). The red dashed circles in (A-D) indicate the regions under GRIN lens for each mouse - see depth ranges in (A-C) for dorsal/ventral extent of image volume in each subject.

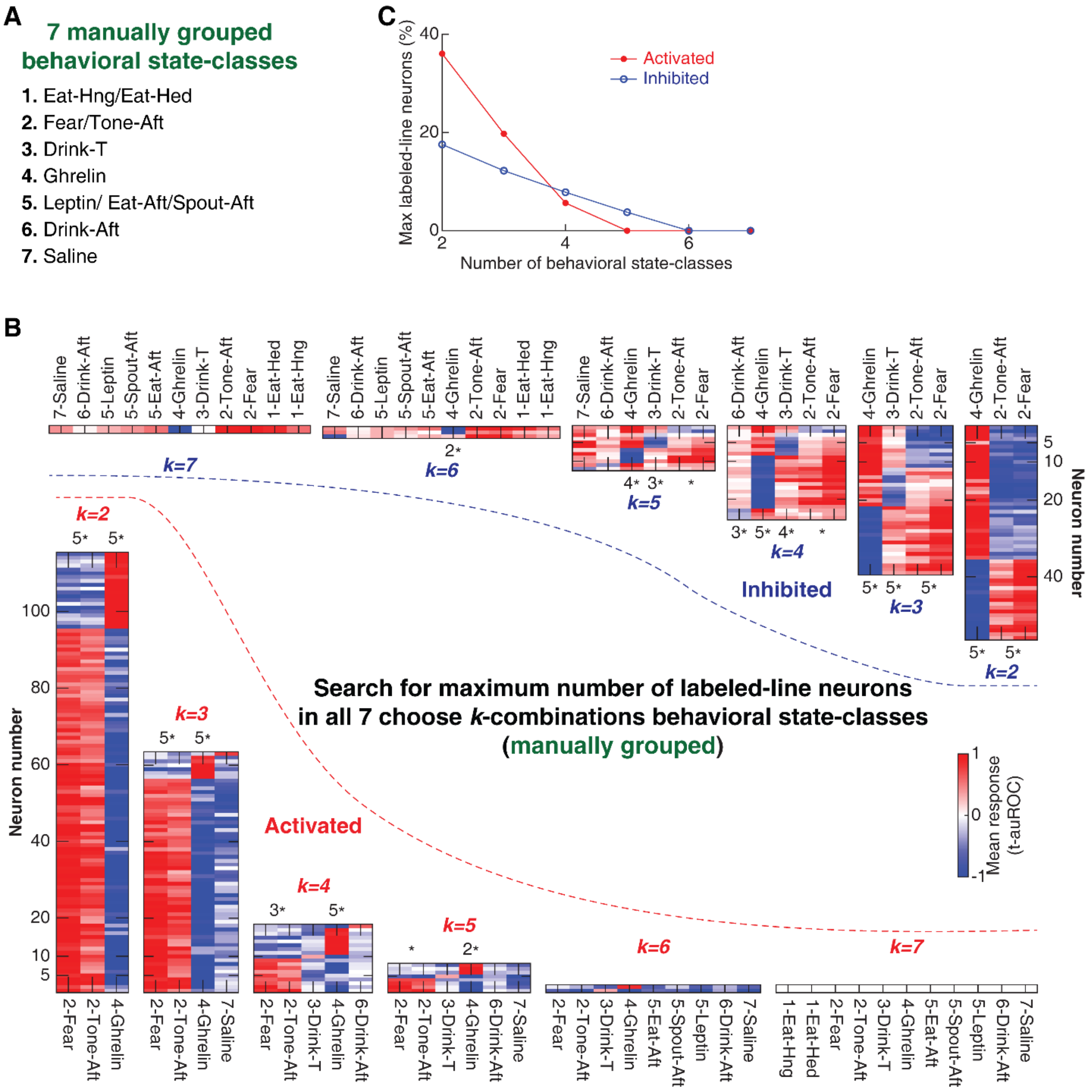

Fig. S13. Search for labeled-line neurons encoding 7 manually grouped behavioral state-classes.
(**A**) Seven manually grouped behavioral state-classes based on subjective thoughts about behavioral similarity. (**B**) Mean response maps of the maximum number of labeled-line neurons in all 7 choose ***k***-combinations of behavioral state-classes. Bottom left: activated labeled-line neuron sets. Top right: inhibited labeled-line neuron sets. Fisher's exact test was used to evaluate whether neurons are significantly specialized for a behavioral state-class. ***k*** = 2 to 7. (**C**) Number of labeled-line neurons depends on the number of behavioral state-classes. ***k*** = 2 to 7. *: $p < 0.05$; 2*: $p < 0.01$; 3*: $p <$ 1e-3; 4*: $p <$ 1e-4; 5*: $p <$ 1e-5.

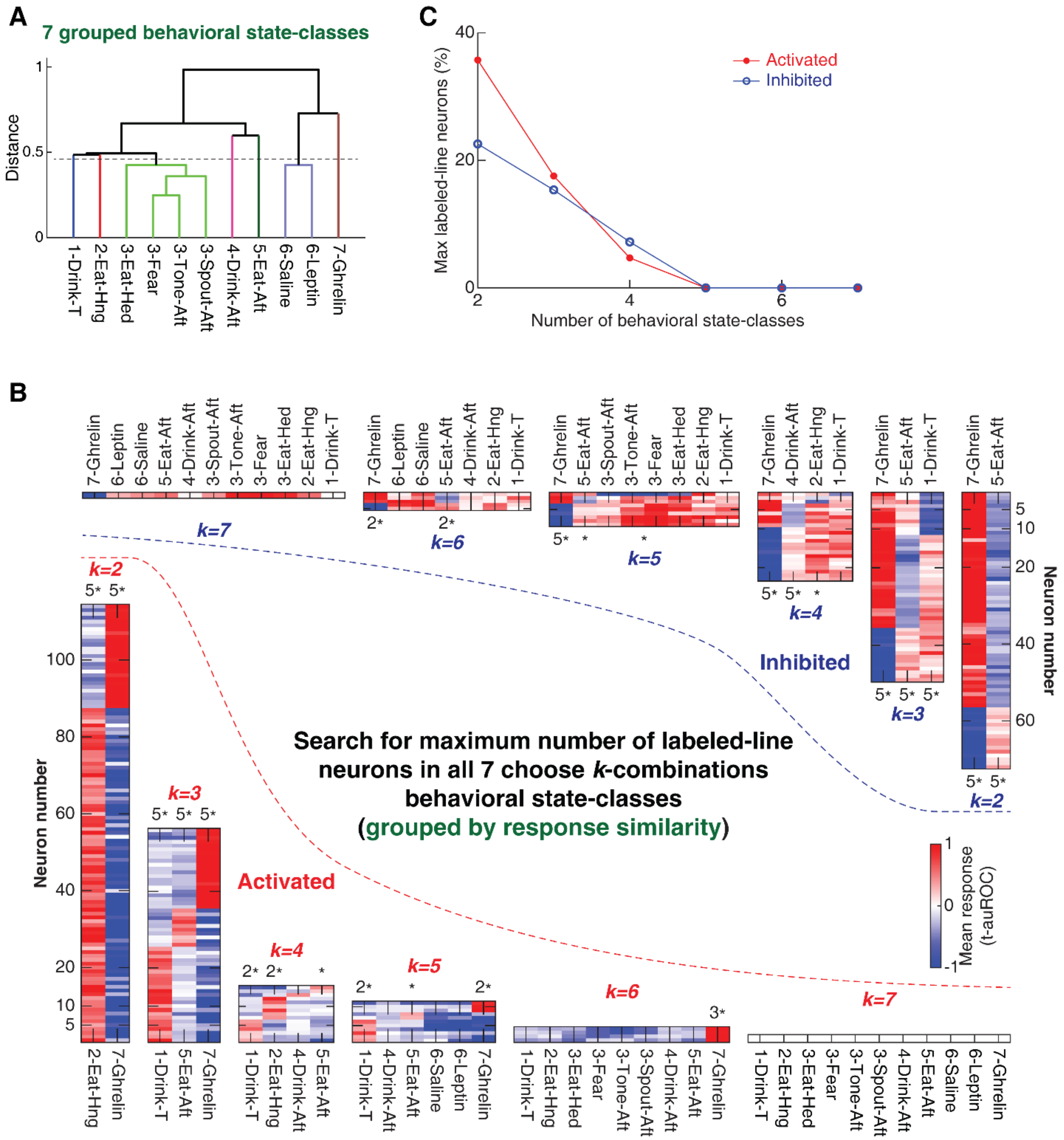

Fig. S14. Search for labeled-line neurons encoding 7 behavioral state-classes grouped by response similarity.
(**A**) Seven behavioral state-classes grouped by their pPVH ensemble response similarity. Dashed line: grouping threshold (threshold 7 in Fig. 4A). (**B**) Mean response maps of the maximum number of labeled-line neurons in all 7 choose ***k***-combinations behavioral state-classes. Bottom left: activated labeled-line neuron sets. Top right: inhibited labeled-line neuron sets. Fisher's exact test was used to evaluate whether neurons are significantly specialized for a behavioral state-class. ***k*** = 2 to 7. (C) Number of labeled-line neurons depends on the number of behavioral state-classes. ***k*** = 2 to 7. *: $p < 0.05$; 2*: $p < 0.01$; 3*: $p < 1e-3$; 4*: $p < 1e-4$; 5*: $p < 1e-5$.

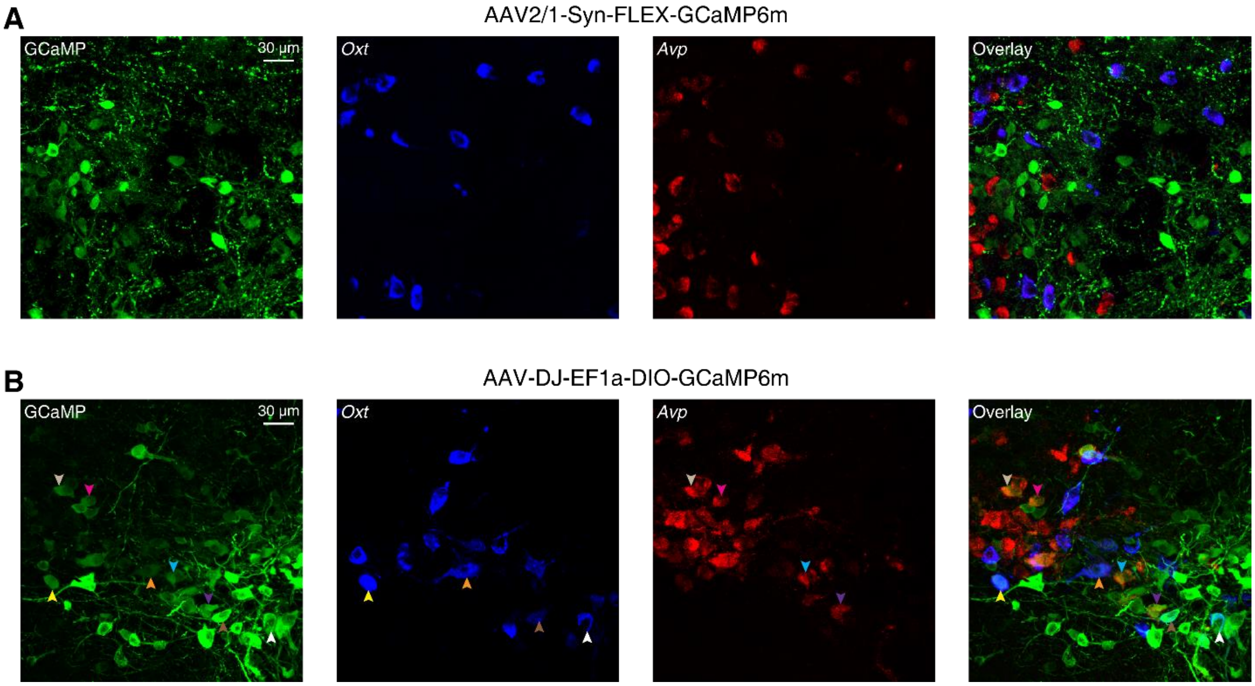

Fig. S15. Viral tropism of the AAV2/1.
(**A**) Neither *Oxt*$^+$ or *Avp*$^+$ neurons express GCaMP by injecting AAV2/1-Syn-FLEX-GCaMP6m into PVH of S*im1-Cre* mouse. Other examples are in Fig. 3E and fig. S9C. (**B**) GCaMP expressions in many *Oxt*$^+$ and *Avp*$^+$ neurons were observed after injecting AAV-DJ-EF1a-DIO-GCaMP6m into PVH of S*im1-Cre* mouse. Colored arrowheads mark the corresponding neurons.

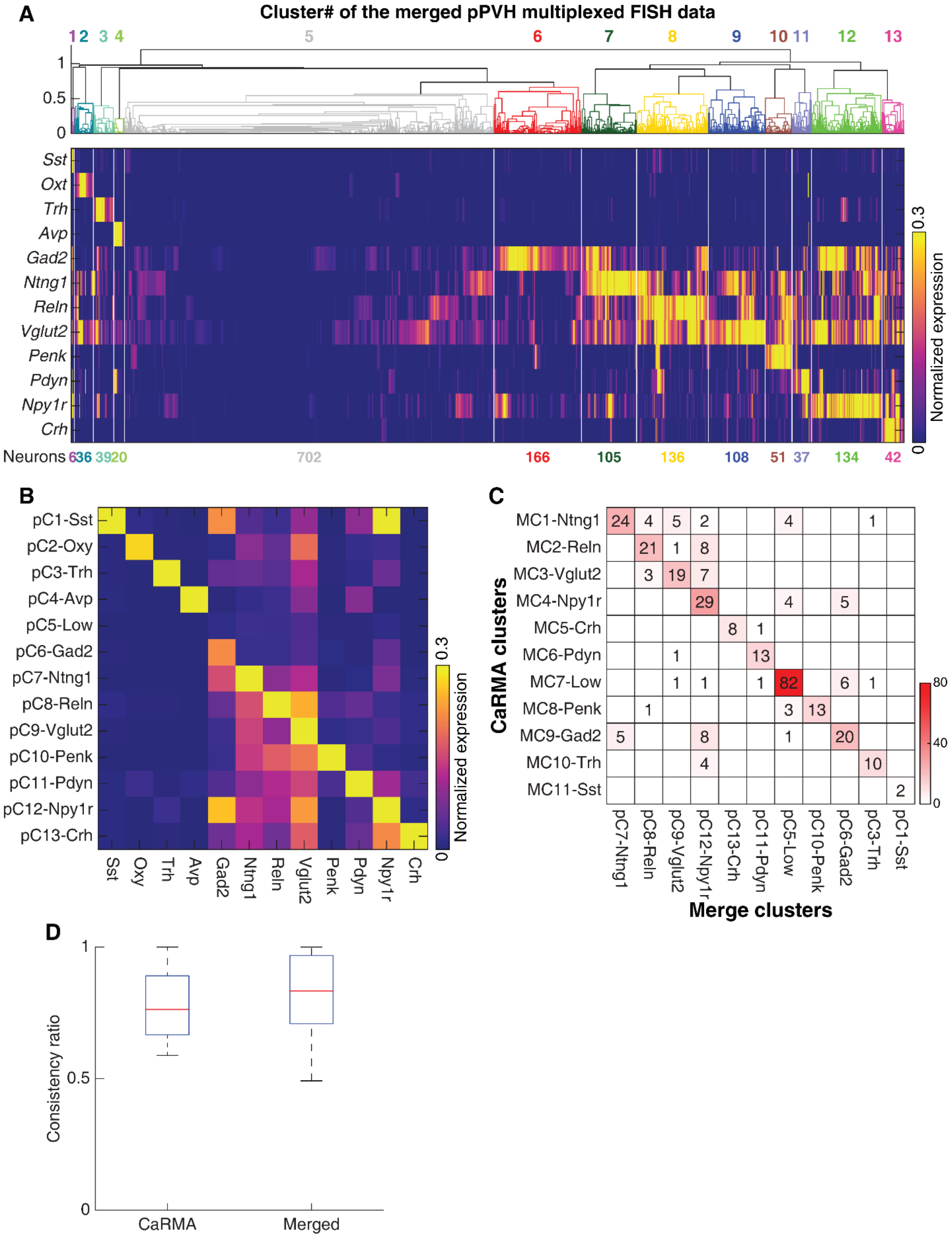
A
Cluster# of the merged pPVH multiplexed FISH data
1 2 3 4 5 6 7 8 9 10 11 12 13
1
0.5
0
Sst
Oxt
Trh
Avp
Gad2
Ntng1
Reln
Vglut2
Penk
Pdyn
Npy1r
Crh
Neurons 6 36 39 20 702 166 105 136 108 51 37 134 42
0.3
0
Normalized expression
B
pC1-Sst
pC2-Oxy
pC3-Trh
pC4-Avp
pC5-Low
pC6-Gad2
pC7-Ntng1
pC8-Reln
pC9-Vglut2
pC10-Penk
pC11-Pdyn
pC12-Npy1r
pC13-Crh
Sst Oxy Trh Avp Gad2 Ntng1 Reln Vglut2 Penk Pdyn Npy1r Crh
0.3
0
Normalized expression
C
CaRMA clusters
MC1-Ntng1 24 4 5 2 4 1
MC2-Reln 21 1 8
MC3-Vglut2 3 19 7
MC4-Npy1r 29 4 5
MC5-Crh 8 1
MC6-Pdyn 1 13
MC7-Low 1 1 1 82 6 1
MC8-Penk 1 3 13
MC9-Gad2 5 8 1 20
MC10-Trh 4 10
MC11-Sst 2
pC7-Ntng1 pC8-Reln pC9-Vglut2 pC12-Npy1r pC13-Crh pC11-Pdyn pC5-Low pC10-Penk pC6-Gad2 pC3-Trh pC1-Sst
Merge clusters
80
40
0
D
Consistency ratio
1
0.5
0
CaRMA
Merged

Fig. S16. Molecular clustering of the neurons from CaRMA imaging is consistent with PVH clusters from FISH in FISH-only tissue.
(**A**) Gene expression profiles of individual cells in the merged pPVH 12-plex FISH dataset (pPVH FISH-only and CaRMA datasets). Hierarchical clustering of these neurons based on their gene expression profiles resulted in 13 molecular clusters. (**B**) Mean expression pattern of the marker genes in the 13 molecular clusters from hierarchical clustering in (A). Molecular clusters are denoted as pC*i*-*xxx*, where *i* is the cluster number in (A) and *xxx* is a high-expressed gene or "Low" (low expression for all probed genes). (**C**) Consistency matrix between the molecular clusters determined with the merged dataset (merge clusters) and the clusters determined with CaRMA dataset (CaRMA clusters in Fig. 5B) for individual CaRMA neurons. (**D**) Consistency ratios of CaRMA clusters and merge clusters. Consistency ratios of CaRMA clusters are the diagonal values element-wise divided by the sum of values along the row in the consistency matrix in (C). Consistency ratios of clusters from the merged datasets are the diagonal values element-wise divided by the sum of values along the column in the consistency matrix in (C).

A

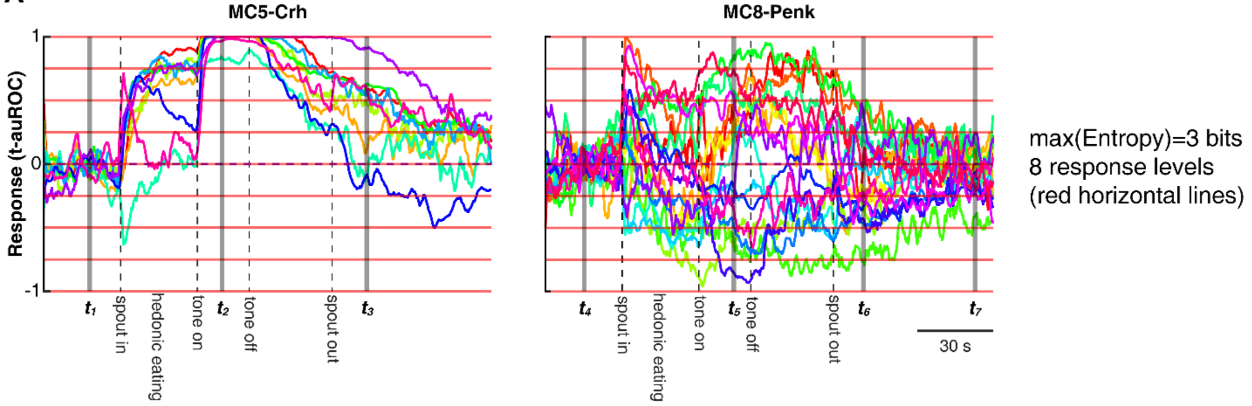

B

	Response distribution	Entropy (S) $-\sum_{i=1}^{8} p_i \log_2 p_i$	Purity (P) $\frac{1-2^{(S-3)}}{1-2^{-3}}$	Mean response (R_m)	Consistent response (R_m*P)	Notes
t_1	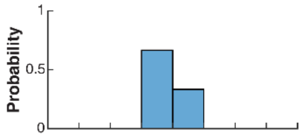	0.92	0.87	-0.02	-0.018	low mean response (baseline) high purity
t_2	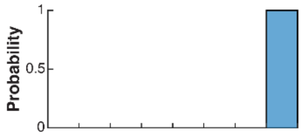	0	1	0.98	0.98	high mean response high purity neurons respond similarly
t_3	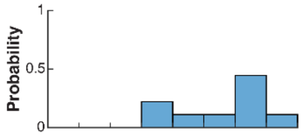	2.06	0.55	0.41	0.22	intermediate mean response intermediate purity most neurons are activated
t_4	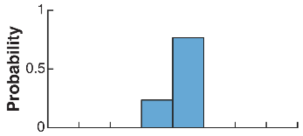	0.79	0.90	0.026	0.023	low mean response (baseline) high purity
t_5	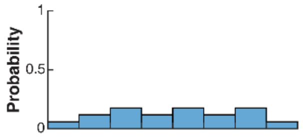	2.90	0.080	0.026	0.002	low mean response low purity neuronal responses are almost uniformly distributed
t_6	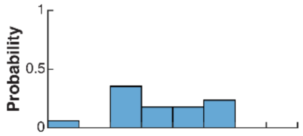	2.15	0.51	-0.1	-0.051	low mean response intermediate purity similar number of neurons are activated or inhibited
t_7	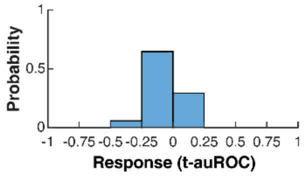	1.17	0.82	-0.077	-0.063	low mean response (return to baseline) high purity

Fig. S17. Examples to provide intuition for purity and consistent-response metrics for groups of molecularly defined cell types.
(**A**) Responses of individual MC5-Crh (left) and MC8-Penk (right) neurons during fear retrieval. Multi-colored lines: response traces of individual neurons; red horizontal lines: 8 response levels used to calculate response distributions in (B); gray vertical lines: 7 example timestamps for the analysis in (B). (**B**) Distributions of the responses of individual neurons at the 7 timestamps in (A) and their corresponding entropies, purities, mean responses and consistent-responses. Formulas for entropy, purity and consistent-response are shown. Brief descriptions of neuronal responses at the 7 timestamps are listed in the last column. Note, a response different than zero with 0.5 purity typically means activation or inhibition distributed along different activation or inhibition response levels respectively; a cell type with zero mean response and 0.5 purity usually has similar number of activated and inhibited neurons; a cell type with zero response and zero purity means the same number of activated and inhibited neurons uniformly distributed across the possible response magnitude range.

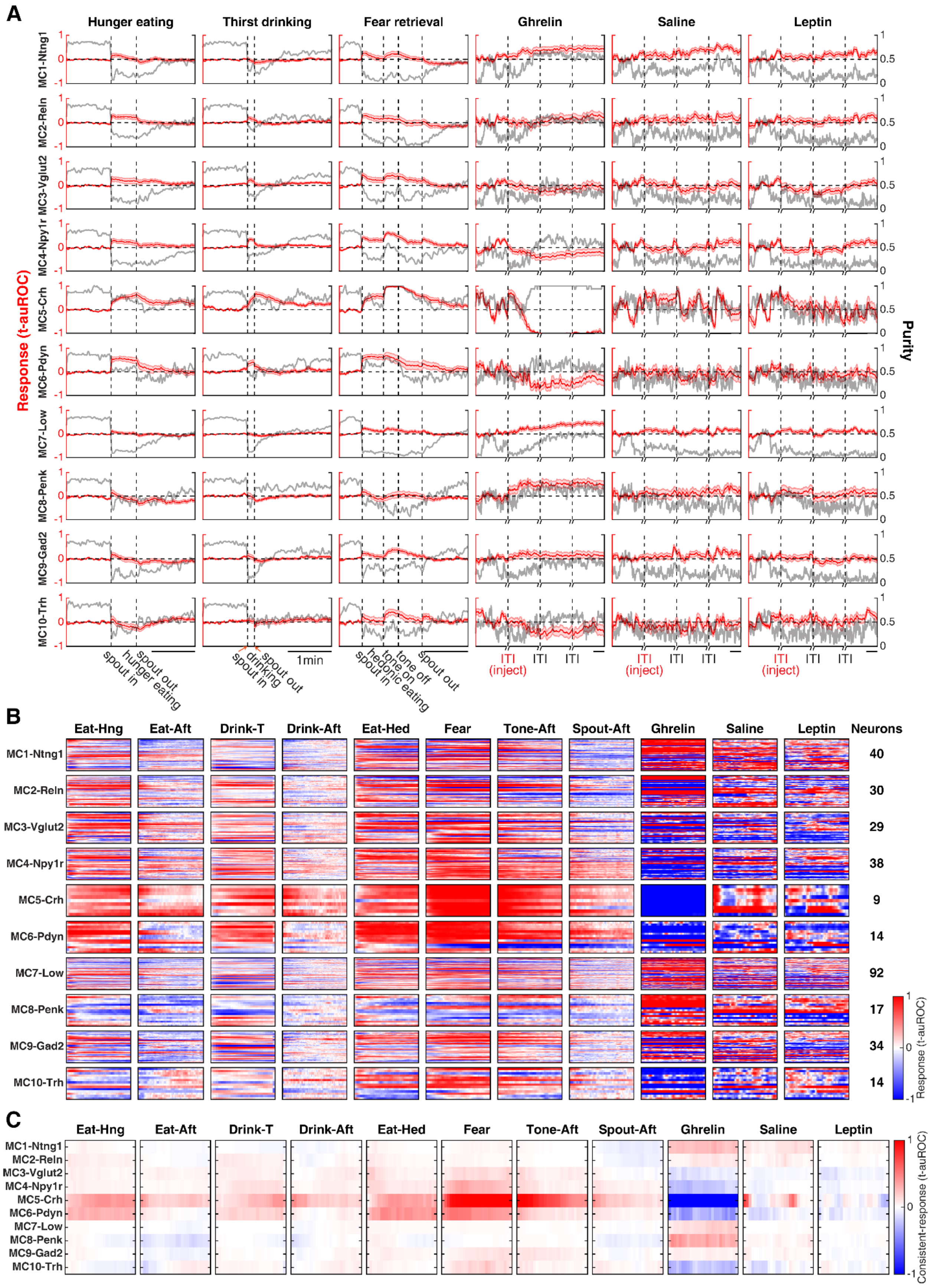

A
Hunger eating
Thirst drinking
Fear retrieval
Ghrelin
Saline
Leptin
MC1-Ntng1
MC2-Reln
MC3-Vglut2
MC4-Npy1r
MC5-Crh
MC6-Pdyn
MC7-Low
MC8-Penk
MC9-Gad2
MC10-Trh
Response (t-auROC)
Purity
spout in
hunger eating
spout out
drinking
1min
hedonic eating
tone on
tone off
ITI (inject)
ITI
B
Eat-Hng
Eat-Aft
Drink-T
Drink-Aft
Eat-Hed
Fear
Tone-Aft
Spout-Aft
Neurons
40
30
29
38
9
14
92
17
34
14
Response (t-auROC)
C
Consistent-response (t-auROC)

Fig. S18. Temporal dynamics of molecularly defined PVH cell types across multiple behavioral states.

(**A**) Average temporal responses and purities of all PVH cell types across all functional imaging experiments. Red shaded lines: the average responses ± SEM across neurons. Gray lines: purity. Temporal scale bar: 1 min. (**B**) Responses of individual neurons in each PVH cell type in each behavioral state. Neurons: number of neurons in corresponding cell type. (**C**) Temporal consistent-responses of all PVH cell types across all behavioral states (26 time bins).

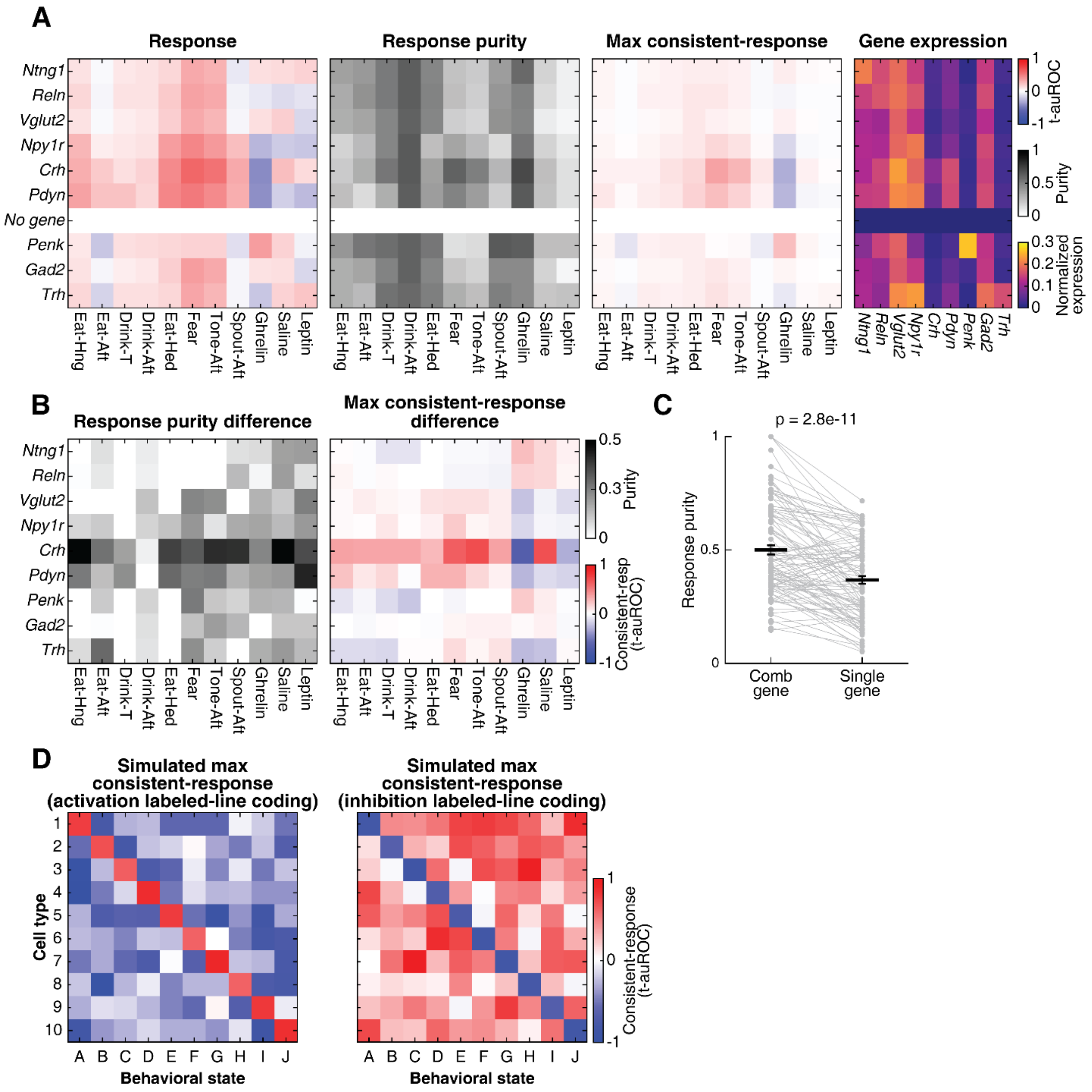

Fig. S19. Comparisons of response patterns from cell types defined by one gene or multiple genes across 11 behavioral states.

(**A**) Temporal maximum for consistent-response and corresponding response and purity of PVH cell types defined by binary expression of single genes across 11 behavioral states. *No gene* row is used for matching these maps with Fig. 5H. Note that cells are not uniquely assigned to a single row in (A) due to co-expression relationships (see Methods for thresholding procedure). (**B**) Difference in response purity and consistent-response between the corresponding maps in Fig. 5H and in (A). This shows that purity and consistent-response are usually higher for cell types defined by a combination of genes. (**C**) Significant difference in response purities of cell types defined by combinatorial gene expression or by single genes (paired-sample *t*-test). (**D**) Simulations of maximum consistent-response expected for labeled-line coding. These patterns

are considerably different from the pattern in Fig. 5H. Left: activation coding. Right: inhibition coding.

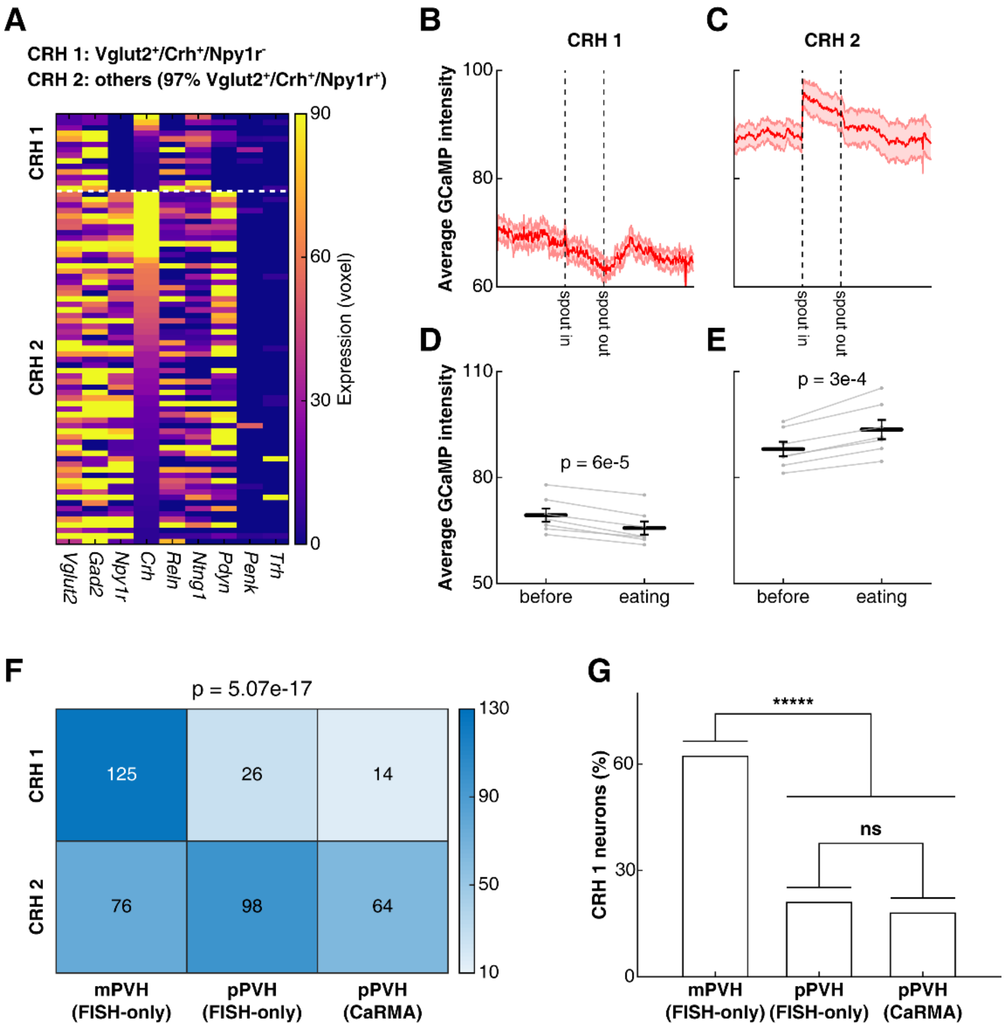

Fig. S20. Responses of two CRH neuron subtypes during hunger-eating.
(**A**) Two subtypes of CRH neurons from CaRMA imaging and their gene expression profiles. Note, MC5-Crh neurons are exclusively a subset of high *Crh*-expressing CRH-2 neurons. (**B, C**) CRH-1 neurons rapidly reduce their activity (B), while CRH-2 neurons immediately increase their activity (C) during hunger-eating. Shaded red lines: mean GCaMP intensity ± SEM across trials. Note, that the rapid increase in CRH-2 activity is distinct from MC5-Crh activity, which ramps more slowly during food consumption in hunger (Fig. 5G). This is because CRH-2 contains a considerable portion of the neurons from MC6-Pdyn, which are fast-responding (Fig. 5G). This is due to the co-expression relationship of *Crh* and *Pdyn* (apparent in A). This also highlights a limitation of Cre-line-based photometry of these neurons which is expected to capture even low-expressing CRH neurons, many of which are high *Pdyn*-expressing and members of MC6-Pdyn. In contrast, CaRMA imaging measures the expression level of each gene in each GCaMP-expressing cell and then uses unsupervised classification algorithms that implicitly threshold expression levels so that these molecularly defined clusters (MCs) are comprised of high-expressing cells for a given gene. (**D, E**) Changes in average GCaMP intensity of CRH-1 (D) and CRH-2 (E) neurons before and during hunger-eating. Each dot represents the mean value of GCaMP intensity for all CRH-1 and CRH-2 neurons before or during hunger-eating in one trial. Mean± SEM; paired-sample *t*-test. (**F**) Proportions of CRH-1

and CRH-2 neurons in mPVH (FISH-only), pPVH (FISH-only), and pPVH (CaRMA). (**G**) CRH-1 subtype is significantly enriched in mPVH compared with pPVH, explaining the CRH-inhibited responses reported by fiber photometry population measurements of PVH^{CRH} neuron calcium activity. Chi-squared test (F) followed by pairwise comparisons with Bonferroni correction (G). ***** $p < 10^{-5}$. Statistics in Table S2.

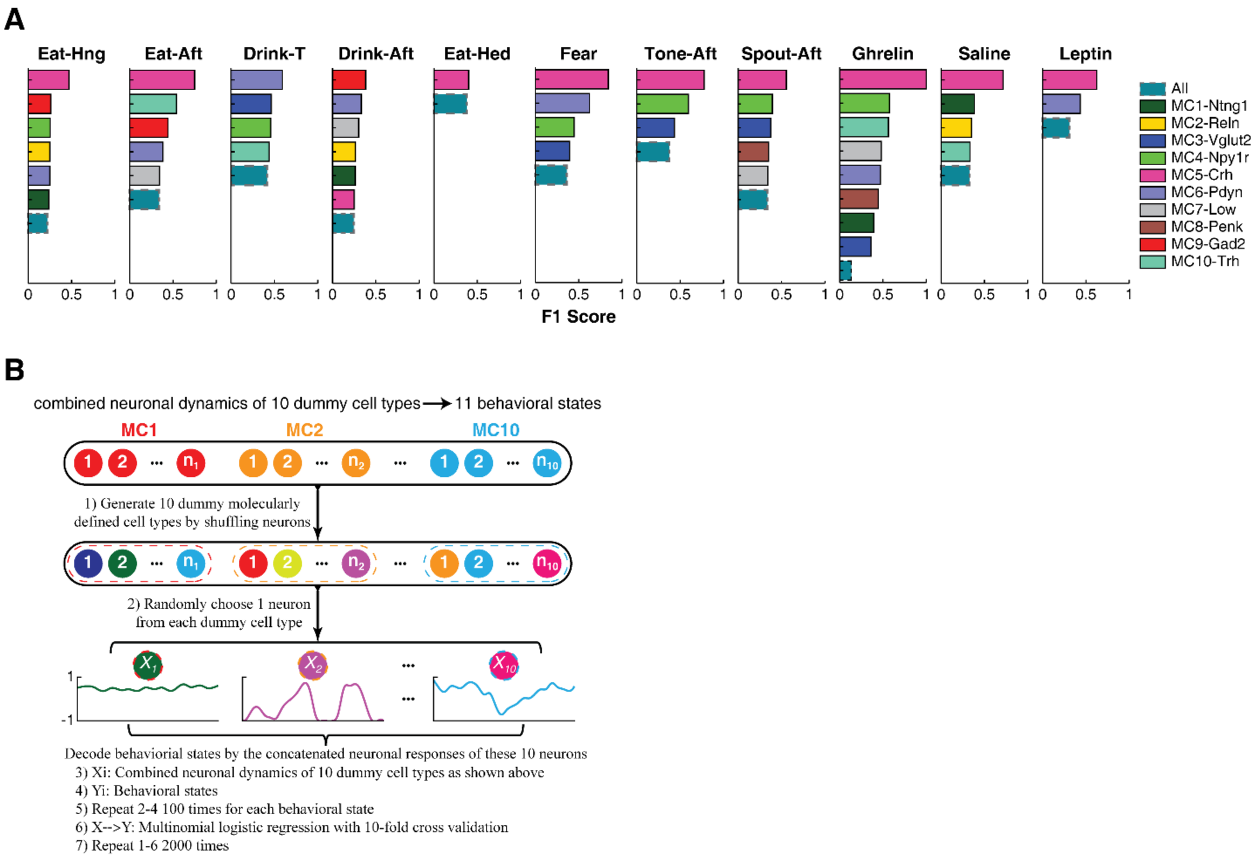

Fig. S21. Decoding behavioral states with neuronal dynamics of PVH cell types.
(**A**) Decoding performances of individual molecularly defined PVH cell types measure by their F1 scores in different behavioral states. Cell types are ordered by their decoding performances and displayed when their performances are higher than the performance decoded from all neurons (All). (**B**) Schematic procedure of decoding behavioral states with the combined temporal response profile of the dummy PVH cell types, one neuron from each dummy cell type.

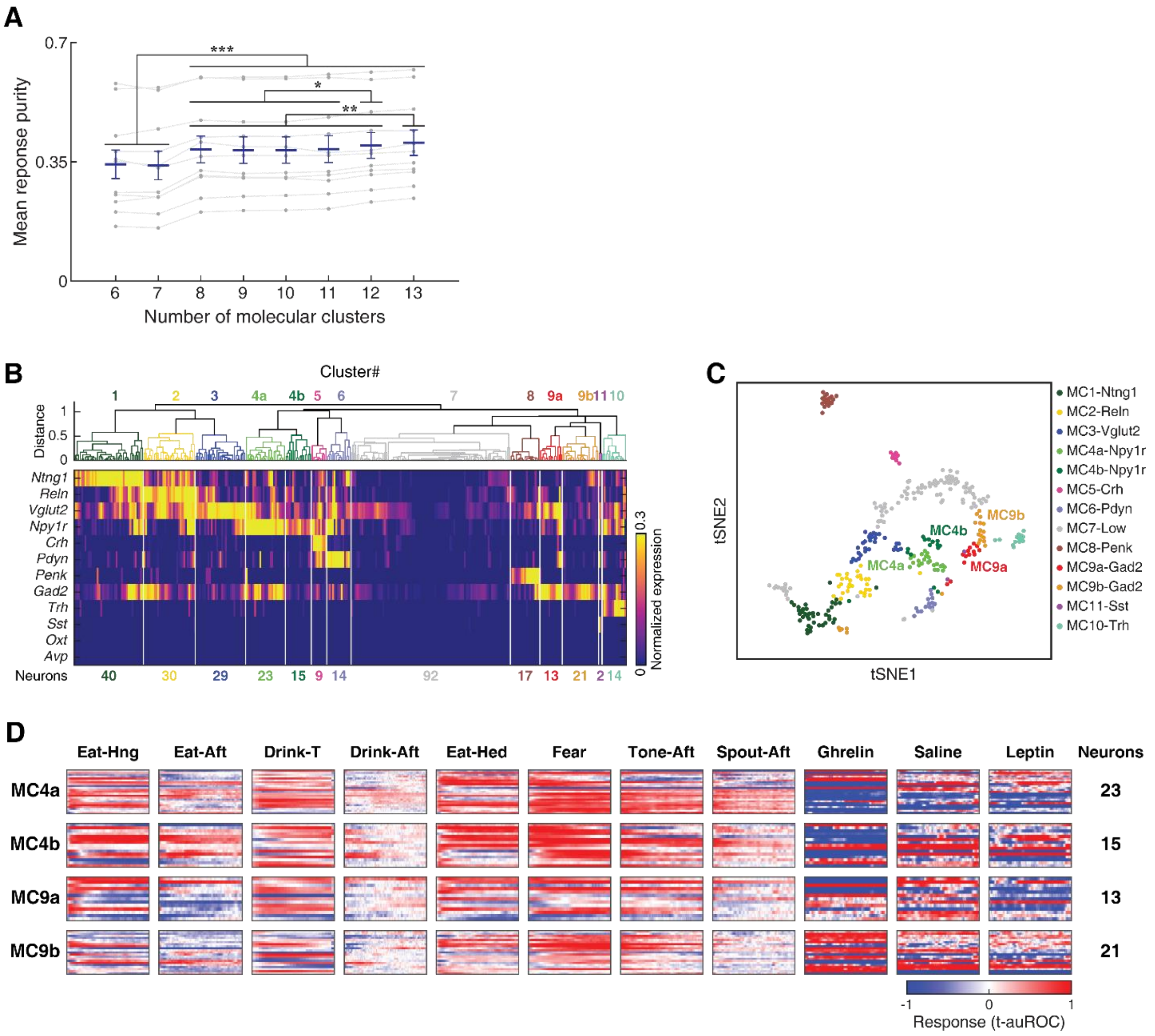

Fig. S22. Effect of cluster threshold on the mean response purity of corresponding cell types across all behavioral states.
(**A**) Differences in mean response purities of different number of molecular clusters across all behavioral states by varying clustering threshold. Purity showed marginal but significant increases by lowering the cluster threshold, thereby increasing the number of clusters to 13. Blue lines: mean response purities; error bars: SEM; gray connected circles: mean response purities of different number of molecular clusters in one behavioral state (one-way repeated measures ANOVA followed by Tukey-Kramer test). (**B**) The expression profile of 13 molecularly defined cell types from the CaRMA dataset. The expression profile and hierarchical organization are the same as in Fig. 5B but use lower clustering threshold. This further splits MC4-Npy1r and MC9-Gad2 (fig. S18B) each into two additional clusters as MC4a: high-*Npy1r*$^+$/high-*Gad2*$^+$; MC4b: medium-*Npy1r*$^+$/low-*Gad2*$^+$; MC9a: high-*Gad2*$^+$/high-*Vglut2*$^+$; MC9b: high-*Gad2*$^+$/low-*Vglut2*$^+$. (**C**) tSNE plot of the 13 molecularly defined cell types in (B) showing the splits of MC4-Npy1r and MC9-Gad2 from Fig. 5C. (**D**) Responses of individual neurons from MC4a, MC4b, MC9a and MC9b cell types in each behavioral state. Neuronal responses of other PVH cell types in

each behavioral state are the same as in fig. S18B. Neurons: number of neurons in corresponding cell type. * $p < 0.05$, ** $p < 0.01$, *** $p < 0.001$. Statistics in Table S2.

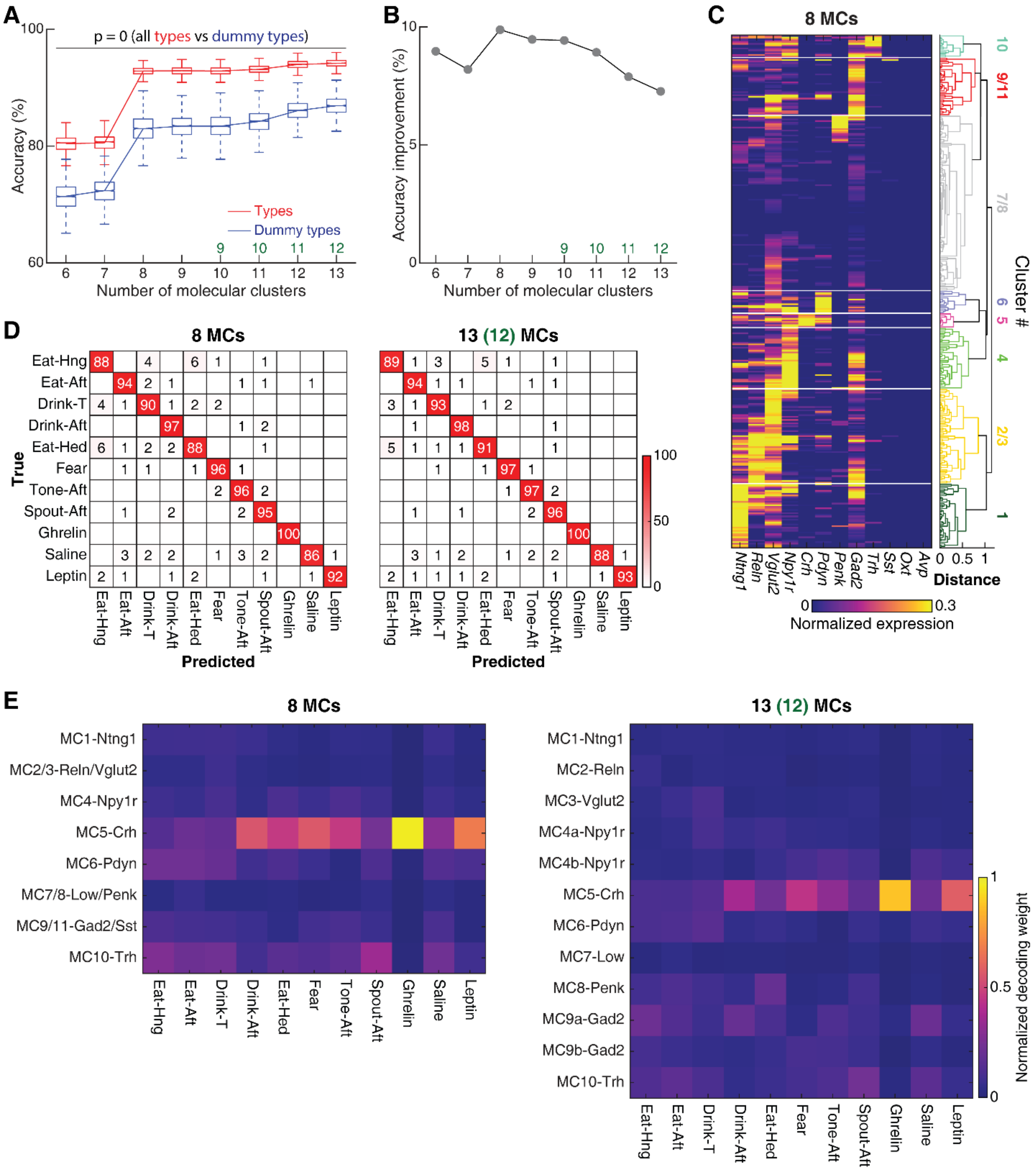

Fig. S23. Effect of the number of molecular clusters on the robustness of behavioral state decoding.

(**A**) Effect of the number of molecular clusters on decoding accuracy by adjusting the threshold for hierarchical molecular clustering in Fig. 5B. Red box plots: decoding accuracies with the combined neuronal dynamics of *n* neurons from *n* PVH cell types clustered with different thresholds. Blue box plots: decoding accuracies with the combined neuronal dynamics of *n* neurons from *n* dummy cell types that scramble cell type information. Except for the number of

cell types, procedures for decoding behavioral states with PVH and dummy cell types are the same as in Fig. 6E and fig. S21B, respectively. The green number is the effective number (*n*) of cell types for behavioral state decoding, because the Sst cluster with only 2 neurons was excluded from the decoding analysis. Red line: mean decoding accuracies with the corresponding PVH cell types. Blue line: mean decoding accuracies with the corresponding dummy cell types. $n = 6$ to 12. Decoding accuracy generally increased with the number of molecular clusters, but this was also the case for the dummy cell types. (**B**) Improvement in decoding accuracies by using the molecular information from different number of molecular clusters. The largest improvement in decoding accuracy over the dummy cell types was from 8 molecular clusters, which has the highest threshold that separates the MC5-Crh and MC6-Pdyn clusters into distinct cell types (C). The highest overall purity from thresholding clusters (13 MCs, fig. S22A) was associated with the lowest improvement in decoding accuracy relative to the dummy cell types, which is due to redundant information associated with increased splitting of MCs combined with increased decoding power of dummy cell types as the number of decoding neurons increases. (**C**) The expression profile of 8 molecularly defined cell types of CaRMA neurons. The expression profile and hierarchical organization are the same as in Fig. 5B but using higher clustering threshold. This merges MC2-Reln with MC3-Vglut2, MC7-Low with MC8-Penk, and MC9-Gad2 with MC11-Sst. (**D**) The average confusion matrices with the combined neuronal dynamics of 8 neurons from 8 MCs (left matrix) or with the combined neuronal dynamics of 12 neurons from 12 MCs (right matrix) using the same procedure in Fig. 6E but different number of MCs. (**E**) Normalized decoding weights of individual cell types for all behavioral state using 8 (left) or 12 MCs (right). Definition and calculation of normalized decoding weights are described in fig. S24, A and B.

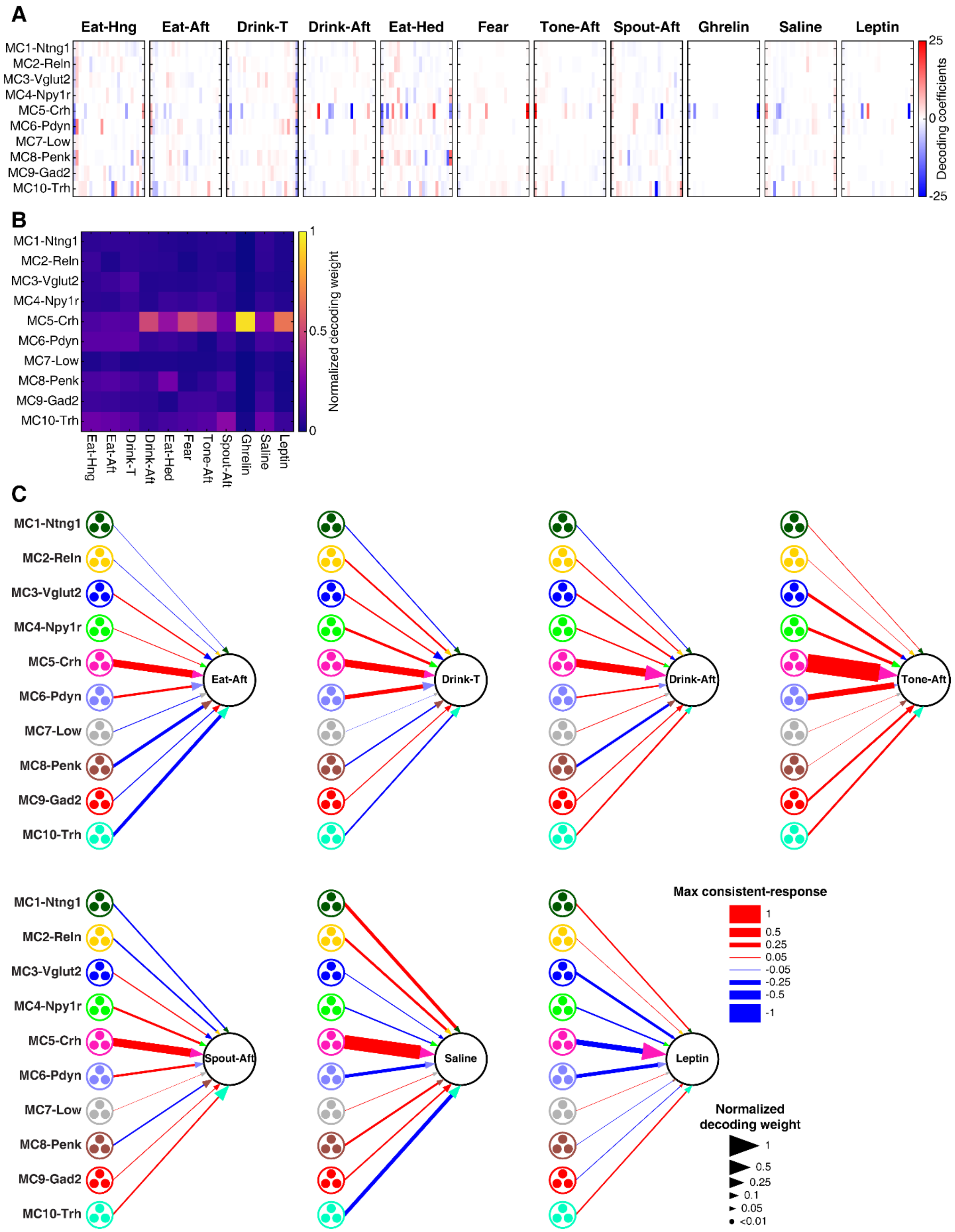
A
Eat-Hng
Eat-Aft
Drink-T
Drink-Aft
Eat-Hed
Fear
Tone-Aft
Spout-Aft
Ghrelin
Saline
Leptin
MC1-Ntng1
MC2-Reln
MC3-Vglut2
MC4-Npy1r
MC5-Crh
MC6-Pdyn
MC7-Low
MC8-Penk
MC9-Gad2
MC10-Trh
25
0
-25
Decoding coefficients
B
1
0.5
0
Normalized decoding weight
C
Max consistent-response
1
0.5
0.25
0.05
-0.05
-0.25
-0.5
-1
Normalized decoding weight
1
0.5
0.25
0.1
0.05
<0.01

Fig. S24. Cell type ensemble response-decoding diagrams

(**A**) Average multinomial regression coefficients of individual cell types for each behavioral state. Values in each row are the average decoding coefficients for the temporal response (26 bins) of one cell type (average value of the coefficients from the 2000 prediction models). (**B**) Normalized decoding weights of individual cell types for all behavioral state. The decoding weight of one cell type for each behavioral state is the summation of the absolute values of the average decoding coefficients of that cell type for that behavioral state. Then the decoding weights of all cell types for each behavioral state are normalized, so that the summation of the normalized decoding weights of all cell types for each behavioral state is 1. This functional organization was maintained when we varied the number of MCs by adjusting the clustering threshold (fig. S23E). (**C**) Diagrams with temporal maximum of consistent-response (proportional to line width) for each cell type and their decoding weights (proportional to arrowhead area) for other 7 behavioral states.

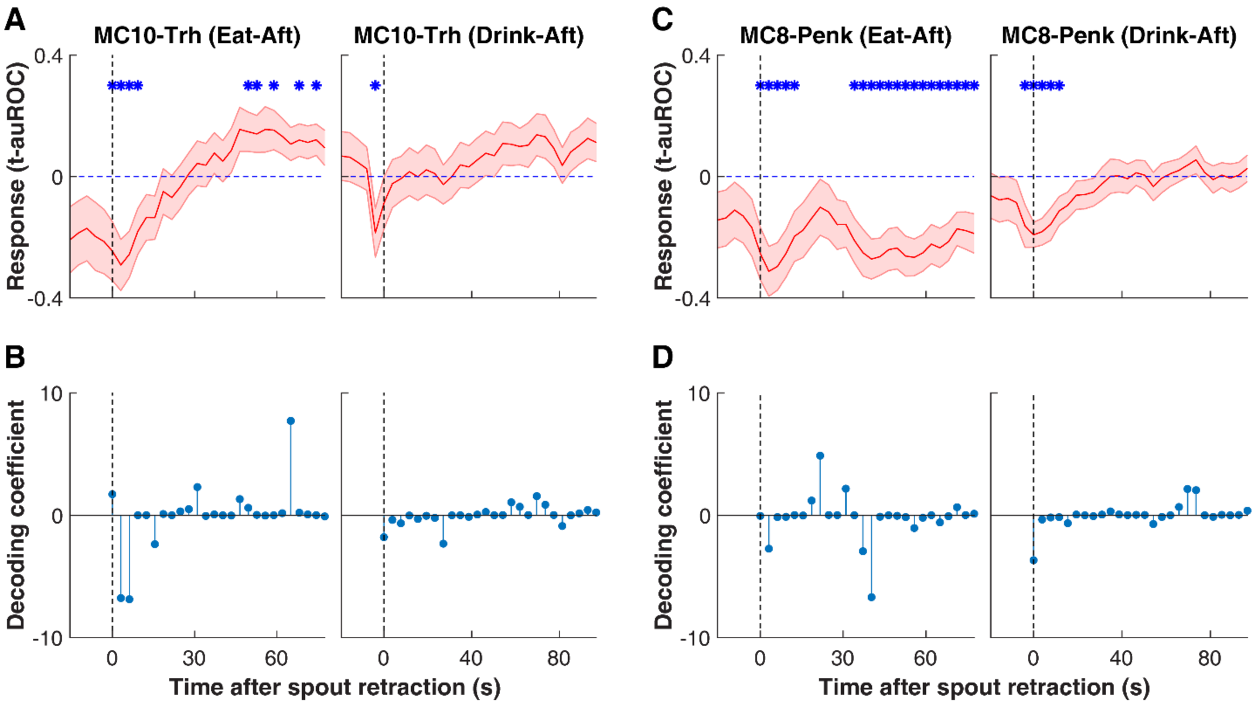

Fig. S25. Temporal responses and decoding coefficients of MC10-Trh and MC8-Penk neurons after food and water consumption

(**A**) Temporal responses of MC10-Trh neurons after hunger-eating (left) and thirst-drinking (right). (**B**) Average coefficients of MC10-Trh neuron responses for decoding after hunger-eating (Eat-Aft, left) and thirst-drinking (Drink-Aft, right) states. (**C**) Temporal responses of MC8-Penk neurons after hunger-eating (left) and thirst-drinking (right). (**D**) Average coefficients of MC8-Penk neuron responses for decoding Eat-Aft (left) and Drink-Aft (right) states. Red shaded line in (A, C): mean ± SEM of 26-bin temporal responses across neurons. Blue * in (A, C): responses significantly different from 0 (blue dashed line), one-sample *t*-test.

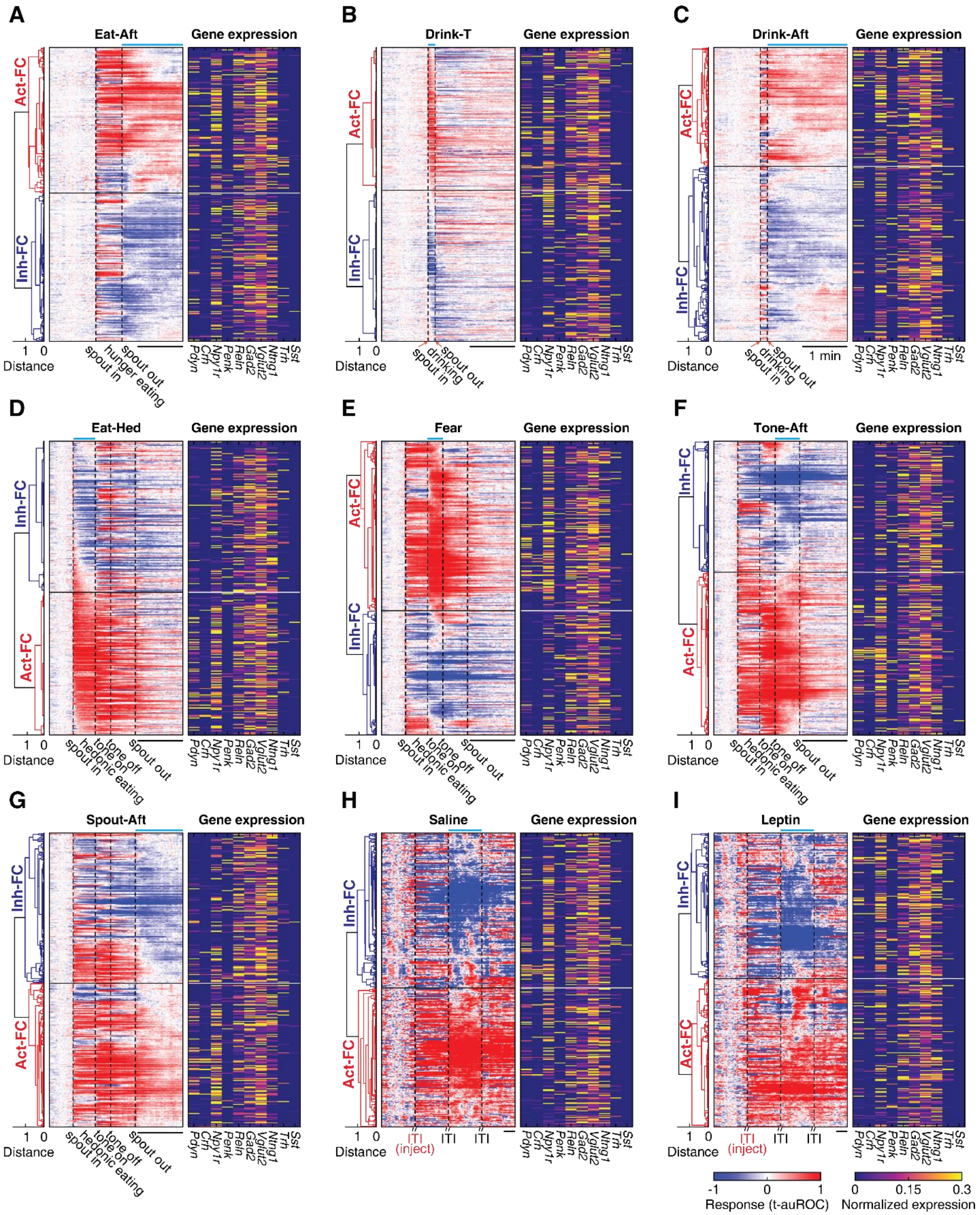

Fig. S26. Functional clustering of PVH neurons and their corresponding gene expression profiles in 9 behavioral states.

(**A-I**) Hierarchical clustering of PVH neurons based on their responses in Eat-Aft (A), Drink-T (B), Drink-Aft (C), Eat-Hed (D), Fear (E), Tone-Aft (F), Spout-Aft (G), Saline (H) and Leptin (I) state. Gene expression profiles of individual neurons are shown on the right. Blue bar on top marks the behavioral state. Description of behavioral tasks and abbreviations are in fig. S7. Temporal scale bar: 1 min.

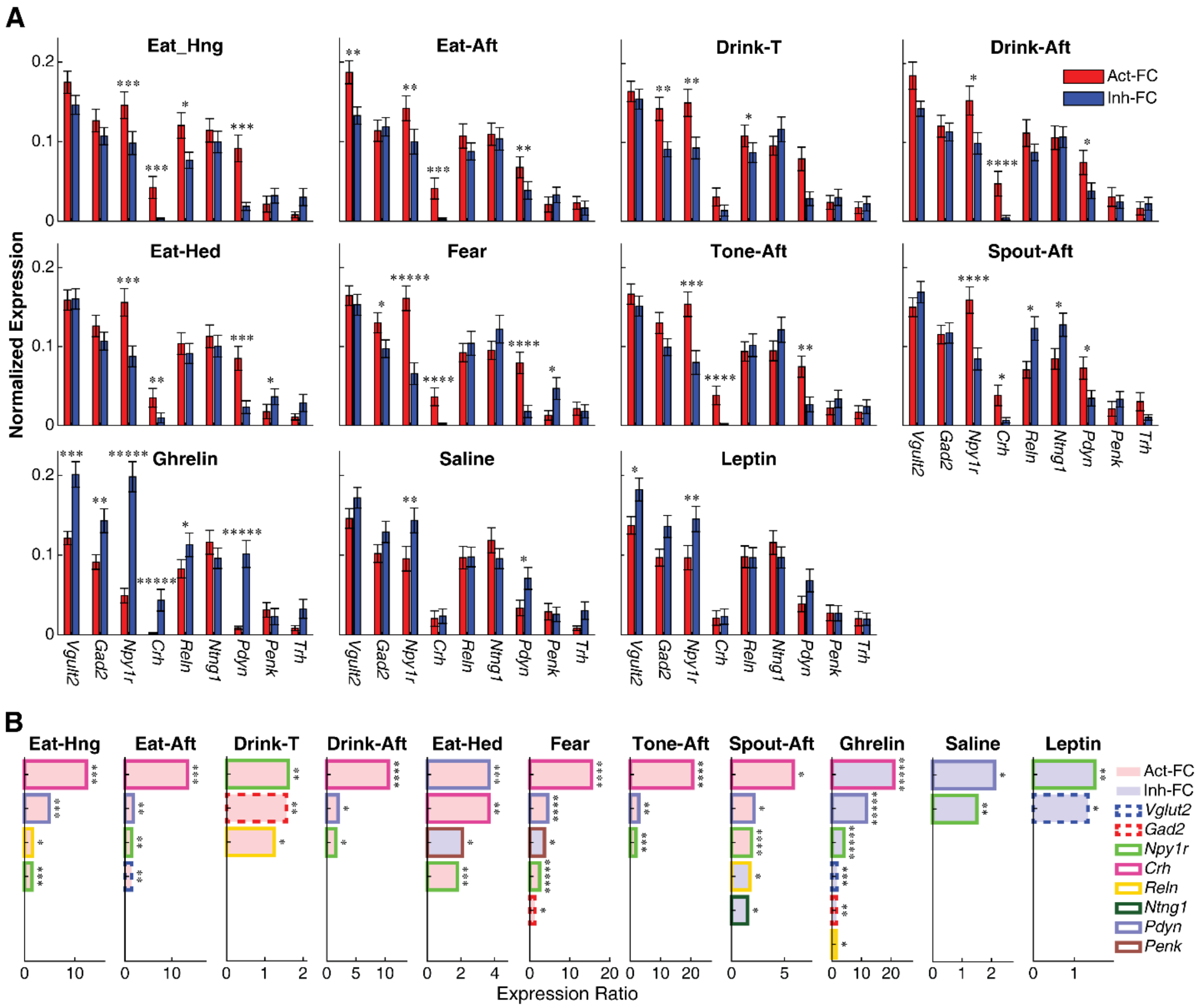

Fig. S27. Differential enrichment of marker-genes in 11 behavioral states.
(**A**) Differences in expression level of marker-genes between the Act-FC and Inh-FC in each behavioral state. Error bars: mean ± SEM. (**B**) Enrichment of marker-genes in the functional clusters across 11 behavioral states. Gene enrichments were ranked by expression ratio (average expression in the enriched FC divided by average expression in the other FC). The enriched genes are indicated by the bar outlines, and the bar fill colors indicate the enriched functional clusters. * $p < 0.05$, ** $p < 0.01$, *** $p < 0.001$, **** $p < 10^{-4}$, ***** $p < 10^{-5}$. Statistics in Table S2.

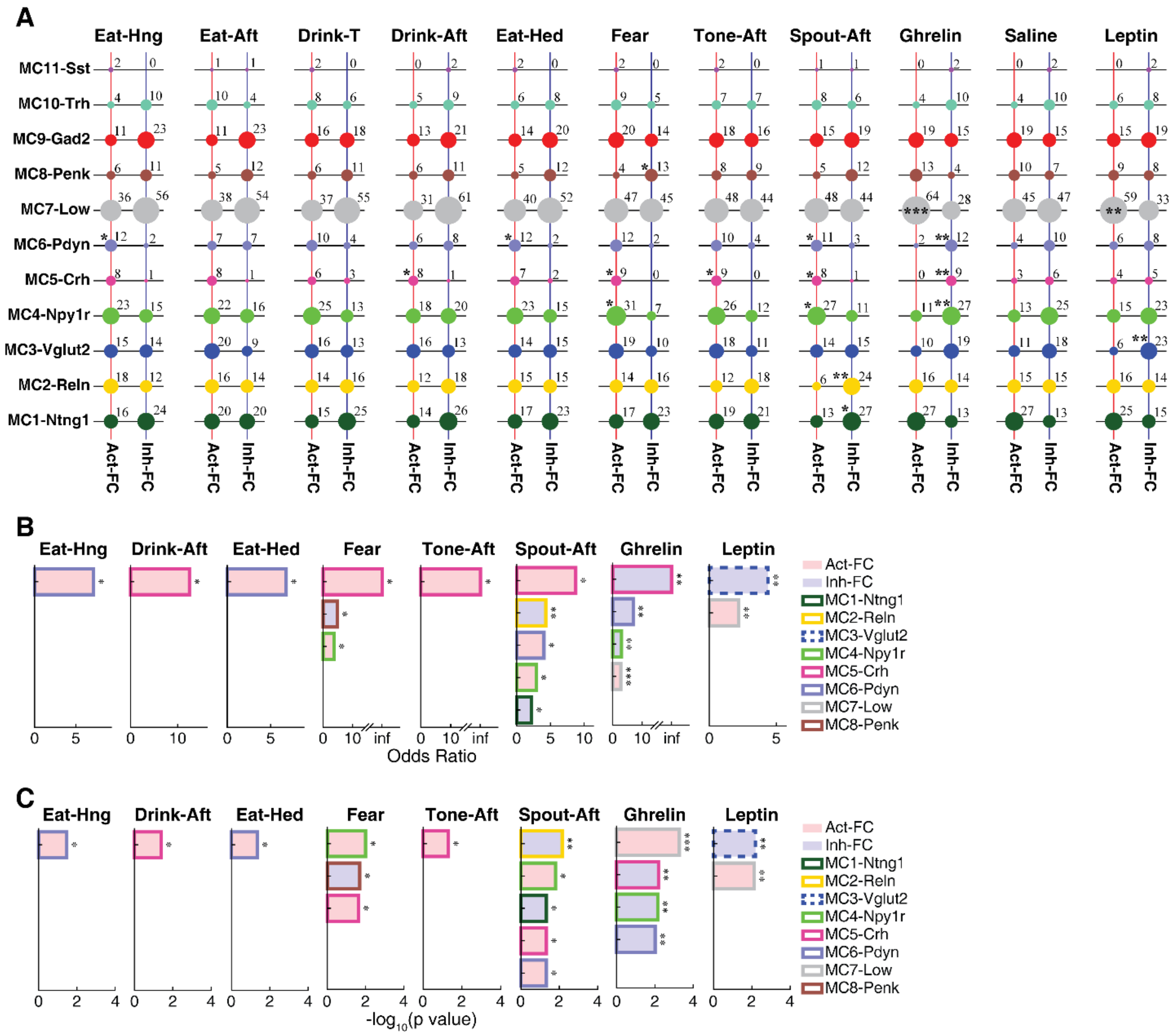

Fig. S28. Enrichment analyses of molecularly defined cell types in 11 behavioral states. (**A**) Differential enrichment of molecularly defined cell types between Act-FC and Inh-FC in each behavioral state. Cell counts are labelled and proportional to circle area. (**B-C**) Enrichment of molecularly defined cell types in the functional clusters across 8 behavioral states. Cell type enrichments were ranked by odds ratio (B) and by -log$_{10}$(p value) (C). The enriched cell types are indicated by the bar outlines, and the bar fill colors indicate the enriched functional clusters. * $p < 0.05$, ** $p < 0.01$, *** $p < 0.001$. Statistics in Table S2.

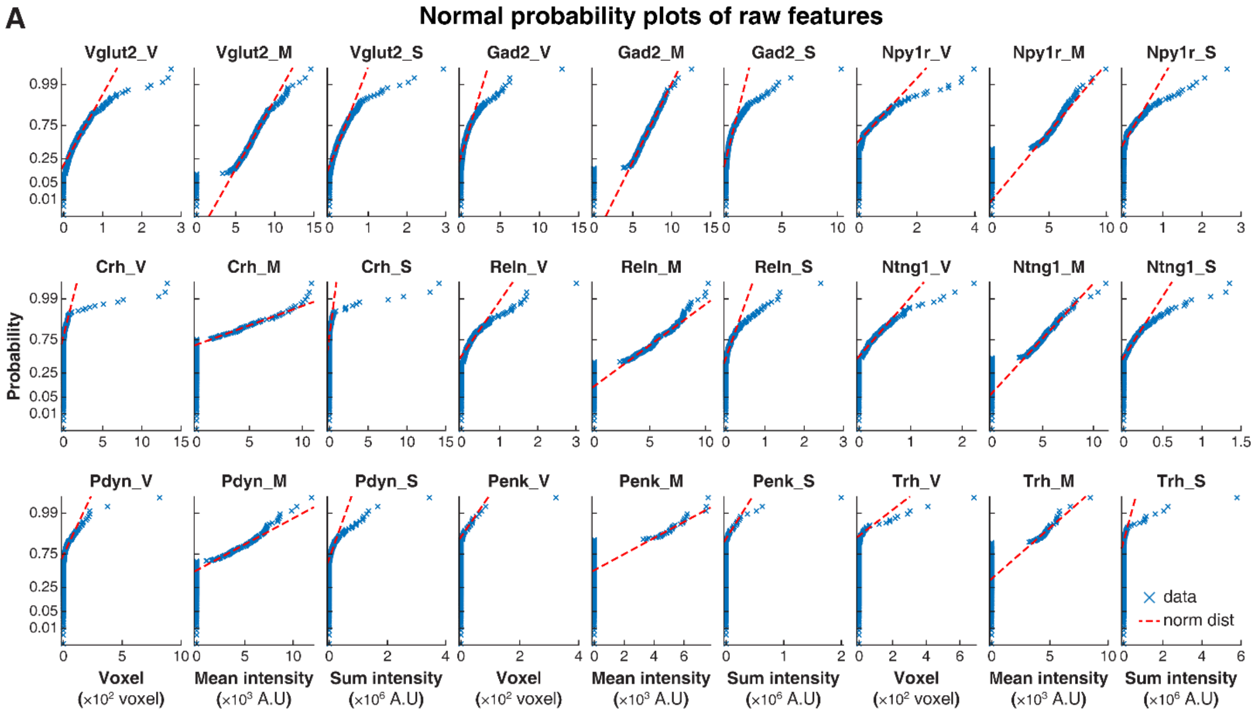

B *N01_ReLu_ln* **normalization of features**

(1)

$$fl = \begin{cases} \ln(f\_raw+1) & \text{if f\_raw is voxel or sum intensity} \\ f\_raw & \text{if f\_raw is mean intensity} \end{cases}$$

(2)

$$flp\_min = min(fl|fl>0)$$
$$fl\_max = max(fl)$$
$$flp\_range = fl\_max - flp\_min$$
$$fl\_thresh = max(0.99*flp\_min, flp\_min-0.01*flp\_range)$$

(3)

$$fn = \frac{fl-fl\_thresh}{fl\_max-fl\_thresh}$$

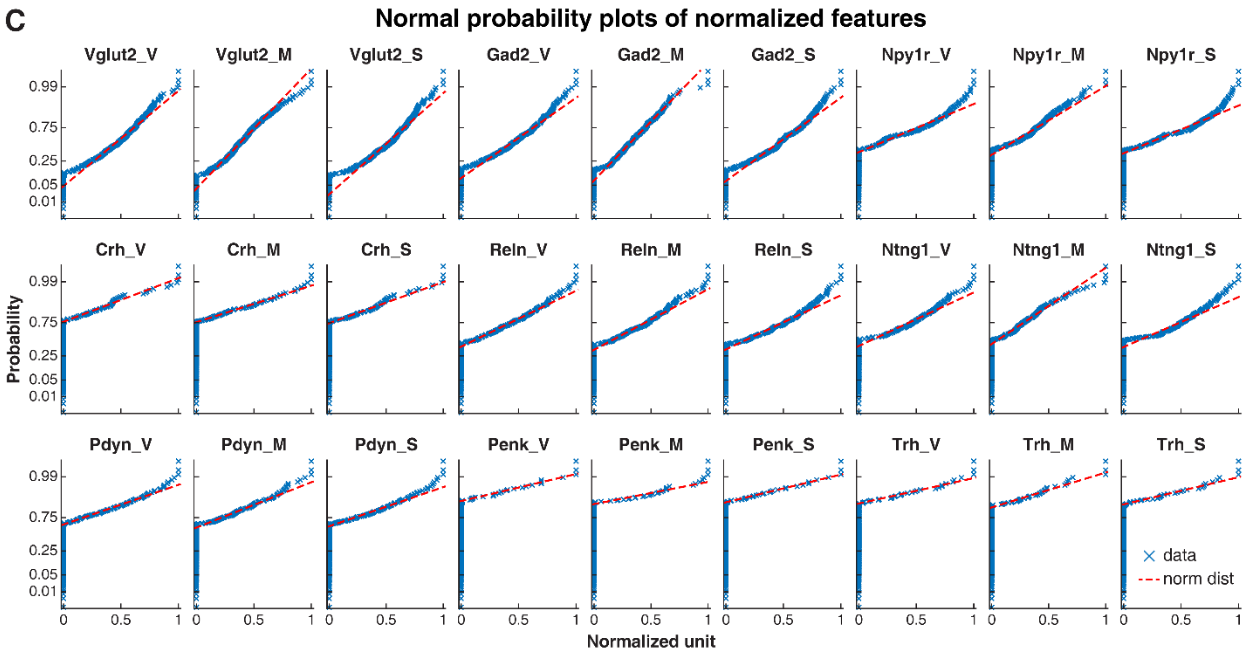

Fig. S29. Normalization of gene expression features

(**A**) Normal probability plots of the 3 raw features from all cells for all genes. The 3 raw features to represent the expression level of gene *xxx* were voxels (*xxx*-V), mean intensity (*xxx*-M) and sum intensity (*xxx*-S). A.U: arbitrary unit. Blue cross: a feature data point from one cell. Red dashed line: reference line represents normal distribution fit for the feature data points (value > 0). If the feature data (value > 0) have a normal distribution, then the data points appear along the reference line, otherwise the data points deviate from the reference line with a certain curvature. These plots showed that the raw mean intensity features of all genes largely had normal distributions with a large gap between feature value = 0 and value > 0, but the raw voxel and sum intensity features of most genes were not normally distributed. (**B**) Formulas for feature normalization. Formula in step1 was used to transform the raw feature data (*f_raw*) so that the transformed features (*fl*) could be normally distributed. The maximum value (*fl_max*) and threshold for rectification (*fl_thresh*) of each feature were found in step2. In step3, the transformed features (*fl*) were rectified and scaled to [0 1]. The rectification was used to increase the dynamic range of mean intensity features by reducing the gap between feature value = 0 and value > 0. (**C**) Normal probability plots of the 3 normalized features from all cells for all genes. These plots indicated that all normalized features of all genes largely had normal distributions that were continuous within the range [0 1].

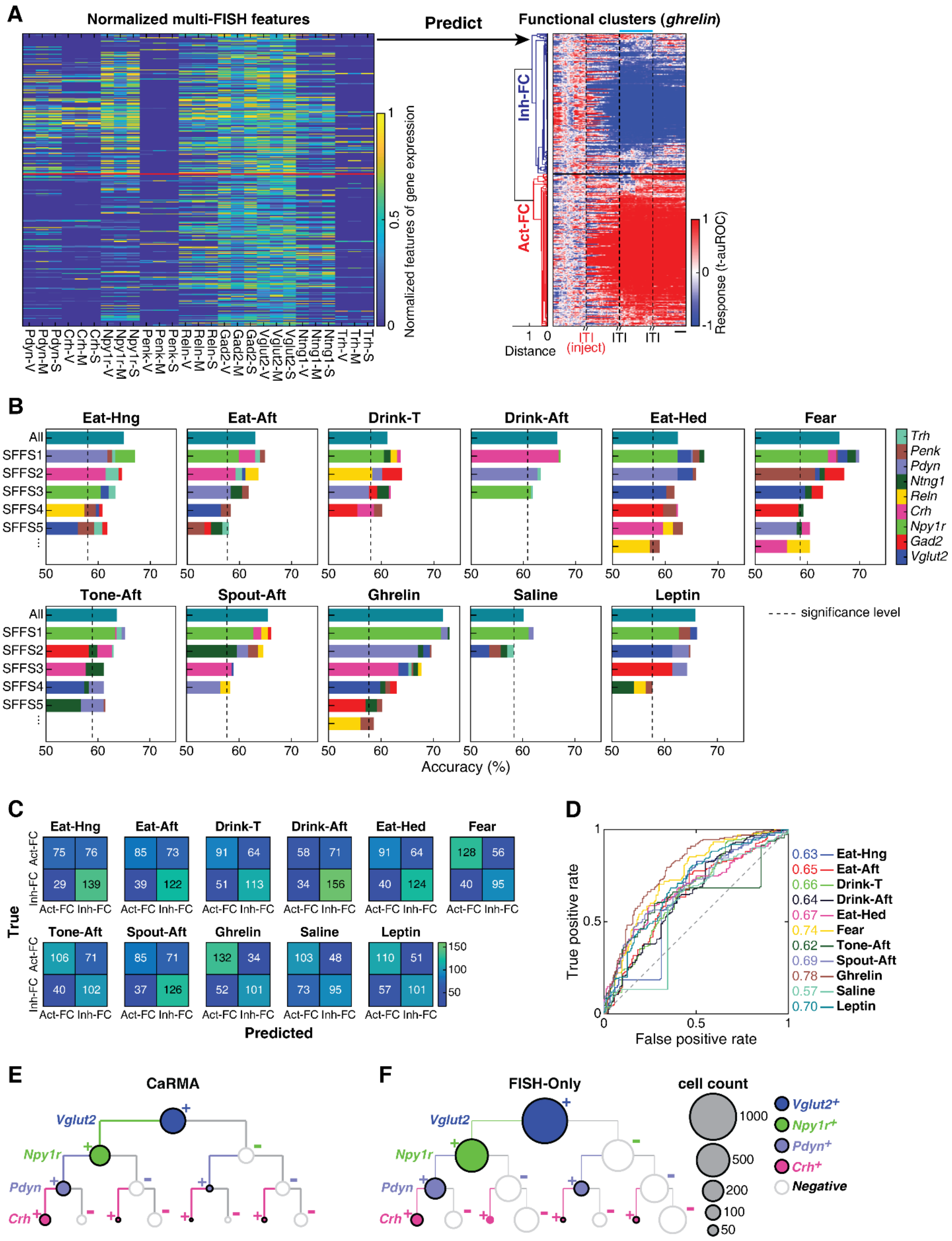
A
Normalized multi-FISH features
Predict
Functional clusters (ghrelin)
Normalized features of gene expression
Inh-FC
Act-FC
Distance
ITI (inject)
ITI
ITI
Response (t-auROC)
B
Eat-Hng
Eat-Aft
Drink-T
Drink-Aft
Eat-Hed
Fear
Tone-Aft
Spout-Aft
Ghrelin
Saline
Leptin
All
SFFS1
SFFS2
SFFS3
SFFS4
SFFS5
Trh
Penk
Pdyn
Ntng1
Reln
Crh
Npy1r
Gad2
Vglut2
significance level
Accuracy (%)
C
True
Predicted
Act-FC Inh-FC
D
True positive rate
False positive rate
0.63 Eat-Hng
0.65 Eat-Aft
0.66 Drink-T
0.64 Drink-Aft
0.67 Eat-Hed
0.74 Fear
0.62 Tone-Aft
0.69 Spout-Aft
0.78 Ghrelin
0.57 Saline
0.70 Leptin
E
CaRMA
Vglut2
Npy1r
Pdyn
Crh
F
FISH-Only
cell count
1000
500
200
100
50
Vglut2+
Npy1r+
Pdyn+
Crh+
Negative

Fig. S30. Gene expression profiles of individual neurons are predictive for their functional clusters in multiple behavioral states.

(**A**) Normalized features of marker-genes from individual neurons (left) and their corresponding functional clusters (FCs, right) after ghrelin injection. Normalized voxels (*xxx*-V), mean intensity (*xxx*-M) and sum intensity (*xxx*-S) were the 3 features of gene *xxx* for the prediction of FCs. (**B**) The predictive accuracies of individual genes and their combinations in multiple behavioral states by mSFFS. All: prediction with all marker genes. SFFS*n*: n^{th} round of sequential forward feature selection, where the most predictive genes from the previous rounds (<*n*) were removed from the candidate gene set. Only rounds with predictive power above the significance level were displayed. In each round, the predictive power was stacked with the sequentially additive contributions of the selected genes measured from mSFFS, so that the predictive power of any sequential combination of these selected genes can be easily evaluated. (**C**) Confusion matrix of optimal prediction of FCs, which was evaluated by mSFFS, in each behavioral state. (**D**) ROC curve of optimal prediction of FCs in each behavioral state. auROCs are listed on the right. (**E-F**) Hierarchical expression of *Vglut2*, *Npy1r*, *Pdyn* and *Crh* genes in the PVH samples from CaRMA imaging (E) and from the multiplexed FISH-only dataset from Fig. 2 (F). Circle area is proportional to cell counts.

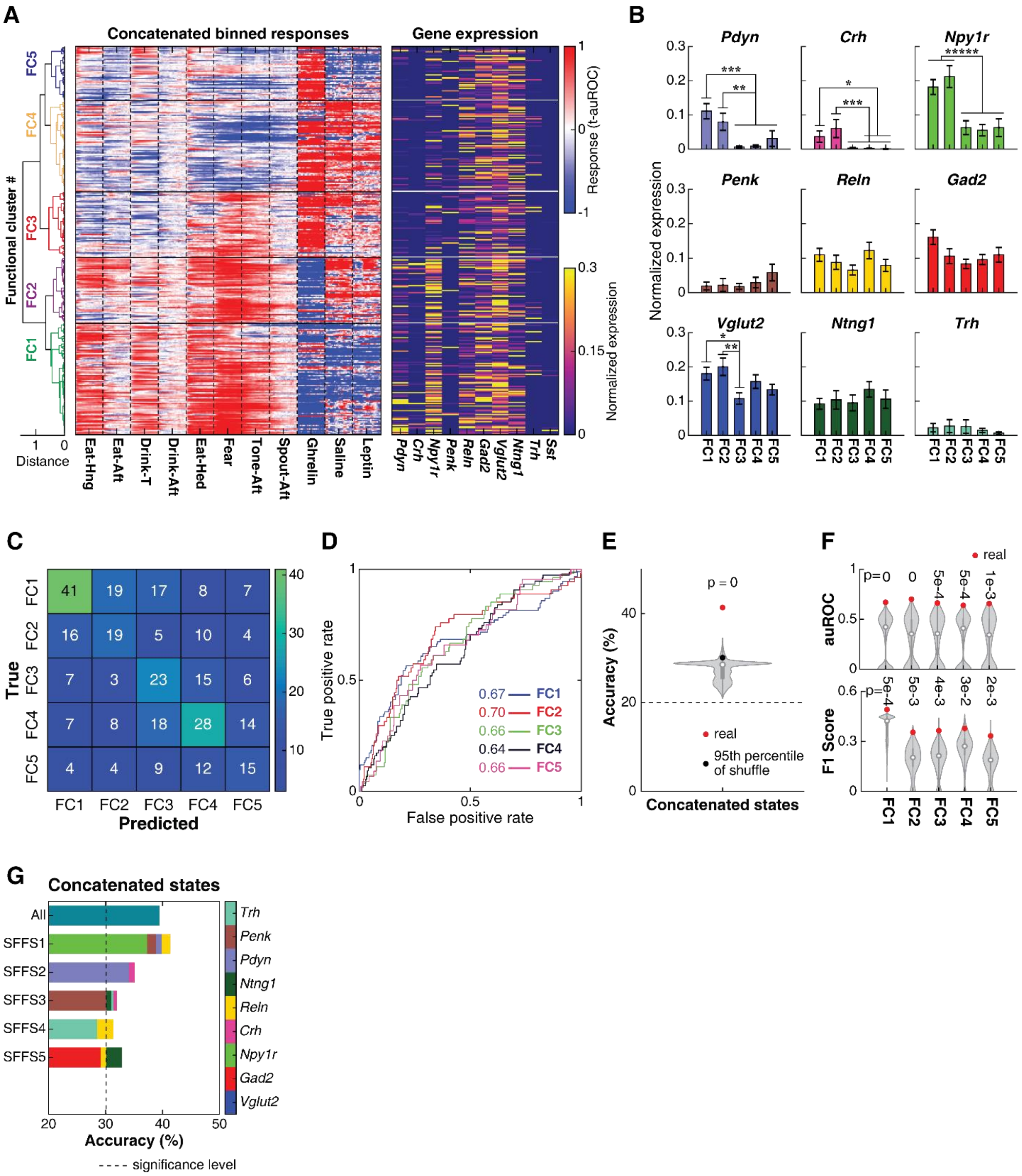

Fig. S31. Relationship between gene expression profiles of individual neurons and functional clusters based on their concatenated responses across multiple behavioral states. (**A**) Hierarchical clustering of PVH neurons based on their concatenated binned responses across 11 behavioral states. Gene expression profiles of individual neurons are shown on the right. To balance the sample sizes of responses from all behavioral states, the response in each behavioral state was binned into 26-bin response and then concatenated across all behavioral states for each

neuron. (**B**) Differences in expression level of marker-genes among the 5 FCs. FC1 and FC2 showed strong gene expression enrichment of *Npy1r*, *Pdyn*, and *Crh.* Error bars: mean ± SEM. (**C**) We used multinomial logistic regression to predict these FCs by gene expression profiles. Confusion matrix of optimal prediction of the 5 FCs, which was evaluated by mSFFS in (G). The relatively high false prediction rates between FC1/FC2 and FC3/FC4 are associated, in part, with the low performance accuracies of our marker-genes to predict FCs in some behavioral states (such as leptin and saline injection in Figure 8D) that were included when we concatenated all behavioral states. In principle, additional marker-genes might improve predictive performance in these states as well as the FCs from the concatenated neuronal responses across all behavioral states. (**D**) ROC curves of optimal prediction of the 5 FCs. The numbers listed are the auROCs. (**E**) The predictive power of gene expression profiles for all 5 FCs was statistically significant by comparing the optimal accuracy with the shuffled accuracies. (**F**) The predictive power of gene expression profiles for individual FCs were each statistically significant by comparing the optimal auROCs of individual FCs with their shuffled auROCs (top) and the optimal F1 scores of individual FCs with their shuffled F1 scores (bottom). (**G**) The predictive accuracy of individual genes and their combinations for all 5 FCs by mSFFS. All: prediction with all marker-genes. SFFS*n*: n^{th} round of sequential forward feature selection. Only rounds with predictive power above the significance level were displayed. *Npy1r*, *Penk*, *Pdyn*, and *Reln* were the optimal gene predictors for these 5 FCs. As expected, predictive accuracies for 5FCs were less than for predictions of just two functional clusters. Nevertheless, this demonstrated that even a limited number of gene expression features provided statistically significant prediction of many neuron functional classes across multiple behavioral states. The bars were organized in the same way as those in Fig. 8C and fig. S30B. * $p < 0.05$, ** $p < 0.01$, *** $p < 0.001$, ***** $p < 10^{-5}$. Statistics in Table S2.

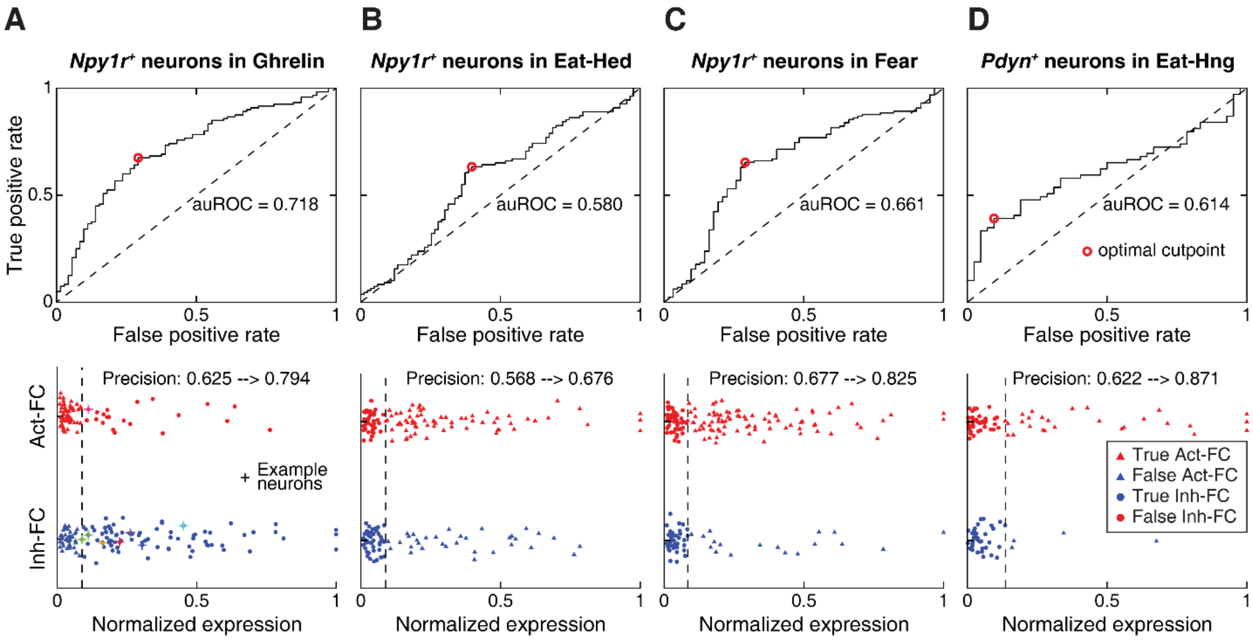

Fig. S32. Improvement in functional classification precision by using a marker-gene expression threshold.

(**A**) Top: ROC curve using *Npy1r* expression level in *Npy1r*$^{+}$ neurons to predict Inh-FC after ghrelin injection and its Youden's optimal cutpoint. Bottom: Classification of *Npy1r*$^{+}$ neurons using the optimal cutpoint above as *Npy1r* expression threshold. Colored cross: example neurons in Fig. 8G (response color in Fig. 8G). (**B**) Top: ROC curve using *Npy1r* expression level in *Npy1r*$^{+}$ neurons to predict Act-FC during hedonic eating and its optimal cutpoint. Bottom: Classification of *Npy1r*$^{+}$ neurons using the optimal cutpoint above as *Npy1r* expression threshold. (**C**) Top: ROC curve using *Npy1r* expression level in *Npy1r*$^{+}$ neurons to predict Act-FC during fear retrieval and its optimal cutpoint. Bottom: Classification of *Npy1r*$^{+}$ neurons using the optimal cutpoint above as *Npy1r* expression threshold. (**D**) Top: ROC curve using *Pdyn* expression level in *Pdyn*$^{+}$ neurons to predict Act-FC during Eat-Hng and its optimal cutpoint. Bottom: Classification of *Pdyn*$^{+}$ neurons using the optimal cutpoint above as *Pdyn* expression threshold. Precision improvements by using the optimal expression thresholds are shown at the top of bottom panels. Dashed lines in bottom panels: the optimal expression threshold.

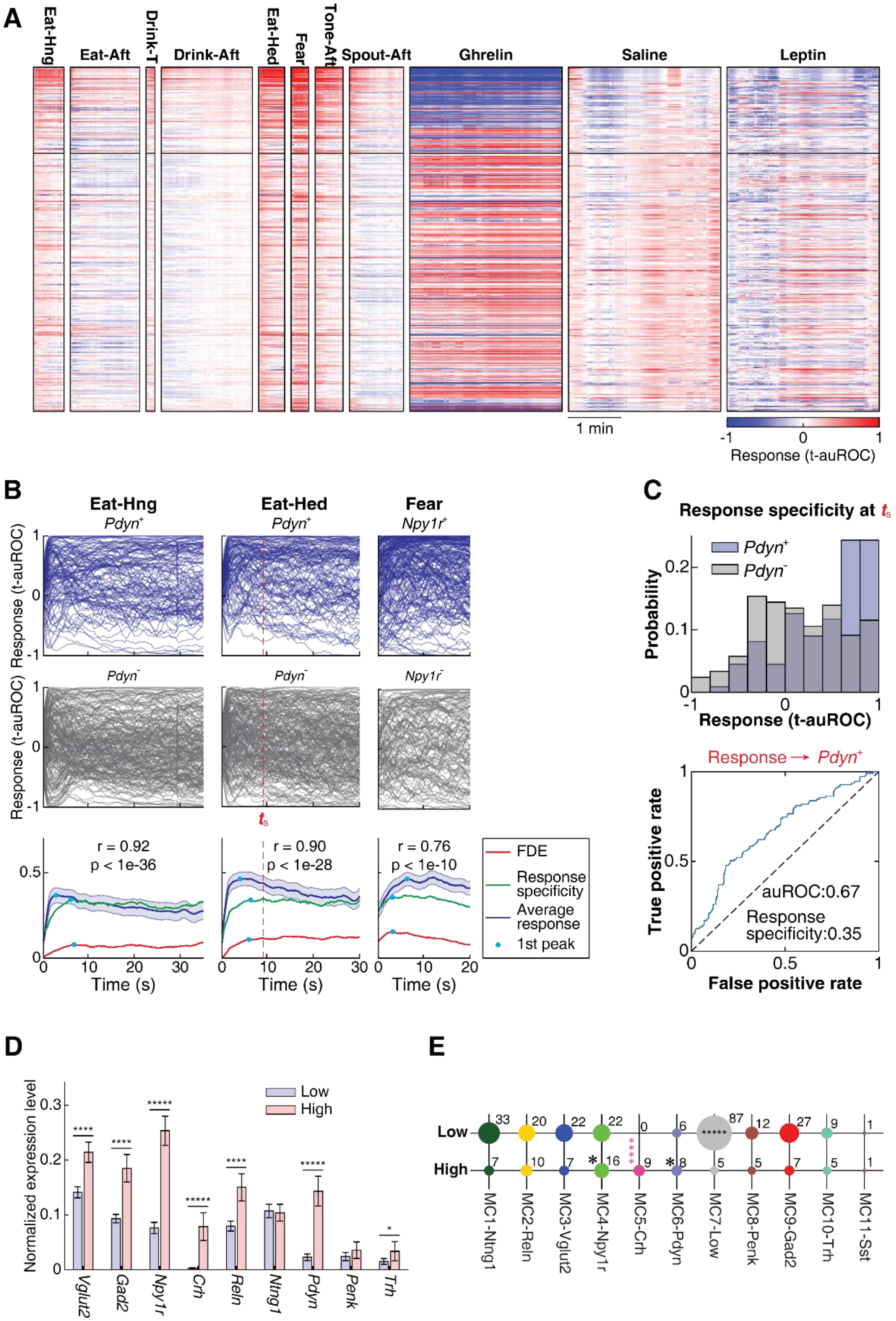

Fig. S33. The relationship between gene expression profiles of individual neurons and their temporal responses in multiple behavioral states.

(**A**) The optimal prediction of temporal responses from mSFFS in Fig. 9A. (**B**) The contributions of cell-type-specific neuronal dynamics to the predictive power of temporal responses. Top row: Response traces of individual *Pdyn*$^+$ neurons in Eat-Hng (left), *Pdyn*$^+$ neurons in Eat-Hed (middle) and *Npy1r*$^+$ neurons in Fear (right). *Pdyn* and *Npy1r* were the most predictive genes for temporal responses in those behavioral states. Middle row: Response traces of individual *Pdyn*$^-$ neurons in Eat-H (left), *Pdyn*$^-$ neurons in Eat-Hed (middle) and *Npy1r*$^-$ neurons in Fear (right). Bottom row: the average responses, response specificities and FDEs of *Pdyn*$^+$ neurons in Eat-Hng (left), *Pdyn*$^+$ neurons in Eat-Hed (middle) and *Npy1r*$^+$ neurons in Fear (right). Definition and calculation of the response specificity of neurons expressing a marker-gene at each timestamp is shown in (C) using *Pdyn*$^+$ neurons at timestamp t_s (red dashed lines) in Eat-Hed as an example. The correlation coefficients (r) between response specificities and FDEs showed high statistical significance, indicating response specificity was critical for predictive accuracy of marker-genes. Shaded blue lines: mean ± SEM. Blue dots: 1st peaks in average responses, response specificities and FDEs. The time differences between the 1st peaks of the average responses and FDEs highlight the different temporal profiles of *xxx*$^+$-neuron response and *xxx*$^+$-neuron-specific response. Thus, the cell-type-specific responses (indicated by the green lines) are important for predictions of temporal dynamics, not necessarily the peak response of the cell type (shaded blue lines) when these responses are also observed in other cell types. (**C**) Response specificity of *Pdyn*$^+$ neurons at t_s (red dashed lines in B). Top: distributions of the responses of *Pdyn*$^+$ and *Pdyn*$^-$ neurons. Bottom: ROC curve using neuronal responses to distinguish *Pdyn*$^+$ neurons from the entire ensemble. Response specificity at each timestamp was defined as the normalized AUC of the ROC curve at that timestamp (normalization: 2*(auROC-0.5)). (**D**) Differences in expression level of marker-genes between the neurons with top quartile of FDEs (High group, red) and other neurons with low FDEs (Low group, blue). Error bars: mean ± SEM. (**E**) Differential enrichment of molecularly defined cell types between High and Low groups. Cell counts are labelled and proportional to circle area. * $p < 0.05$, **** $p < 10^{-4}$, ***** $p < 10^{-5}$. Statistics in Table S2.

Table S1. Spatial organization of molecularly defined cell types in PVH subregions

No.	Cell Type	Anterior	Middle	Posterior
1	C6-Sst	enriched in the PVHpv; along the wall of 3rd ventricle		
2	C9-Crh	scattered	clustered in the PVHmpd	scattered
3	C7-Trh	widely distributed outside of the PVHpv	concentrated in the PVHmpd	widely distributed outside of the PVHpv
4	C3-Avp	rare	widely distributed; formed a densely packed ‘‘ball’’ in the the PVHpml	scattered outside of the PVHmpv
5	C1-Oxt	scattered	clustered in the PVHpv; intermixed with Avp in the PVHpmm	scattered in PVHpv and PVHmpv; intermixed with Avp in the PVHdp
6	C10-Npy1r	scattered	distributed in the PVHmpd, ventral PVHpv and ventral PVHpmm	densely compacted in the PVHmpv
7	C12-Penk	scattered outside of the PVHpv	distributed in the PVHmpd and the PVHpmm	clustered in the PVHlp and the mpv-lp border of the PVHmpv
8	C2-Pdyn	scattered in the PVHpv	distributed outside of the PVHmpd	distributed outside of the PVHpv
9	C11-Ntng1 C13-Reln	widely scattered	widely distributed outside of the PVHpv	densely clustered outside of the PVHpv

Note: Items 1-5 were consistent with immunohistochemistry results reported in a previous study (*55*).

Table S2. Summary of statistical analyses

Figure	Sample size (n)	Statistical Test	Values
**Fig. 2B**	4 samples from 2 mice (left and right)	All passed normal distribution test. Equal variance: one-way ANOVA followed by Tukey multiple comparison test. Unequal variance: one-way Welch's ANOVA followed by Tamhane T2 multiple comparison test	As indicated in Fig. 2B
**Fig. 4B**	Neuron number shown in Fig. 4B	Fisher's exact test	As indicated in Fig. 4B
**Fig. 5G**	MC5-Crh: 225<br>MC6-Pydn: 350	Two-sample Kolmogorov-Smirnov test	As indicated in Fig. 5G
**Fig. 6B**	All: 3509<br>MC1-Ntng1:440<br>MC2-Reln:330<br>MC3-Vglut2:319<br>MC4-Npy1r:418<br>MC5-Crh:99<br>MC6-Pydn:154<br>MC7-Low:1012<br>MC8-Penk:187<br>MC9-Gad2:374<br>MC10-Trh:154	Chi-squared test followed by Marascuillo procedure	As indicated in Fig. 6B
**Fig. 6D**	9	one-way repeated measures ANOVA followed by Tukey-Kramer test	As indicated in Fig. 6D
**Fig. 6G**	2000	Wilcoxon rank sum test	p=0
**Fig. 7C**	*Pdyn*$^+$: 32 (Act-FC) vs. 79 (Inh-FC) neurons;<br>*Crh*$^+$: 23 (Act-FC) vs. 55 (Inh) neurons;<br>*Npy1r*$^+$: 73 (Act-FC) vs. 119 (Inh-FC) neurons from 3 mice	Two-sample Kolmogorov-Smirnov test	*Pdyn*$^+$: $p=5*10^{-3}$<br>*Crh*$^+$: $p=9.6*10^{-3}$<br>*Npy1r*$^+$: $p=2.1*10^{-6}$
**Fig. 7D**	**Eat-Hng**: 151 (Act-FC) vs. 168 (Inh-FC) neurons<br>**Eat-Aft**: 158 (Act-FC) vs. 161 (Inh-FC) neurons	Wilcoxon rank sum test followed by Benjamini and Hochberg false discovery rate adjustment	As indicated in Fig. 7D

	**Drink-T**: 155 (Act-FC) vs. 164 (Inh-FC) neurons<br>**Drink-Aft**: 129 (Act-FC) vs. 190 (Inh-FC) neurons<br>**Eat-Hed**: 155 (Act-FC) vs. 164 (Inh-FC) neurons<br>**Fear**: 184 (Act-FC) vs. 135 (Inh-FC) neurons<br>**Tone-Aft**: 177 (Act-FC) vs. 142 (Inh-FC) neurons<br>**Spout-Aft**: 156 (Act-FC) vs. 163 (Inh-FC) neurons<br>**Ghrelin**: 166 (Act-FC) vs. 153 (Inh-FC) neurons<br>**Saline**: 151 (Act-FC) vs. 168 (Inh-FC) neurons<br>**Leptin**: 161 (Act-FC) vs. 158 (Inh-FC) neurons<br>from 3 mice		
**Fig. 8C**	11	Paired-sample *t*-test	$9.4*10^{-4}$
**Fig. 8E**	*Npy1r*$^+$: 192 neurons<br>*Crh*$^+$: 78 neurons<br>*Pdyn*$^+$: 111 neurons<br>from 3 mice	Chi-squared test	As indicated in Fig. 8E
**Fig. 8F**	11	Paired-sample t-test	$2.3*10^{-3}$
**fig. S5A**	4 samples from 2 mice (left and right)	Normal distribution with equal variance: one-way ANOVA followed by Tukey multiple comparison test.<br>Normal distribution with unequal variance: one-way Welch's ANOVA followed by Tamhane T2 multiple comparison test<br>Non-normal distribution: Kruskal-Wallis test followed by Tukey multiple comparison tests	As indicated in fig. S5A

**fig. S6G**	pPVH: 1394<br>mPVH: 1855<br>pPVH: 1263	Chi-squared test followed by pairwise comparisons with Bonferroni correction	As indicated in fig. S6G
**fig. S13B**	Neuron number shown in fig. S13B	Fisher's exact test	As indicated in fig. S13B
**fig. S14B**	Neuron number shown in fig. S14B	Fisher's exact test	As indicated in fig. S14B
**fig. S19C**	110	paired-sample t-test	$2.8*10^{-11}$
**fig. S20D**	7	paired-sample t-test	$6*10^{-5}$
**fig. S20E**	7	paired-sample t-test	$3*10^{-4}$
**fig. S20F**	As shown in fig. S20F	Chi-squared test	$5.07*10^{-17}$
**fig. S20G**	As shown in fig. S20F	Pairwise Chi-squared test with Bonferroni correction	As indicated in fig. S20G
**fig. S22A**	11	one-way repeated measures ANOVA followed by Tukey-Kramer test	As indicated in fig. S22A
**fig. S23A**	2000	Wilcoxon rank sum test	As indicated in fig. S23A
**fig. S25A**	14	one-sample t-test	As indicated in fig. S25A
**fig. S25C**	17	one-sample t-test	As indicated in fig. S25C
**fig. S27A**	Same as Fig. 7D	Same as Fig. 7D	As indicated in fig. S27A
**fig. S27B**	Same as Fig. 7D	Same as Fig. 7D	As indicated in fig. S27B
**fig. S28A**	As labelled in fig. S28A	Fisher's exact tests followed by Benjamini and Hochberg false discovery rate adjustment	As indicated in fig. S28A
**fig. S28B**	Same as fig. S28A	Same as fig. S28A	As indicated in fig. S28B
**fig. S28C**	Same as fig. S28A	Same as fig. S28A	As indicated in fig. S28C
**fig. S31B**	FC1:92<br>FC2:54<br>FC3:54<br>FC4:75<br>FC5:44	Kruskal-Wallis test followed by Tukey multiple comparison tests	As indicated in fig. S31B
**fig. S33B**	Eat-H: 90<br>Eat-Hed: 78<br>Fear: 52	Pearson Correlation	As indicated in fig. S33B
**fig. S33D**	High: 80<br>Low: 239	Wilcoxon rank sum test followed by Benjamini and Hochberg false discovery rate adjustment	As indicated in fig. S33D

**fig. S33E**	As labelled in fig. S33E	Fisher’s exact tests followed by Benjamini and Hochberg false discovery rate adjustment	As indicated in fig. S33E

Movie. S1. Two-photon-GRIN-lens volumetric imaging of PVH neuron calcium activity during fear retrieval experiment.

PVH neuronal ensemble activity and mouse behaviors during Baseline, Eat-Hed, Fear, Tone-Aft and Spout-Aft states in one trial of the fear retrieval experiment. Top 2 rows show the volumetric calcium imaging of PVH neuron activity. ΔF/F activity (black-to-yellow colormap) is superimposed (alpha = 0.5) with the template reference images (gray) from 8 imaging planes. ΔF/F was calculated for each pixel's time series as previously described (*65*) with minor modification (see Methods). The template reference images are the average intensity projection of the temporal calcium images. To clearly show all neurons in the template images, Gamma of the template images was adjusted to 0.45. Bottom row shows the video of the mouse behaviors (Left: front view; Right: side view). The ensemble activity patterns are visually distinct in different states. The red box signifies the auditory cue (conditioned with electrical foot-shock in a foot-shock arena on the day before the experiment—no shock during the imaging experiment), and the mouse immediately stops licking. Lick suppression continues after the offset of the tone (Tone-Aft state). Video was played back at 6× speed. Audio was played back at 1× speed but trimmed to video playback speed.

References and Notes

1. C. Stringer, M. Pachitariu, N. Steinmetz, C. B. Reddy, M. Carandini, K. D. Harris, Spontaneous behaviors drive multidimensional, brainwide activity. *Science* **364**, 255 (2019). doi:10.1126/science.aav7893 Medline

2. J. E. Allen, T. E. Sutherland, Crystal-clear treatment for allergic disease. *Science* **364**, 738–739 (2019). doi:10.1126/science.aax6175 Medline

3. J. Gründemann, Y. Bitterman, T. Lu, S. Krabbe, B. F. Grewe, M. J. Schnitzer, A. Lüthi, Amygdala ensembles encode behavioral states. *Science* **364**, eaav8736 (2019). doi:10.1126/science.aav8736 Medline

4. X. Chen, Y. Mu, Y. Hu, A. T. Kuan, M. Nikitchenko, O. Randlett, A. B. Chen, J. P. Gavornik, H. Sompolinsky, F. Engert, M. B. Ahrens, Brain-wide organization of neuronal activity and convergent sensorimotor transformations in larval zebrafish. *Neuron* **100**, 876–890.e5 (2018). doi:10.1016/j.neuron.2018.09.042 Medline

5. E. S. Lein, M. J. Hawrylycz, N. Ao, M. Ayres, A. Bensinger, A. Bernard, A. F. Boe, M. S. Boguski, K. S. Brockway, E. J. Byrnes, L. Chen, L. Chen, T.-M. Chen, M. Chi Chin, J. Chong, B. E. Crook, A. Czaplinska, C. N. Dang, S. Datta, N. R. Dee, A. L. Desaki, T. Desta, E. Diep, T. A. Dolbeare, M. J. Donelan, H.-W. Dong, J. G. Dougherty, B. J. Duncan, A. J. Ebbert, G. Eichele, L. K. Estin, C. Faber, B. A. Facer, R. Fields, S. R. Fischer, T. P. Fliss, C. Frensley, S. N. Gates, K. J. Glattfelder, K. R. Halverson, M. R. Hart, J. G. Hohmann, M. P. Howell, D. P. Jeung, R. A. Johnson, P. T. Karr, R. Kawal, J. M. Kidney, R. H. Knapik, C. L. Kuan, J. H. Lake, A. R. Laramee, K. D. Larsen, C. Lau, T. A. Lemon, A. J. Liang, Y. Liu, L. T. Luong, J. Michaels, J. J. Morgan, R. J. Morgan, M. T. Mortrud, N. F. Mosqueda, L. L. Ng, R. Ng, G. J. Orta, C. C. Overly, T. H. Pak, S. E. Parry, S. D. Pathak, O. C. Pearson, R. B. Puchalski, Z. L. Riley, H. R. Rockett, S. A. Rowland, J. J. Royall, M. J. Ruiz, N. R. Sarno, K. Schaffnit, N. V. Shapovalova, T. Sivisay, C. R. Slaughterbeck, S. C. Smith, K. A. Smith, B. I. Smith, A. J. Sodt, N. N. Stewart, K.-R. Stumpf, S. M. Sunkin, M. Sutram, A. Tam, C. D. Teemer, C. Thaller, C. L. Thompson, L. R. Varnam, A. Visel, R. M. Whitlock, P. E. Wohnoutka, C. K. Wolkey, V. Y. Wong, M. Wood, M. B. Yaylaoglu, R. C. Young, B. L. Youngstrom, X. F. Yuan, B. Zhang, T. A. Zwingman, A. R. Jones, Genome-wide atlas of gene expression in the adult mouse brain. *Nature* **445**, 168–176 (2007). doi:10.1038/nature05453 Medline

6. A. Saunders, E. Z. Macosko, A. Wysoker, M. Goldman, F. M. Krienen, H. de Rivera, E. Bien, M. Baum, L. Bortolin, S. Wang, A. Goeva, J. Nemesh, N. Kamitaki, S. Brumbaugh, D. Kulp, S. A. McCarroll, Molecular diversity and specializations among the cells of the adult mouse brain. *Cell* **174**, 1015–1030.e16 (2018). doi:10.1016/j.cell.2018.07.028 Medline

7. A. Zeisel, H. Hochgerner, P. Lönnerberg, A. Johnsson, F. Memic, J. van der Zwan, M. Häring, E. Braun, L. E. Borm, G. La Manno, S. Codeluppi, A. Furlan, K. Lee, N. Skene, K. D. Harris, J. Hjerling-Leffler, E. Arenas, P. Ernfors, U. Marklund, S. Linnarsson, Molecular architecture of the mouse nervous system. *Cell* **174**, 999–1014.e22 (2018). doi:10.1016/j.cell.2018.06.021 Medline

8. B. Tasic, Z. Yao, L. T. Graybuck, K. A. Smith, T. N. Nguyen, D. Bertagnolli, J. Goldy, E. Garren, M. N. Economo, S. Viswanathan, O. Penn, T. Bakken, V. Menon, J. Miller, O.

Fong, K. E. Hirokawa, K. Lathia, C. Rimorin, M. Tieu, R. Larsen, T. Casper, E. Barkan, M. Kroll, S. Parry, N. V. Shapovalova, D. Hirschstein, J. Pendergraft, H. A. Sullivan, T. K. Kim, A. Szafer, N. Dee, P. Groblewski, I. Wickersham, A. Cetin, J. A. Harris, B. P. Levi, S. M. Sunkin, L. Madisen, T. L. Daigle, L. Looger, A. Bernard, J. Phillips, E. Lein, M. Hawrylycz, K. Svoboda, A. R. Jones, C. Koch, H. Zeng, Shared and distinct transcriptomic cell types across neocortical areas. *Nature* **563**, 72–78 (2018). doi:10.1038/s41586-018-0654-5 Medline

9. A. R. Adamantidis, F. Zhang, A. M. Aravanis, K. Deisseroth, L. de Lecea, Neural substrates of awakening probed with optogenetic control of hypocretin neurons. *Nature* **450**, 420–424 (2007). doi:10.1038/nature06310 Medline

10. D. Atasoy, S. M. Sternson, Chemogenetic tools for causal cellular and neuronal biology. *Physiol. Rev.* **98**, 391–418 (2018). doi:10.1152/physrev.00009.2017 Medline

11. C. J. Magnus, P. H. Lee, J. Bonaventura, R. Zemla, J. L. Gomez, M. H. Ramirez, X. Hu, A. Galvan, J. Basu, M. Michaelides, S. M. Sternson, Ultrapotent chemogenetics for research and potential clinical applications. *Science* **364**, eaav5282 (2019). Medline

12. M. Jazayeri, A. Afraz, Navigating the neural space in search of the neural code. *Neuron* **93**, 1003–1014 (2017). doi:10.1016/j.neuron.2017.02.019 Medline

13. K. Hardcastle, S. Ganguli, L. M. Giocomo, Cell types for our sense of location: Where we are and where we are going. *Nat. Neurosci.* **20**, 1474–1482 (2017). doi:10.1038/nn.4654 Medline

14. C. F. Stevens, Neuronal diversity: Too many cell types for comfort? *Curr. Biol.* **8**, R708–R710 (1998). doi:10.1016/S0960-9822(98)70454-3 Medline

15. T. E. Holy, "Yes! We're all individuals!": Redundancy in neuronal circuits. *Nat. Neurosci.* **13**, 1306–1307 (2010). doi:10.1038/nn1110-1306 Medline

16. S. Fusi, E. K. Miller, M. Rigotti, Why neurons mix: High dimensionality for higher cognition. *Curr. Opin. Neurobiol.* **37**, 66–74 (2016). doi:10.1016/j.conb.2016.01.010 Medline

17. A. J. Northcutt, D. R. Kick, A. G. Otopalik, B. M. Goetz, R. M. Harris, J. M. Santin, H. A. Hofmann, E. Marder, D. J. Schulz, Molecular profiling of single neurons of known identity in two ganglia from the crab *Cancer borealis*. *Proc. Natl. Acad. Sci. U.S.A.* **116**, 26980–26990 (2019). doi:10.1073/pnas.1911413116 Medline

18. W. E. Armstrong, "Hypothalamic supraoptic and paraventricular nuclei," in *The Rat Nervous System*, G. Paxinos, Ed. (Elsevier, 2004), pp. 369–388.

19. R. A. Romanov, A. Zeisel, J. Bakker, F. Girach, A. Hellysaz, R. Tomer, A. Alpár, J. Mulder, F. Clotman, E. Keimpema, B. Hsueh, A. K. Crow, H. Martens, C. Schwindling, D. Calvigioni, J. S. Bains, Z. Máté, G. Szabó, Y. Yanagawa, M.-D. Zhang, A. Rendeiro, M. Farlik, M. Uhlén, P. Wulff, C. Bock, C. Broberger, K. Deisseroth, T. Hökfelt, S. Linnarsson, T. L. Horvath, T. Harkany, Molecular interrogation of hypothalamic organization reveals distinct dopamine neuronal subtypes. *Nat. Neurosci.* **20**, 176–188 (2017). doi:10.1038/nn.4462 Medline

20. L. W. Swanson, P. E. Sawchenko, Hypothalamic integration: Organization of the paraventricular and supraoptic nuclei. *Annu. Rev. Neurosci.* **6**, 269–324 (1983). doi:10.1146/annurev.ne.06.030183.001413 Medline

21. M. M. Li, J. C. Madara, J. S. Steger, M. J. Krashes, N. Balthasar, J. N. Campbell, J. M. Resch, N. J. Conley, A. S. Garfield, B. B. Lowell, The paraventricular hypothalamus regulates satiety and prevents obesity via two genetically distinct circuits. *Neuron* **102**, 653–667.e6 (2019). doi:10.1016/j.neuron.2019.02.028 Medline

22. M. J. Krashes, B. P. Shah, J. C. Madara, D. P. Olson, D. E. Strochlic, A. S. Garfield, L. Vong, H. Pei, M. Watabe-Uchida, N. Uchida, S. D. Liberles, B. B. Lowell, An excitatory paraventricular nucleus to AgRP neuron circuit that drives hunger. *Nature* **507**, 238–242 (2014). doi:10.1038/nature12956 Medline

23. T. Füzesi, N. Daviu, J. I. Wamsteeker Cusulin, R. P. Bonin, J. S. Bains, Hypothalamic CRH neurons orchestrate complex behaviours after stress. *Nat. Commun.* **7**, 11937 (2016). doi:10.1038/ncomms11937 Medline

24. C. B. Saper, B. B. Lowell, The hypothalamus. *Curr. Biol.* **24**, R1111–R1116 (2014). doi:10.1016/j.cub.2014.10.023 Medline

25. K. K. Ishii, T. Osakada, H. Mori, N. Miyasaka, Y. Yoshihara, K. Miyamichi, K. Touhara, A labeled-line neural circuit for pheromone-mediated sexual behaviors in mice. *Neuron* **95**, 123–137.e8 (2017). doi:10.1016/j.neuron.2017.05.038 Medline

26. A. K. Graebner, M. Iyer, M. E. Carter, Understanding how discrete populations of hypothalamic neurons orchestrate complicated behavioral states. *Front. Syst. Neurosci.* **9**, 111 (2015). doi:10.3389/fnsys.2015.00111 Medline

27. S. M. Sternson, Hypothalamic survival circuits: Blueprints for purposive behaviors. *Neuron* **77**, 810–824 (2013). doi:10.1016/j.neuron.2013.02.018 Medline

28. D. O. Hebb, *The Organization of Behavior* (Erlbaum, 1949/2002).

29. J. R. Moffitt, D. Bambah-Mukku, S. W. Eichhorn, E. Vaughn, K. Shekhar, J. D. Perez, N. D. Rubinstein, J. Hao, A. Regev, C. Dulac, X. Zhuang, Molecular, spatial, and functional single-cell profiling of the hypothalamic preoptic region. *Science* **362**, eaau5324 (2018). doi:10.1126/science.aau5324 Medline

30. D. Lee, M. Kume, T. E. Holy, Sensory coding mechanisms revealed by optical tagging of physiologically defined neuronal types. *Science* **366**, 1384–1389 (2019). doi:10.1126/science.aax8055 Medline

31. M. Lovett-Barron, R. Chen, S. Bradbury, A. S. Andalman, M. Wagle, S. Guo, K. Deisseroth, Multiple convergent hypothalamus-brainstem circuits drive defensive behavior. *Nat. Neurosci.* **23**, 959–967 (2020). doi:10.1038/s41593-020-0655-1 Medline

32. M. Lovett-Barron, A. S. Andalman, W. E. Allen, S. Vesuna, I. Kauvar, V. M. Burns, K. Deisseroth, Ancestral circuits for the coordinated modulation of brain state. *Cell* **171**, 1411–1423.e17 (2017). doi:10.1016/j.cell.2017.10.021 Medline

33. A. M. Kerlin, M. L. Andermann, V. K. Berezovskii, R. C. Reid, Broadly tuned response properties of diverse inhibitory neuron subtypes in mouse visual cortex. *Neuron* **67**, 858–871 (2010). doi:10.1016/j.neuron.2010.08.002 Medline

34. L. M. Barnett, T. E. Hughes, M. Drobizhev, Deciphering the molecular mechanism responsible for GCaMP6m's Ca2+-dependent change in fluorescence. *PLOS ONE* **12**, e0170934 (2017). doi:10.1371/journal.pone.0170934 Medline

35. C. Li, J. Navarrete, J. Liang-Guallpa, C. Lu, S. C. Funderburk, R. B. Chang, S. D. Liberles, D. P. Olson, M. J. Krashes, Defined paraventricular hypothalamic populations exhibit differential responses to food contingent on caloric state. *Cell Metab.* **29**, 681–694.e5 (2019). doi:10.1016/j.cmet.2018.10.016 Medline

36. J. Kim, S. Lee, Y.-Y. Fang, A. Shin, S. Park, K. Hashikawa, S. Bhat, D. Kim, J.-W. Sohn, D. Lin, G. S. B. Suh, Rapid, biphasic CRF neuronal responses encode positive and negative valence. *Nat. Neurosci.* **22**, 576–585 (2019). doi:10.1038/s41593-019-0342-2 Medline

37. Y. Mandelblat-Cerf, A. Kim, C. R. Burgess, S. Subramanian, B. A. Tannous, B. B. Lowell, M. L. Andermann, Bidirectional anticipation of future osmotic challenges by vasopressin neurons. *Neuron* **93**, 57–65 (2017). doi:10.1016/j.neuron.2016.11.021 Medline

38. J. Y. Cohen, S. Haesler, L. Vong, B. B. Lowell, N. Uchida, Neuron-type-specific signals for reward and punishment in the ventral tegmental area. *Nature* **482**, 85–88 (2012). doi:10.1038/nature10754 Medline

39. J. N. Betley, S. Xu, Z. F. H. Cao, R. Gong, C. J. Magnus, Y. Yu, S. M. Sternson, Neurons for hunger and thirst transmit a negative-valence teaching signal. *Nature* **521**, 180–185 (2015). doi:10.1038/nature14416 Medline

40. Y. Li, A. Mathis, B. F. Grewe, J. A. Osterhout, B. Ahanonu, M. J. Schnitzer, V. N. Murthy, C. Dulac, Neuronal representation of social information in the medial amygdala of awake behaving mice. *Cell* **171**, 1176–1190.e17 (2017). doi:10.1016/j.cell.2017.10.015 Medline

41. G. V. Trunk, A problem of dimensionality: A simple example. *IEEE Trans. Pattern Anal. Mach. Intell.* **1**, 306–307 (1979). doi:10.1109/TPAMI.1979.4766926 Medline

42. A. Kask, L. Rägo, J. Harro, Evidence for involvement of neuropeptide Y receptors in the regulation of food intake: Studies with Y_1-selective antagonist BIBP3226. *Br. J. Pharmacol.* **124**, 1507–1515 (1998). doi:10.1038/sj.bjp.0701969 Medline

43. M. Ranzato, F. J. Huang, Y. Boureau, Y. LeCun, "Unsupervised learning of invariant feature hierarchies with applications to object recognition," in *IEEE Conference on Computer Vision and Pattern Recognition* (IEEE, 2007), pp. 1–8; https://doi.org/10.1109/CVPR.2007.383157.

44. D. H. Hubel, T. N. Wiesel, Receptive fields, binocular interaction and functional architecture in the cat's visual cortex. *J. Physiol.* **160**, 106–154 (1962). doi:10.1113/jphysiol.1962.sp006837 Medline

45. Y. LeCun, I. Kanter, S. A. Solla, "Second order properties of error surfaces: Learning time and generalization," in *Advances in Neural Information Processing Systems 3*, R. P. Lippmann, J. E. Moody, D. S. Touretzky, Eds. (NIPS, 1990), pp. 396–404.

46. H. Zeng, J. R. Sanes, Neuronal cell-type classification: Challenges, opportunities and the path forward. *Nat. Rev. Neurosci.* **18**, 530–546 (2017). doi:10.1038/nrn.2017.85 Medline

47. H. Herzog, 30Years of NPY research. *Neuropeptides* **46**, 251 (2012). doi:10.1016/j.npep.2012.10.002 Medline

48. S. O. Fetissov, J. Kopp, T. Hökfelt, Distribution of NPY receptors in the hypothalamus. *Neuropeptides* **38**, 175–188 (2004). doi:10.1016/j.npep.2004.05.009 Medline

49. R. A. Romanov, A. Alpár, T. Hökfelt, T. Harkany, Molecular diversity of corticotropin-releasing hormone mRNA-containing neurons in the hypothalamus. *J. Endocrinol.* **232**, R161–R172 (2017). doi:10.1530/JOE-16-0256 Medline

50. A. G. Watts, L. W. Swanson, Diurnal variations in the content of preprocorticotropin-releasing hormone messenger ribonucleic acids in the hypothalamic paraventricular nucleus of rats of both sexes as measured by in situ hybridization. *Endocrinology* **125**, 1734–1738 (1989). doi:10.1210/endo-125-3-1734 Medline

51. K. H. Chen, A. N. Boettiger, J. R. Moffitt, S. Wang, X. Zhuang, RNA imaging. Spatially resolved, highly multiplexed RNA profiling in single cells. *Science* **348**, aaa6090 (2015). doi:10.1126/science.aaa6090 Medline

52. S. Shah, E. Lubeck, W. Zhou, L. Cai, In situ transcription profiling of single cells reveals spatial organization of cells in the mouse hippocampus. *Neuron* **92**, 342–357 (2016). doi:10.1016/j.neuron.2016.10.001 Medline

53. J. A. Harris, K. E. Hirokawa, S. A. Sorensen, H. Gu, M. Mills, L. L. Ng, P. Bohn, M. Mortrud, B. Ouellette, J. Kidney, K. A. Smith, C. Dang, S. Sunkin, A. Bernard, S. W. Oh, L. Madisen, H. Zeng, Anatomical characterization of Cre driver mice for neural circuit mapping and manipulation. *Front. Neural Circuits* **8**, 76 (2014). doi:10.3389/fncir.2014.00076 Medline

54. K. Y. Lee, S. J. Russell, S. Ussar, J. Boucher, C. Vernochet, M. A. Mori, G. Smyth, M. Rourk, C. Cederquist, E. D. Rosen, B. B. Kahn, C. R. Kahn, Lessons on conditional gene targeting in mouse adipose tissue. *Diabetes* **62**, 864–874 (2013). doi:10.2337/db12-1089 Medline

55. J. Biag, Y. Huang, L. Gou, H. Hintiryan, A. Askarinam, J. D. Hahn, A. W. Toga, H.-W. Dong, Cyto- and chemoarchitecture of the hypothalamic paraventricular nucleus in the C57BL/6J male mouse: A study of immunostaining and multiple fluorescent tract tracing. *J. Comp. Neurol.* **520**, 6–33 (2012). doi:10.1002/cne.22698 Medline

56. S. Xu, H. Yang, V. Menon, A. L. Lemire, L. Wang, F. E. Henry, S. C. Turaga, S. M. Sternson, Code for CaRMA imaging analysis pipelines, example data, preprocessed multiplex FISH data, and preprocessed CaRMA imaging data for: Behavioral state coding by molecularly defined paraventricular hypothalamic cell type ensembles, GitHub (2020); https://github.com/sternson-lab/CaRMA-imaging.

57. C. M. Hempel, K. Sugino, S. B. Nelson, A manual method for the purification of fluorescently labeled neurons from the mammalian brain. *Nat. Protoc.* **2**, 2924–2929 (2007). doi:10.1038/nprot.2007.416 Medline

58. M. S. Cembrowski, M. G. Phillips, S. F. DiLisio, B. C. Shields, J. Winnubst, J. Chandrashekar, E. Bas, N. Spruston, Dissociable structural and functional hippocampal outputs via distinct subiculum cell classes. *Cell* **174**, 1036 (2018). doi:10.1016/j.cell.2018.07.039 Medline

59. B. Tasic, V. Menon, T. N. Nguyen, T. K. Kim, T. Jarsky, Z. Yao, B. Levi, L. T. Gray, S. A. Sorensen, T. Dolbeare, D. Bertagnolli, J. Goldy, N. Shapovalova, S. Parry, C. Lee, K.

Smith, A. Bernard, L. Madisen, S. M. Sunkin, M. Hawrylycz, C. Koch, H. Zeng, Adult mouse cortical cell taxonomy revealed by single cell transcriptomics. *Nat. Neurosci.* **19**, 335–346 (2016). doi:10.1038/nn.4216 Medline

60. P. Langfelder, S. Horvath, Fast *R* functions for robust correlations and hierarchical clustering. *J. Stat. Softw.* **46**, i11 (2012). doi:10.18637/jss.v046.i11 Medline

61. M. I. Love, W. Huber, S. Anders, Moderated estimation of fold change and dispersion for RNA-seq data with DESeq2. *Genome Biol.* **15**, 550 (2014). doi:10.1186/s13059-014-0550-8 Medline

62. J. Ollion, J. Cochennec, F. Loll, C. Escudé, T. Boudier, TANGO: A generic tool for high-throughput 3D image analysis for studying nuclear organization. *Bioinformatics* **29**, 1840–1841 (2013). doi:10.1093/bioinformatics/btt276 Medline

63. B. B. Avants, C. L. Epstein, M. Grossman, J. C. Gee, Symmetric diffeomorphic image registration with cross-correlation: Evaluating automated labeling of elderly and neurodegenerative brain. *Med. Image Anal.* **12**, 26–41 (2008). doi:10.1016/j.media.2007.06.004 Medline

64. T. W. Chen, N. Li, K. Daie, K. Svoboda, A map of anticipatory activity in mouse motor cortex. *Neuron* **94**, 866–879.e4 (2017). doi:10.1016/j.neuron.2017.05.005 Medline

65. Y. Mu, D. V. Bennett, M. Rubinov, S. Narayan, C.-T. Yang, M. Tanimoto, B. D. Mensh, L. L. Looger, M. B. Ahrens, Glia accumulate evidence that actions are futile and suppress unsuccessful behavior. *Cell* **178**, 27–43.e19 (2019). doi:10.1016/j.cell.2019.05.050 Medline

66. A. Rubin, L. Sheintuch, N. Brande-Eilat, O. Pinchasof, Y. Rechavi, N. Geva, Y. Ziv, Revealing neural correlates of behavior without behavioral measurements. *Nat. Commun.* **10**, 4745 (2019). doi:10.1038/s41467-019-12724-2 Medline

67. A. Tsao, J. Sugar, L. Lu, C. Wang, J. J. Knierim, M.-B. Moser, E. I. Moser, Integrating time from experience in the lateral entorhinal cortex. *Nature* **561**, 57–62 (2018). doi:10.1038/s41586-018-0459-6 Medline

68. K. B. J. Franklin, G. Paxinos, *The Mouse Brain in Stereotaxic Coordinates* (Academic, ed. 4, 2013).
