# Supplementary material for: Behavioral state coding by molecularly defined paraventricular hypothalamic cell type ensembles
Source: Science. Author manuscript; Available in PMC 2025 Mar 26. (PMC11938375; doi:10.1126/science.abb2494)
Supplement: MDAR Reproducibility Checklist [file NIHMS1900648-supplement-MDAR_Reproducibility_Checklist.pdf]

Materials Design Analysis Reporting (MDAR) Checklist for Authors

The MDAR framework establishes a minimum set of requirements in transparent reporting applicable to studies in the life sciences (see Statement of Task: doi:10.31222/osf.io/9sm4x.). The MDAR checklist is a tool for authors, editors and others seeking to adopt the MDAR framework for transparent reporting in manuscripts and other outputs.   Please refer to the MDAR Elaboration Document for additional context for the MDAR framework.

Materials

Antibodies	Yes (indicate where provided: page no/section/legend)	n/a
For commercial reagents, provide supplier name, catalogue number and RRID, if available.		X

Cell materials	Yes (indicate where provided: page no/section/legend)	n/a
**Cell lines:** Provide species information, strain. Provide accession number in repository **OR** supplier name, catalog number, clone number, **OR** RRID		X
**Primary cultures:** Provide species, strain, sex of origin, genetic modification status.		X

Experimental animals	Yes (indicate where provided: page no/section/legend)	n/a
**Laboratory animals:** Provide species, strain, sex, age, genetic modification status. Provide accession number in repository **OR** supplier name, catalog number, clone number, **OR** RRID	Yes. (Mice section on page 2 of Supplementary Materials)	
**Animal observed in or captured from the field:** Provide species, sex and age where possible		X
**Model organisms:** Provide Accession number in repository (where relevant) **OR** RRID		x

Plants and microbes	Yes (indicate where provided: page no/section/legend)	n/a
**Plants:** provide species and strain, unique accession number if available, and source (including location for collected wild specimens)		X
**Microbes:** provide species and strain, unique accession number if available, and source		X

Human research participants	Yes (indicate where provided: page no/section/legend)	n/a
Identify authority granting ethics approval (IRB or equivalent committee(s), provide reference number for approval.		X
Provide statement confirming informed consent obtained from study participants.		X
Report on age and sex for all study participants.		x

Design

**Study protocol**	**Yes (indicate where provided: page no/section/legend)**	**n/a**
For clinical trials, provide the trial registration number **OR** cite DOI in manuscript.		x

**Laboratory protocol**	**Yes (indicate where provided: page no/section/legend)**	**n/a**
Provide DOI or other citation details if detailed step-by-step protocols are available.		x

**Experimental study design (statistics details)**	**Yes (indicate where provided: page no/section/legend)**	**n/a**
State whether and how the following have been done, **or** if they were not carried out.	Yes	
Sample size determination	Yes (end of Mice section on page 2 of Supplementary Materials)	
Randomisation	No (end of Mice section on page 2 of Supplementary Materials)	
Blinding	No (end of Mice section on page 2 of Supplementary Materials)	
Inclusion/exclusion criteria	Yes.<br>Non-neuronal cells were excluded from scRNA-Seq analysis (Analysis of scRNA-Seq data on page 3 of Supplementary Materials)<br>Some neurons were excluded from the CaRMA-imaged neuronal analysis (line 1-2 of p. 9 in main text).<br>*Oxt*, *Avp* and *Sst* genes were excluded from molecular clustering of CaRMA-imaged neurons (Classification of molecular clusters (MCs) from multiplexed FISH data on page 13 of Supplementary Materials).<br>Cell type with high *Sst* expression was excluded from behavioral state decoding (Figure legend 5B of main text and Effect of the number of molecular clusters on the robustness of behavioral state decoding on page 17 of Supplementary Materials)	

**Sample definition and in-laboratory replication**	**Yes (indicate where provided: page no/section/legend)**	**n/a**
State number of times the experiment was replicated in laboratory	Yes. 3 mice were used for behavioral and imaging experiments (Figure legend 5A).	
Define whether data describe technical or biological replicates	Yes. (Mice section on page 2 of Supplementary Materials and Table S2 on page 78-81 of Supplementary Materials)	

**Ethics**	**Yes (indicate where provided: page no/section/legend)**	**n/a**
Studies involving human participants: State details of authority granting ethics approval (IRB or equivalent committee(s), provide reference number for approval.		x
Studies involving experimental animals: State details of authority granting ethics approval (IRB or equivalent committee(s), provide reference number for approval.	Yes (page 2 of Supplementary Materials)	
Studies involving specimen and field samples: State if relevant permits obtained, provide details of authority approving study; if none were required, explain why.		x

**Dual Use Research of Concern (DURC)**	**Yes (indicate where provided: page no/section/legend)**	**n/a**
If study is subject to dual use research of concern, state the authority granting approval and reference number for the regulatory approval		x

Analysis

**Attrition**	**Yes (indicate where provided: page no/section/legend)**	**n/a**
State if sample or data point from the analysis is excluded, and whether the criteria for exclusion were determined and specified in advance.		x

**Statistics**	**Yes (indicate where provided: page no/section/legend)**	**n/a**
Describe statistical tests used and justify choice of tests.	Yes (Table S2 on page 78-81 of Supplementary Materials)	

**Data Availability**	**Yes (indicate where provided: page no/section/legend)**	**n/a**
State whether newly created datasets are available, including protocols for access or restriction on access.	Yes (**Data and materials availability** on page. 37-38 of main text)	
If data are publicly available, provide accession number in repository or DOI or URL.	Yes. https://github.com/sternson-lab/CaRMA-imaging GEO submission ID for the single-cell PVH data is GSE148568. (**Data and materials availability** p. 37-38 of main text)	
If publicly available data are reused, provide accession number in repository or DOI or URL, where possible.		x

**Code Availability**	**Yes (indicate where provided: page no/section/legend)**	**n/a**
For all newly generated code and software essential for replicating the main findings of the study:	Yes (**Data and materials availability** on page. 37-38 of main text).	
State whether the code or software is available.	Yes (**Data and materials availability** on page. 37-38 of	
If code is publicly available, provide accession number in repository, or DOI or URL.	Yes (**Data and materials availability** on page. 37-38 of main text). https://github.com/sternson-lab/CaRMA-	

Reporting

**Adherence to community standards**	**Yes (indicate where provided: page no/section/legend)**	**n/a**
MDAR framework recommends adoption of discipline-specific guidelines, established and endorsed through community initiatives. Journals have their own policy about requiring specific guidelines and recommendations to complement MDAR.		x
State if relevant guidelines (eg., ICMJE, MIBBI, ARRIVE) have been followed, and whether a checklist (eg., CONSORT, PRISMA, ARRIVE) is provided with the manuscript.		x
